# The Flexibility of Tetra(N‐Heterocyclic Carbene) Ligands Controls the Nuclearity and Geometry of Polynuclear M^I^‒NHC Assemblies

**DOI:** 10.1002/anie.202502081

**Published:** 2025-05-19

**Authors:** Guang‐Feng Jin, Fang Wang, F. Ekkehardt Hahn, Ying‐Feng Han

**Affiliations:** ^1^ Xi'an Key Laboratory of Functional Supramolecular Structure and Materials College of Chemistry and Materials Science Northwest University Xi'an 710127 P.R. China; ^2^ Institut für Anorganische und Analytische Chemie Westfälische Wilhelms‐Universität Münster Corrensstrasse 30 48149 Münster Germany

**Keywords:** Anthracene, N‐Heterocyclic carbene, Steric hindrance, Supramolecular chemistry

## Abstract

A series of tetrakisimidazolium salts bearing two di(phenylimidazolium)amine groups linked by differently substituted anthracenes has been prepared. These are H_4_‐**1a**(PF_6_)_4_ (anthracene bridge), H_4_‐**1b**(PF_6_)_4_ (phenyl‐anthracene‐phenyl bridge), H_4_‐**1c**(PF_6_)_4_ (anthracene‐phenyl bridge), and H_4_‐**1d**(PF_6_)_4_ (anthracene‐phenyl‐anthracene bridge). X‐ray crystallography showed that those ligand precursors having the di(phenylimidazolium)amine connected directly to the anthracene experience restricted rotation about the N─C_anthracene_ bond. Depending on their flexibility, the reaction of the tetrakisimidazolium salts with Ag_2_O followed by transmetalation with [AuCl(THT)] yielded octanuclear ([Au_8_(**1a**)_4_](PF_6_)_8_), tetranuclear ([Au_4_(**1b**)_2_](PF_6_)_4_), hexanuclear ([Au_6_(**1c**)_3_](PF_6_)_6_), or octanuclear ([Au_8_(**1d**)_4_](PF_6_)_8_) assemblies, demonstrating the direct bonding strategy can be employed for the selective synthesis of polynuclear poly‐NHC (NHC = N‐heterocyclic carbene) metallosupramolecular assemblies.

## Graphical abstract

Tetracarbene ligands composed of two di(phenylimidazol‐2‐ylidene)amine groups linked by an anthracene bridge exhibit restricted rotation of the C_phenyl_─N─C_phenyl_ plane relative to the anthracene moiety if the N(Ph─NHC)_2_ moieties are directly attached to anthracene. The presence or absence of this restriction has been used in coordination driven self‐assembly to prepare discrete M_4_L_2_, M_6_L_3_ and M_8_L_4_ (M = Ag^+^, Au^+^) assemblies.

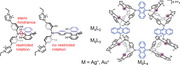

## Introduction

Metallosupramolecular chemistry has developed into an interdisciplinary research field covering a broad range of architectures with intriguing properties.^[^ ^1^, ^2^, ^3^ ^]^ Consequently, metallosupramolecular assemblies have found a wide range of applications in catalysis,^[^ ^4^, ^5^, ^6^, ^7^, ^8^, ^9^ ^]^ as luminescent materials,^[^ ^10^, ^11^, ^12^, ^13^, ^14^ ^]^ in molecular recognition and separation,^[^ ^15^, ^16^, ^17^, ^18^, ^19^, ^20^, ^21^, ^22^, ^23^, ^24^, ^25^, ^26^, ^27^ ^]^ stabilization of reactive molecules,^[^ ^28^, ^29^, ^30^, ^31^, ^32^ ^]^ drug delivery,^[^ ^33^, ^34^, ^35^ ^]^ and molecular weaving.^[^ ^36^, ^37^, ^38^, ^39^ ^]^

Coordination‐driven self‐assembly constitutes the classical approach for the synthesis of diverse metallosupramolecular architectures.^[^ ^1^, ^2^, ^3^, ^40^, ^41^, ^42^, ^43^, ^44^, ^45^, ^46^ ^]^ The highly directional and predictable nature of the metal‒ligand coordination sphere is a critical feature of coordination‐driven self‐assembly where the information encoded in the building blocks determines the outcome of the assembly reaction. Among the self‐assembly reactions based on metal–ligand coordination, directional bonding,^[^ ^1^, ^47^, ^48^ ^]^ symmetry interaction,^[^ ^49^ ^]^ molecular paneling,^[^ ^50^ ^]^ and the weak link^[^ ^51^, ^52^ ^]^ constitute the most common strategies.

Most metallosupramolecular assemblies have been constructed from transition metal ions and Werner‐type ligands featuring N‐, O‐, S‐ or P‐donor ligands. Recently, polydentate ligands featuring C‐donor ligands have been employed for the generation of metallosupramolecular assemblies.^[^ ^53^, ^54^, ^55^, ^56^, ^57^, ^58^, ^59^ ^]^ Most of these organometallic assemblies are built from poly‐NHC ligands, although some exceptions are known.^[^ ^57^, ^58^, ^59^ ^]^ Poly‐NHC (NHC = N‐heterocyclic carbene) ligands in combination with silver ions constitute a perfect combination for the generation of metallosupramolecular structures based on the facile generation of the C_NHC_─Ag bond and the lability of this bond allowing for the formation of the thermodynamically most stable assembly.^[^ ^60^, ^61^ ^]^

**Figure 1:**
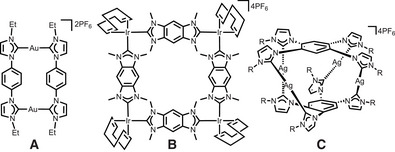
Selected metallosupramolecular structures from poly‐NHC ligands.

Metallosupramolecular assemblies prepared from poly‐NHC ligands are normally obtained following the directional bonding strategy.^[^ ^1^, ^47^, ^48^ ^]^ This approach requires that the precursor units must be able to adopt the required orientation of the donor and acceptor groups for a specific geometry of the assembly and the appropriate stoichiometric ratio of the precursors must be used. For example, a dicarbene ligand with angular orientation of the NHC donors of 0° reacts with Ag^+^ ions followed by transmetalation to Au^+^ (linear coordination mode) to yield the metallarectangle **A** (Figure 1).^[^ ^62^, ^63^ ^]^ However, the reaction of a benzobiscarbene ligand featuring an angular orientation of the donor groups of 180° with square‐planar coordinated Ir^I^ (two cis‐coordination sites) yields molecular square **B**.^[^ ^64^ ^]^ The molecular cylinder **C** was obtained from a benzene bridged tetracarbene ligand and Ag^+^ ions.^[^ ^65^, ^66^ ^]^ Although the poly‐NHC ligands in **A** and **C** possess some flexibility regarding the rotation about the N_NHC_─C_phenyl_ bond, the linear coordination mode of the C_NHC_─M─C_NHC_ bonds enforces the formation of the dinuclear metallarectangle and the tetranuclear cylinder.

Using the directional bonding strategy, the spatial orientation of the donor groups and geometric constraints of polydentate ligands can shape the self‐assembly of high‐symmetry structures. The effect of minute changes in the bonding angle or the flexibility of bidentate ligands has been demonstrated.^[^ ^67^, ^68^ ^]^ For example, Fujita et al. have shown that selected bipyridines L in combination with Pd^2+^ ions can react with formation of various polyhedra of type M_n_L_2n_ with *n* = 3,^[^ ^69^ ^]^ 6,^[^ ^70^ ^]^ 12,^[^ ^71^ ^]^ 24,^[^ ^72^, ^73^ ^]^ and 60.^[^ ^74^ ^]^

**Scheme 1:**
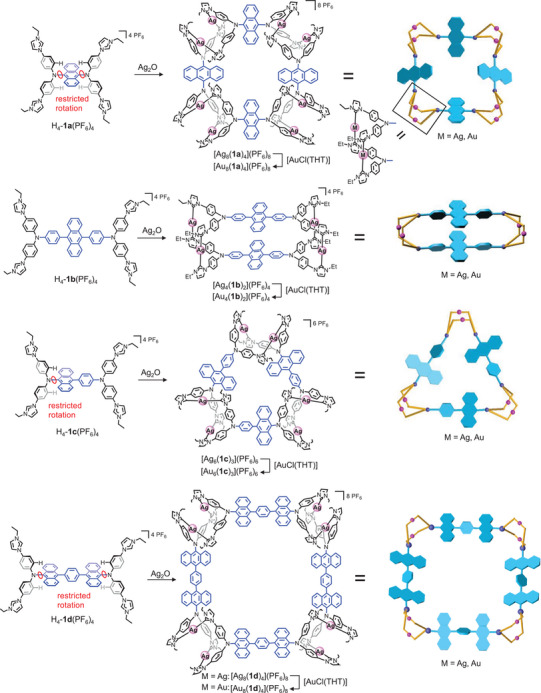
Synthesis of tetra‐, hexa‐, and octanuclear assemblies.

We attempted to introduce steric constraints into tetra‐NHC ligands and thus to influence the nuclearity and geometry of the assemblies obtained from such ligands and M^+^ (M = Ag, Au) ions. Because of the free rotation about the N_NHC_─C_phenyl_ bond in complexes of type **C** and related tetraphenylethylene derivatives,^[^ ^75^ ^]^ these tetra‐NHC ligands form with Ag^+^ ions normally cylinder‐like assemblies of type Ag_4_L_2_. In this study we used 9,10‐substituted anthracene as central bridging unit for the synthesis of novel tetra‐NHC ligands. If di(phenylimidazolium)amine groups are attached directly to the 9 and/or 10 positions of anthracene, the rotation about the C_anthracene_─N bond is restricted as in ligand precursors H_4_‐**1a**(PF_6_)_4_ and H_4_‐**1d**(PF_6_)_4_ (Scheme 1). However, if phenyl spacers are placed in between the anthracene and the di(phenylimidazolium)amine, rotation about both the C_anthracene_─C_phenyl_^[^ ^76^, ^77^ ^]^ and the C_phenyl_─N bonds (ligand precursor H_4_‐**1b**(PF_6_)_4_ in Scheme 1) is possible. Ligand precursor H_4_‐**1c**(PF_6_)_4_ features both, a rotation‐restricted N─C_anthracene_ and a less rotation‐restricted N─C_phenyl_ bond (Scheme 1). Herein, we describe how the modulation of the steric hindrance in ligand precursors H_4_‐**1a**(PF_6_)_4_−H_4_‐**1d**(PF_6_)_4_ leads in the reaction with Ag^+^ ions to the selective formation of tetra‐, hexa‐, or octanuclear assemblies M_4_L_2_, M_6_L_3_, and M_8_L_4_ with various shapes. Transmetalation of the tetra‐NHC ligands to Au^+^ without destruction of the metallosupramolecular framework is also demonstrated.

## Results and Discussion

The tetra‐NHC precursor H_4_‐**1a**(PF_6_)_4_ was synthesized by the reaction of compound *N,N,N*’*,N*’‐tetrakis(4‐bromophenyl)‐*p*‐(9,10‐anthracene)diamine with imidazole, followed by *N*‐alkylation of the imidazole with ethyl bromide and counterion exchange with an excess of NH_4_PF_6_ (Scheme S1). The formation of H_4_‐**1a**(PF_6_)_4_ was confirmed by NMR spectroscopy and HR‐ESI mass spectrometry (Figures S1–S6). An X‐ray diffraction study (Table S1, Figure S47) demonstrates the strain existing within the centrosymmetric cation H_4_‐**1a** ^4+^ with the plane C_phenyl_─N─C_phenyl_ rotated by 72.26° relative to the plane of the central anthracene ring.

**Figure 2:**
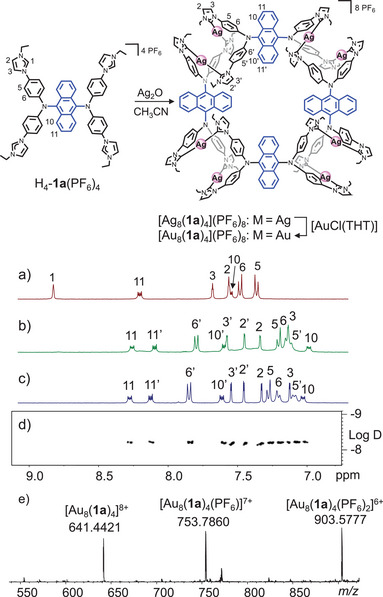
a) ^1^H NMR spectrum (400 MHz, CD_3_CN) of H_4_‐**1a**(PF_6_)_4_. b) ^1^H NMR spectrum (400 MHz, CD_3_CN) of assembly [Ag_8_(**1a**)_4_](PF_6_)_8_. c) ^1^H NMR spectrum (400 MHz, CD_3_CN) of assembly [Au_8_(**1a**)_4_](PF_6_)_8_. d) ^1^H DOSY spectrum (400 MHz, CD_3_CN) of [Au_8_(**1a**)_4_](PF_6_)_8_. e) HR‐ESI mass spectrum of [Au_8_(**1a**)_4_](PF_6_)_8_.

Treatment of tetra‐NHC precursor H_4_‐**1a**(PF_6_)_4_ with an excess of Ag_2_O in CH_3_CN afforded the octanuclear assembly [Ag_8_(**1a**)_4_](PF_6_)_8_ in good yield of 85%. Complete deprotonation of the ligand precursor was concluded from the absence of the resonance for proton H1 in the ^1^H NMR spectrum (Figures 2b, S7), which was observed for the ligand precursor H_4_‐**1a**(PF_6_)_4_ at *δ* = 8.83 ppm (Figures 2a, S1). Two sets of resonances were observed due to the slightly different arrangements of the ligands in the assembly. The HR‐ESI mass spectrum (Figure S8) confirmed the formation of the octanuclear assembly with the strongest peaks observed at *m*/*z* = 552.3891 (calcd for [Ag_8_(**1a**)_4_]^8+^ 552.3747), 652.0094 (calcd for [Ag_8_(**1a**)_4_(PF_6_)]^7+^ 651.9946), and 784.8342 (calcd for [Ag_8_(**1a**)_4_(PF_6_)_2_]^6+^ 784.8210).

Transmetalation of the tetra‐NHC ligands from Ag^+^ to Au^+^ yielded assembly [Au_8_(**1a**)_4_](PF_6_)_8_. Although an informative ^13^C NMR spectrum of [Ag_8_(**1a**)_4_](PF_6_)_8_ could not be recorded, the ^13^C{^1^H] NMR spectrum of [Au_8_(**1a**)_4_](PF_6_)_8_ clearly shows the resonances for the gold coordinated C_NHC_ atoms in the expected range at *δ* = 183.3 and 182.5 ppm (Figure S11). Again, two sets of resonances were observed in the ^1^H (Figure 2c) and ^13^C{^1^H} NMR spectra. ^1^H VT NMR spectroscopy showed no changes of the spectra in the range of 298 to 343 K (Figure S10).The ^1^H DOSY spectrum confirmed that all resonances belong to the same assembly (Figure 2d). The HR‐ESI mass spectrum (Figures 2e, S15) confirmed the formation of the assembly with the strongest peaks observed at *m*/*z* = 641.4421 (calcd for [Au_8_(**1a**)_4_]^8+^ 641.4316), 753.7860 (calcd for [Au_8_(**1a**)_4_(PF_6_)]^7+^ 753.7790), and 903.5777 (calcd for [Au_8_(**1a**)_4_(PF_6_)_2_]^6+^ 903.5695).

**Figure 3:**
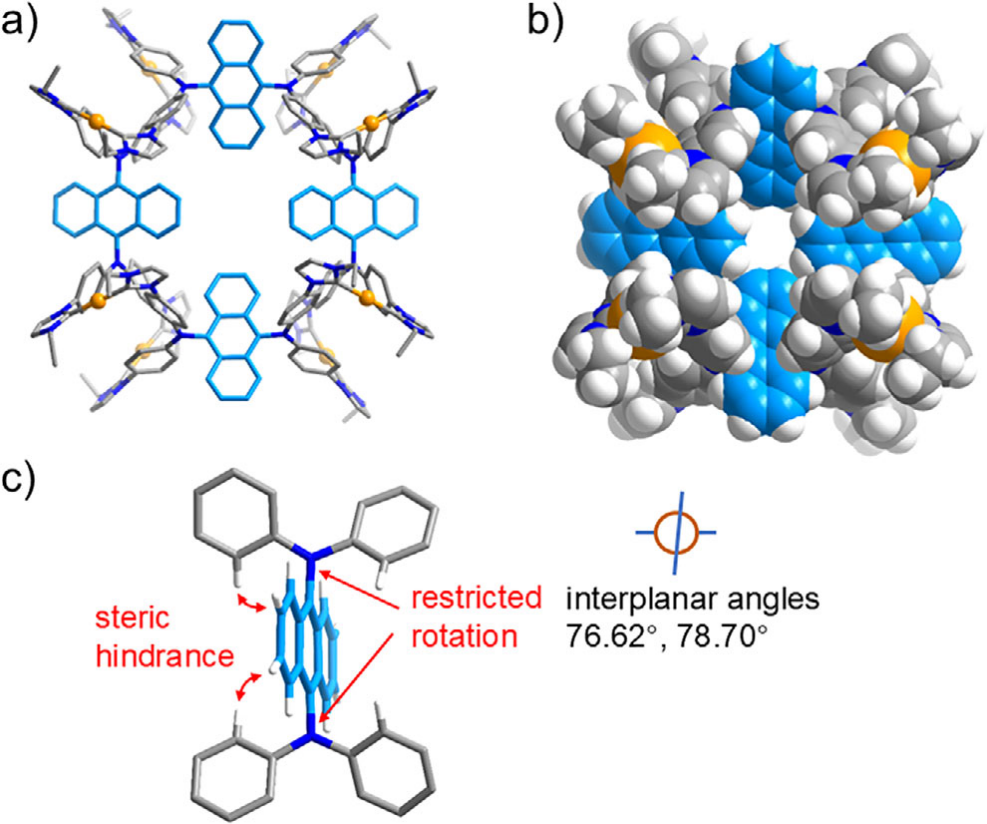
a) Molecular structure of cation [Au_8_(**1a**)_4_]^8+^ in [Au_8_(**1a**)_4_](PF_6_)_8_·6Et_2_O·2CH_3_CN, top view. b) Space‐filling model of cation [Au_8_(**1a**)_4_]^8+^. c) Representation of one of the Ph_2_N–anthracene–NPh_2_ units showing the reasons for the restricted rotation about the anthracene C9─ or C10─NPh_2_ bond. Color code: Au, orange; N, blue; and H, white; carbon atoms of the assemblies are displayed in different colors (light blue and gray) to highlight the central anthracene linkers.

Crystals [Au_8_(1a)_4_](PF_6_)_8_·6Et_2_O·2CH_3_CN were obtained by slow diffusion of diethyl ether into an acetonitrile solution of the assembly at 25 °C. The X‐ray diffraction analysis (Table S2 and Figure S48) confirmed the formation of an assembly of type [Au_8_(1a)_4_]^8+^, where four tetracarbene ligands bridge eight Au^+^ ions (Figure 3a,b). The eight angles between the planes of the anthracene bridges and the C_phenyl_─N─C_phenyl_ planes attached to it fall in a narrow range of 76.62°‒78.70°. These values are very close to the equivalent angles in the tetrakisimidazolium starting material H_4_‐1a(PF_6_)_4_ (72.26°). An inspection of the closest contacts between anthracene hydrogen atoms and the hydrogen atoms of the N‐phenyl rings reveals a separation of 3.491 Å in ligand precursor H_4_‐1a(PF_6_)_4_. For octacation [Au_8_(1a)_4_]^8+^ the shortest separations between anthracene protons and the phenyl protons (total of 16 distances) fall in the range of 3.109‒4.284 Å. These parameters indicate that the rotation about the C_anthracene_─N(C_phenyl_)_2_ bonds is restricted (Figure 3c) and thus very likely responsible for the shape and nuclearity of the [Au_8_(1a)_4_]^8+^ assembly obtained.

In addition, six diethyl ether molecules were found in the asymmetric unit. Two of these are located inside the assembly where they form intermolecular CH_3_─ H⋯ O interactions of 2.65 and 2.76 Å (Figure S48c).

A different picture emerges if tetra‐NHC precursor H_4_‐**1b**(PF_6_)_4_ is considered. This ligand precursor features a phenyl bridge between the anthracene and the N(phenylimidazolium)_2_ moieties (for analytical details see Figures S16–S18). An X‐ray diffraction study with crystals H_4_‐**1b**(PF_6_)_4_ reveals an almost coplanar arrangement of the C_phenyl_─N─C_phenyl_ and anthracene planes (Figure S49, interplanar angle 10.29°. This is possible due to free rotation about the C_anthracene_─C_phenyl_ and the C_phenyl_─NPh_2_ bonds and contrary to H_4_‐**1a**(PF_6_)_4_ no strain exists in this ligand precursor.

The reaction of H_4_‐**1b**(PF_6_)_4_ with an excess of Ag_2_O under conditions identical to those used for the synthesis of [Ag_8_(**1a**)_4_](PF_6_)_8_ yielded the tetranuclear assembly [Ag_4_(**1b**)_2_](PF_6_)_4_ (Scheme 1, for analytical details see Figures S19–S21). Formation of the tetranuclear assembly was confirmed by NMR spectroscopy and HR‐ESI mass spectrometry showing the strongest peak at *m*/*z* = 628.4010 (calcd for [Ag_4_(**1b**)_2_]^4+^ 628.4062). The formation of the tetranuclear assembly must be a result of the facile rotation about the C_anthracene_─C_phenyl_ and the C_phenyl_─NPh_2_ bonds in both the ligand precursor and the assembly obtained from this precursor.

**Figure 4:**
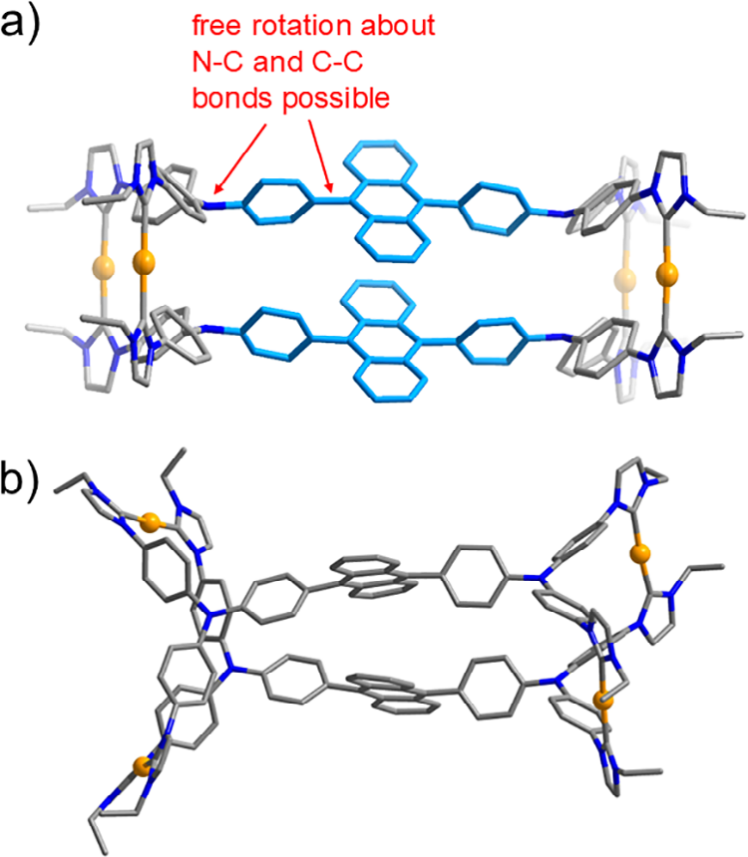
a) Molecular structure of one of the disordered tetracations [Au_4_(**1b**)_2_]^4+^ in [Au_4_(**1b**)_2_](PF_6_)_2.8_(SbF_6_)_1.2_ (determined by X‐ray diffraction). b) Molecular structure of tetracation [Au_4_(**1b**)_2_]^4+^ calculated by molecular modelling with Gaussian.

Multiple attempts to crystallize [Ag_4_(**1b**)_2_](PF_6_)_4_ proceeded without success. Ligand transfer from [Ag_4_(**1b**)_2_](PF_6_)_4_ to [AuCl(THT)] yielded [Au_4_(**1b**)_2_](PF_6_)_4_ with retention of the tetranuclear assembly as was confirmed by NMR spectroscopy (Figures S22,S23) and by HR‐ESI mass spectrometry (Figure S24) showing the strongest peak at *m*/*z* = 717.4595 (calcd for [Au_4_(**1b**)_2_]^4+^ 717.4669). However, all attempts to obtain crystals of the gold compound also failed. Addition of NaSbF_6_ resulted in the formation of weakly scattering crystals of composition [Au_4_(**1b**)_2_](PF_6_)_2.8_(SbF_6_)_1.2_. The X‐ray diffraction analysis with these crystals revealed a strongly disordered [Au_4_(**1b**)_2_]^4+^ cation residing on an inversion center. The diffraction data are only sufficient to confirm the overall geometry of the tetracation. One of the disordered cations is depicted in Figure 4a. The interplanar angle between the anthracene and the C_phenyl_─N─C_phenyl_ planes in the depicted structure measures 33.62° and is much smaller than the equivalent angle in the octanuclear cation [Au_8_(**1a**)_4_]^8+^. Apparently, the facile rotation of the C_phenyl_─N─C_phenyl_ planes relative to the anthracene plane allows for the formation of the tetranuclear cation.

In the absence of reliable X‐ray diffraction data, molecular modelling using Gaussian^[^ ^78^ ^]^ was employed to confirm the molecular structure of cation [Au_4_(**1b**)_2_]^4+^ (Figure 4b). The calculations also reveal a strain‐free tetranuclear cation [Au_4_(**1b**)_2_]^4+^. In the absence of packing and anion effects, however, the angles between the anthracene plane and the C_phenyl_─N─C_phenyl_ planes fall in the range of 44.45°−93.79°. This observation serves as confirmation for the ability of the C_phenyl_─N─C_phenyl_ planes to rotate freely relative to the anthracene planes in the tetranuclear cation.

Given the relevance of free rotation of the anthracene group relative to the C_phenyl_─N─C_phenyl_ planes in the previously discussed octanuclear and tetranuclear assemblies, its effect was further studied. For that purpose, tetra‐NHC ligand precursor H_4_‐**1c**(PF_6_)_4_ was prepared (Scheme 1, Figures S25–S30). This ligand precursor features one N(phenyimidazolium)_2_ group directly linked to anthracene (as in ligand precursor H_4_‐**1a**(PF_6_)_4_) and one N(phenyimidazolium)_2_ group linked to anthracene via an additional phenyl bridge, leading to restricted rotation in the former and free rotation of the latter C_phenyl_─N─C_phenyl_ groups relative to the anthracene group.

An X‐ray diffraction study with crystals of composition H_4_‐**1c**(PF_6_)_4_⋅2CH_3_CN revealed a tetracation where the C_phenyl_─N─C_phenyl_ plane of the N(phenylimidazolium)_2_ group directly linked to the anthracene is rotated relative to the anthracene by 72.72° (Figure S51). This value is almost identical to the corresponding value in tetracation H_4_‐**1a** ^4+^ (72.26°) indicating a restricted rotation about the C_anthracene_─NPh_2_ bond. Interestingly, the C_phenyl_─N─C_phenyl_ plane of the N(phenylimidazolium)_2_ group linked via a phenyl group to anthracene is rotated by 77.57° relative to the anthracene plane. However, this cannot be the result of intramolecular steric interactions as the equivalent interplanar angle in ligand precursor H_4_‐**1b**(PF_6_)_4_ measures only 10.29°. In fact, the observation of drastically different interplanar angles between the anthracene group and the C_phenyl_─N─C_phenyl_ plane of the N(phenylimidazolium)_2_ group when these are bridged by a phenyl group confirms that these two planes are free to rotate relative to each other when not directly connected.

Ligand precursor H_4_‐**1c**(PF_6_)_4_ was reacted with an excess of Ag_2_O in dry acetonitrile as described for the synthesis of [Ag_8_(**1a**)_4_](PF_6_)_8_ and [Ag_4_(**1b**)_2_](PF_6_)_4_ but yielded an assembly with composition [Ag_6_(**1c**)_3_](PF_6_)_6_. Although the ^1^H NMR spectrum was not informative (Figure S31), the formation of a hexanuclear assembly was concluded from the HR‐ESI mass spectrum (Figure S32) showing the strongest peak at *m*/*z* = 590.4851 (calcd for [Ag_6_(**1c**)_3_]^6+^ 590.4738). The transmetalation reaction with [AuCl(THT)] yielded the hexagold assembly [Au_6_(**1c**)_3_](PF_6_)_6_. Although the ^1^H NMR spectrum of this compound is also uninformative (Figure S33), the HR‐ESI mass spectrum (Figure S34) and the ^1^H DOSY NMR spectrum (Figure S35) confirm the successful transmetalation with retention of a hexanuclear assembly.

**Figure 5:**
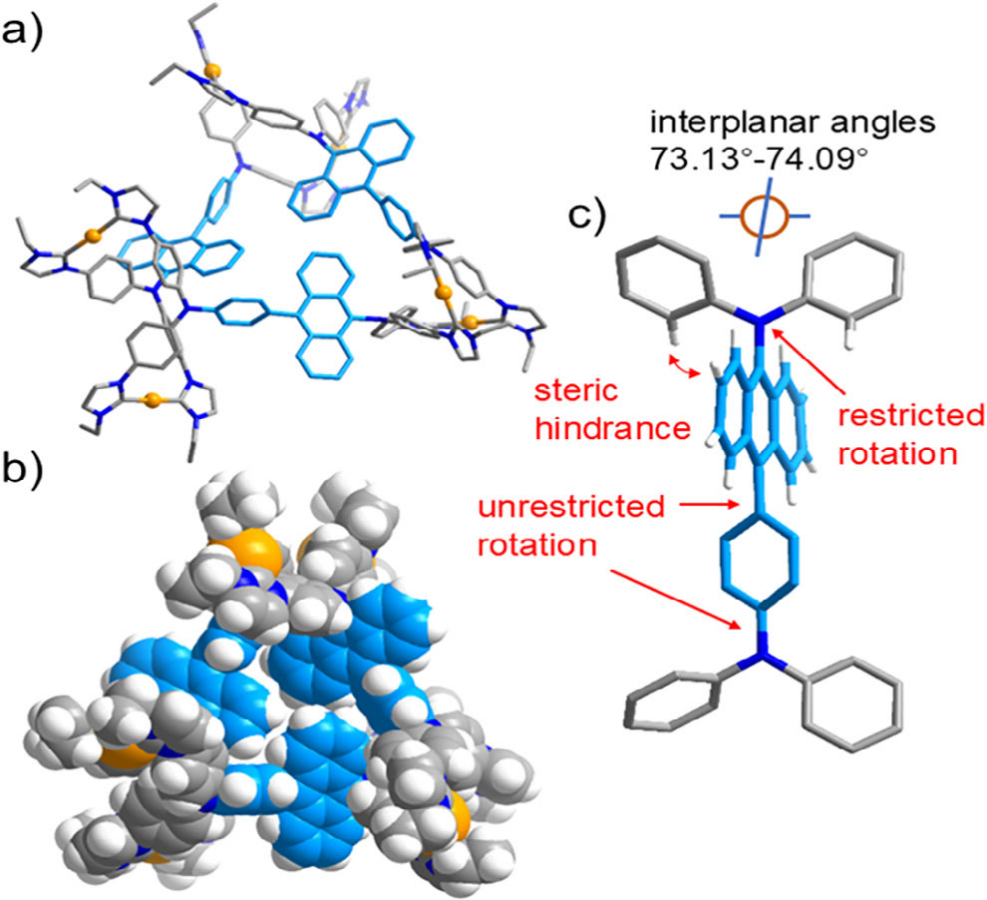
a) Molecular structure of the [Au_6_(**1c**)_3_]^6+^ cation in [Au_6_(**1c**)_3_](PF_6_)_6_. b) Space filling representation of cation [Au_6_(**1c**)_3_]^6+^. c) Representation of one of the three Ph_2_N─anthracene─Ph─NPh_2_ units showing the restricted rotation about the anthracene─NPh_2_ bond.

Final proof for the formation of a hexanuclear assembly was established by an X‐ray diffraction study with crystals of composition [Au_6_(**1c**)_3_](PF_6_)_6_ obtained by slow diffusion of diethyl ether into a saturated acetonitrile solution of the compound. The structure analysis confirmed the presence of a unique hexacation [Au_6_(**1c**)_3_]^6+^ in the asymmetric unit (Figure 5a,b). As expected, the C_phenyl_─N─C_phenyl_ planes of the N(phenylimidazol‐2‐ylidene)_2_ groups directly attached to anthracene are fixed relative to the anthracene planes they are attached to at interplanar angles ranging from 73.13° to 74.09°. These values are almost identical to the equivalent interplanar angles in the ligand precursor H_4_‐**1c**(PF_6_)_4_ (72.72°) confirming the hindered rotation about the N─C_anthracene_ bonds. Contrary to this situation, the C_phenyl_─N─C_phenyl_ planes of the N(phenylimidazol‐2‐ylidene)_2_ groups linked via a phenyl group to anthracene adopt interplanar angles between 29.78° and 89.54° relative to the anthracene plane, indicating that no restriction regarding the orientation of these planes exists. We attribute the formation of the hexanuclear assembly to the combination of free and restricted rotation of the C_phenyl_─N─C_phenyl_ planes relative to the anthracene planes which does not exist in either the octanuclear (restricted rotation) or tetranuclear (no restriction on rotation) assemblies.

Interestingly, the tetra‐NHC ligands adopt a conformation where anthracene protons engage in CH⋯phenyl π‐ interactions with adjacent phenyl groups (distances 3.04, 3.07, and 3.16 Å, see Figure S52c). These interactions might further assist in stabilizing the hexanuclear assembly.

At this point the ability of the C_phenyl_─N─C_phenyl_ plane of the N(phenylimidazol‐2‐ylidene)_2_ moiety to rotate relative to the anthracene group appears to be significant for the outcome of the reaction of the respective tetracarbene ligands with silver and gold ions. To further corroborate this conclusion, the tetrakisimidazolium salt H_4_‐**1d**(PF_6_)_4_ was prepared (Scheme 1, Figures S36–S41). Ligand precursor H_4_‐**1d**(PF_6_)_4_ features an N(phenylimidazolium)_2_ group directly bound to anthracene and two of the substituted anthracenes are then connected by a phenyl bridge. This leads to a ligand precursor more flexible than the related tetra‐NHC precursor H_4_‐**1a**(PF_6_)_4_. However, due to the direct link of the N(phenylimidazolium)_2_ groups to anthracene, restriction of rotation about the N─C_anthracene_ bond can be expected.

Compound H_4_‐**1d**(PF_6_)_4_⋅4CH_3_CN was characterized by an X‐ray diffraction study revealing that tetracation H_4_‐**1d** ^4+^ resides on a crystallographic inversion center (Figure S53). The angle between the C_phenyl_─N─C_phenyl_ plane of the N(phenylimidazolium)_2_ group and the anthracene plane was determined as 69.64°. This value is rather similar to the equivalent angles found in ligand precursors H_4_‐**1a**(PF_6_)_4_ (72.26°) and H_4_‐**1c**(PF_6_)_4_ (72.72°), indicative of a restricted rotation about the N─C_anthracene_ bond.

The reaction of H_4_‐**1d**(PF_6_)_4_ with an excess of Ag_2_O in acetonitrile yielded the octanuclear assembly [Ag_8_(**1d**)_4_](PF_6_)_8_. Although the ^1^H NMR spectrum of the assembly was not informative (Figure S42), the HR‐ESI mass spectrum clearly showed the formation of the octacation [Ag_8_(**1d**)_4_]^8+^ with the strongest peak at *m*/*z* = 678.5414 (calcd for [Ag_8_(**1d**)_4_]^8+^ 678.5470, Figure S43). In order to enhance the stability of the assembly and to facilitate its crystallization, ligand transmetalation using [AuCl(THT)] was carried out to give with retention of the octanuclear structure the assembly [Au_8_(**1d**)_4_](PF_6_)_8_ verified by ^1^H NMR spectroscopy (Figure S44) and HR‐ESI mass spectrometry (Figure S45) showing the strongest peak at *m*/*z* = 767.5905 (calcd for [Au_8_(**1d**)_4_]^8+^ 767.6084).

**Figure 6:**
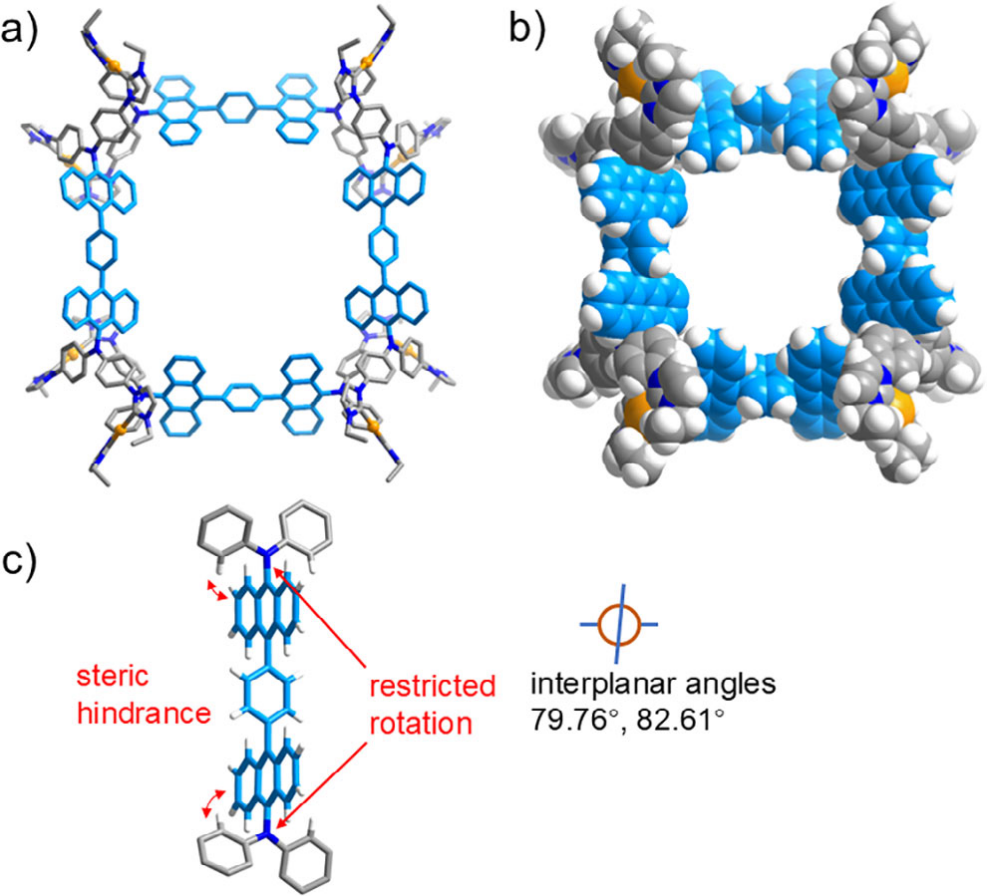
a) Molecular structure of the cation [Au_8_(**1d**)_4_]^8+^ in [Au_8_(**1d**)_4_](PF_6_)_8_. b) Space filling representation of cation [Au_8_(**1d**)_4_]^8+^. c) Representation of one of the four Ph_2_N─anthracene─NPh_2_ units showing the restricted rotation about anthracene─NPh_2_ bonds.

An X‐ray diffraction study with crystals of composition [Au_8_(**1d**)_4_](PF_6_)_8_ confirmed the formation of the octacation [Au_8_(**1d**)_4_]^8+^ (Figure 6a,b), bisected by two mirror planes. The restriction of rotation about the N─C_anthracene_ bond (Figure 6c) enforced the formation of the octanuclear assembly, similarly to the structure of octacation [Au_8_(**1a**)_4_]^8+^ (Figure 3a).

The angles between the C_phenyl_─N─C_phenyl_ planes of the N(phenylimidazol‐2‐ylidene)_2_ groups and the anthracene they are attached to were determined as 79.76° and 82.61°. These values are rather similar to the equivalent interplanar angles observed for [Au_8_(**1a**)_4_]^8+^ (range 76.72° to 78.70°).

## Conclusion

In summary, we have successfully applied the directional bonding strategy to the coordination‐driven self‐assembly of polynuclear polycarbene structures. By modulating the degree of rotation of the C_phenyl_─N─C_phenyl_ plane of di(phenylimidazol‐2‐ylidene)amine groups relative to the plane of a bridging anthracene group in a series of tetracarbene ligands, discrete assemblies of types M_4_L_2_‐, M_6_L_3_‐, and M_8_L_4_ have been obtained. Only the cylinder‐like M_4_L_2_ structure, obtained from tetra‐NHCs featuring free rotation of the NHC relative to the central linker (phenyl^[^ ^65^, ^66^ ^]^ or tetraphenylethylene^[^ ^75^ ^]^) has been known previously. Hindered rotation has been demonstrated in both the tetracarbene precursor salts and in the polynuclear assemblies obtained from these. Although the results at this time are descriptive in nature, they can form the basis for further investigations on directional bonding in the coordination‐driven self‐assembly with polycarbene ligands.

## Conflict of Interests

The authors declare no conflict of interest.

## Supporting information



Supporting Information

Supporting Information

## Data Availability

The data that support the findings of this study are available in the Supporting Information of this article.
