# Supplementary material for: The Flexibility of Tetra(N‐Heterocyclic Carbene) Ligands Controls the Nuclearity and Geometry of Polynuclear MI‒NHC Assemblies
Source: Angew Chem Int Ed Engl. 2025 May 19;64(25):e202502081. doi: 10.1002/anie.202502081 (PMC12171663; doi:10.1002/anie.202502081)
Supplement: Supplementary file 1 — Supporting Information [file ANIE-64-e202502081-s001.pdf]

Table of Contents

1. Materials and methods S2

2. Synthesis of ligand precursor H_4-**1a**$(PF_6)_4$ and of $[Ag_8($**1a**$)_4](PF_6)_8$ and $[Au_8($**1a**$)_4](PF_6)_8$ S3

3. Synthesis of ligand precursor H_4-**1b**$(PF_6)_4$ and of $[Ag_4($**1b**$)_2](PF_6)_4$ and $[Au_4($**1b**$)_2](PF_6)_4$ S7

4. Synthesis of ligand precursor H_4-**1c**$(PF_6)_4$ and of $[Ag_6($**1c**$)_3](PF_6)_6$ and $[Au_6($**1c**$)_3](PF_6)_6$ S11

5. Synthesis of ligand precursor H_4-**1d**$(PF_6)_4$ and of $[Ag_8($**1d**$)_4](PF_6)_8$ and $[Au_8($**1d**$)_4](PF_6)_8$ S14

6. Selected NMR and MS spectra for new compounds S18

7. X-ray crystallography S43

8. Density Functional Theory Calculations S59

9. References S59

1. Materials and methods

All starting materials were used as received from commercial sources. Solvents (acetonitrile, THF and toluene) were freshly distilled by standard procedures prior to use. The experiments were carried out under a nitrogen atmosphere with standard Schlenk techniques or under an argon atmosphere in the glove box. The ^{1}H, $^{13}C\{^{1}H\}$, and 2D nuclear magnetic response (NMR) spectra were recorded on Bruker AVANCE III 400 ord JEOL ECZ400R spectrometers. Chemical shifts (δ) are expressed in ppm downfield from tetramethylsilane using the residual protonated solvent as an internal standard. Mass spectra were obtained with a Bruker microTOF-Q II mass spectrometer (Bruker Daltonics Corp., USA) in the electrospray ionization (ESI) mode. The fluorescence experiments were performed on a QuantaMaster 8000 spectrometer (HORIBA Scientific, Canada; slit width 2 nm). A complete set of NMR spectra (^{1}H and $^{13}C\{^{1}H\}$) and electrospray ionization-mass spectrometry (ESI-MS) spectra are provided. Compound 10-bromo-*N*,*N*-diphenylanthracen-9-amine was synthesized following a published procedure.[S1]

2. Synthesis of ligand precursor H_4-1a(PF_6)$_4$ and of [Ag_8(1a)$_4$](PF_6)$_8$ and [Au_8(1a)$_4$](PF_6)$_8$

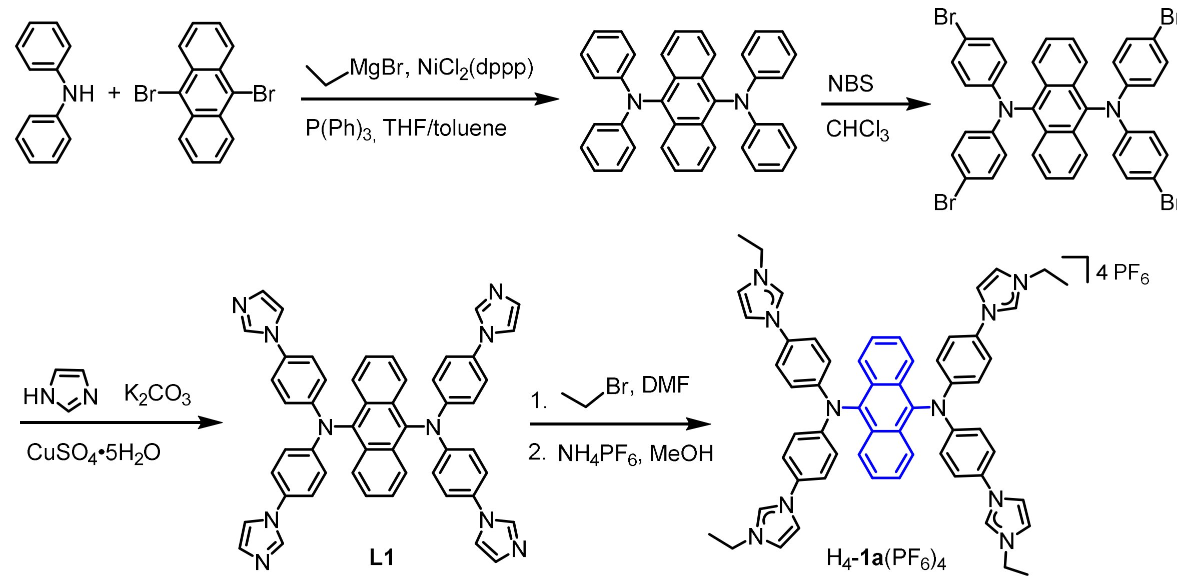

Scheme S1. Synthesis of the tetra-NHC precursor H_4-**1a**(PF_6)$_4$.

Synthesis of *N,N,N',N'*-tetrakis(phenyl)-*p*-(9,10-anthracene)diamine

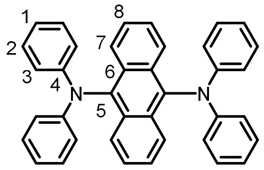

Diphenylamine (0.500 g, 2.955 mmol) was dissolved in THF (4 mL) at ambient temperature in a glove box. Ethyl magnesium bromide (3.2 mL of an 1*M* solution in THF, 3.2 mmol) was added dropwise over 10 min and the mixture was stirred for 5 minutes. Subsequently, $NiCl_2$(dppp) (0.347 g, 0.064 mmol), triphenylphosphine (0.347 g, 0.132 mmol) and 9,10-dibromoanthracene (0.427 g, 1.271 mmol) in dry toluene (10 mL) were added dropwise. The mixture was stirred under reflux for 36 h. It was then poured into a solution of concentrated HCl (4 mL) in water (20 mL). The mixture was stirred at ambient temperature for 1 h. Subsequently, the solution was neutralized with Na_2CO_3, followed by extraction with dichloromethane. The organic phase was washed with water and dried over anhydrous Na_2SO_4. The solvent was removed under reduced pressure to give an orange solid. Yield: 0.300 g (0.585 mmol, 46%). ^{1}H NMR (400 MHz, $CDCl_3$): δ= 8.20 (m, 4H, H8), 7.36 (m, 4H, H7), 7.22 (m, 8H, H2), 7.12 (m, 8H, H3), 6.92 (m, 4H, H1). ^{13}C{^{1}H} NMR (100 MHz, $CDCl_3$): δ= 147.7, 137.4, 131.8, 129.2, 126.7, 125.0, 121.3, 120.3 (Ar-C).

Synthesis of *N,N,N',N'*-tetrakis(4-bromophenyl)-*p*-(9,10-anthracene)diamine

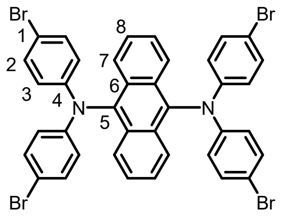

To a solution of *N,N,N',N'*-tetraphenyl-*p*-(9,10-anthracene)diamine (0.3 g, 0.585 mmol) in 70 mL of CH_3Cl, *N*-bromosuccinimide (0.460 g, 2.585 mmol) in 0.5 mL of DMF was added slowly dropwise. The reaction mixture was stirred for 5 h at ambient temperature. The formed solid was isolated by filtration and dried under reduced pressure to give a yellow solid. Yield: 0.350 g (0.423 mmol, 72%). ^{1}H NMR (400 MHz, $CDCl_3$): δ = 8.08 (m, 4H, H8), 7.41 (m, 4H, H7), 7.30 (d, J = 8.0 Hz, 8H, H2), 6.95 (d, J = 8.0 Hz, 8H, H3). $^{13}C\{^1H\}$ NMR (100 MHz, $CDCl_3$): δ = 146.3, 135.4, 132. 4, 131.2, 127.3, 124.6, 121.8, 118.9 (Ar-C).

Synthesis of ligand precursor H_4-1a(PF_6)$_4$

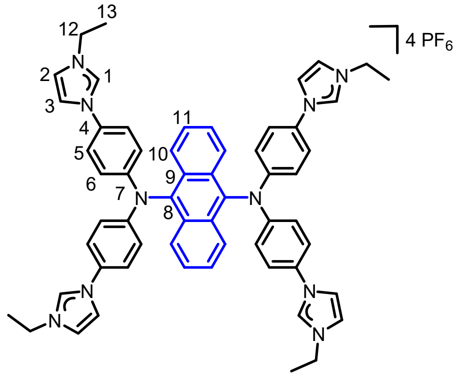

Samples of *N,N,N',N'*-tetrakis(4-bromophenyl)-*p*-(9,10-anthracene)diamine (0.200 g, 0.241 mmol), imidazole (1.314 g, 19.300 mmol), K_2CO_3 (1.333 g, 9.645 mmol), $CuSO_4·5H_2O$ (0.050 g, 0.200 mmol) were mixed and the mixture was heated to 200 °C for 60 h. The mixture was then cooled to ambient temperature and washed several times with water. The remaining solid residue was brought to dryness to give **L1** as pale-green solid. Yield: 0.160 g (0.206 mmol, 85%). **L1** (0.160 g, 0.206 mmol) and an excess of ethyl bromide (1.2 mL, 1.752 g, 1.608 mmol) in DMF (2 mL) were heated to 130 °C for 24 h. After cooling to ambient temperature, ethyl acetate (20 mL) was added leading to the precipitation of a grey solid. The solid was isolated by filtration and dried *in vacuo*. To the solid were added methanol (30 mL) and NH_4PF_6 (0.325 g, 1.994 mmol). The mixture was stirred for 24 h at ambient temperature. Over this period, the hexafluorophosphate salt H_4-**1a**(PF_6)$_4$ precipitated. The solid was isolated by filtration, washed with methanol and dried *in vacuo*. Yield: 0.290 g (0.197 mmol, 95%). ^{1}H NMR (400 MHz, CD_3CN): δ = 8.83 (s, 4H, H1), 8.21 (m, 4H, H11), 7.68 (s, 4H, H3), 7.56 (s, 4H, H2),7.54 (d, J = 4.0 Hz, 4H, H10), 7.49 (d, J = 8.0 Hz, 8H, H6), 7.38 (d, J = 8.0 Hz, 8H, H5), 4.26 (q, J = 7.2 Hz, 8H, H12), 1.52 (t, J = 7.2 Hz, 12H, H13). $^{13}C\{^1H\}$ NMR (100 MHz, CD_3CN): δ = 149.1 (C7), 137.7 (C8), 135.0 (C1), 132.3 (C9), 130.0 (C4), 129.0 (C10), 125.3 (C11), 124.7 (C6), 123.7 (C2), 122.7 (C3), 122.4 (C5), 46.3 (C12), 15.2 (C13). ESI-MS (positive ions): m/z = 223.1433 (calcd for [H_4-**1a**]$^{4+}$ 223.1172), 345.8233 (calcd for [H_4-

1a(PF_6)]$^{3+}$ 345.8110).

Synthesis of [Ag_8(1a)$_4$](PF_6)$_8$

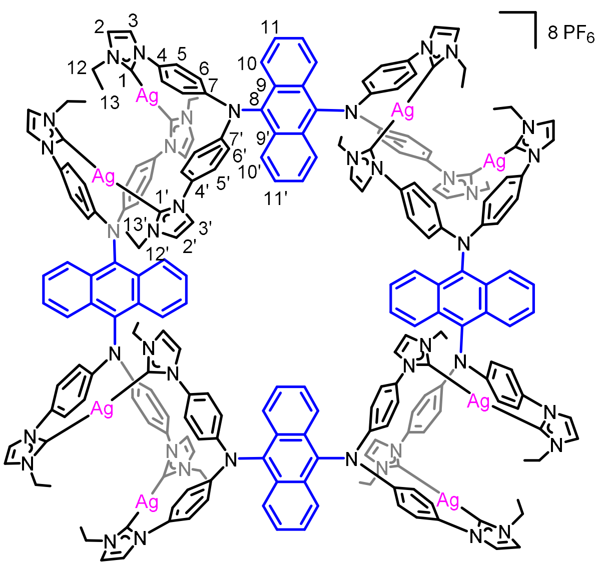

A sample of H_4-**1a**(PF_6)$_4$ (0.048 g, 0.033 mmol) was treated with Ag_2O (0.036 g, 0.156 mmol) in dry acetonitrile (10 mL). The reaction mixture was then heated to 70 °C for 24 h under exclusion of light. After cooling of the reaction mixture to ambient temperature, the resulting suspension was centrifuged and the green solution was separated by filtration. The filtrate was concentrated to 2 mL and diethyl ether (20 mL) was added. This led to precipitation of complex [Ag_8(**1a**)$_4$](PF_6)$_8$ as a gray solid. The solid was collected by filtration, washed with diethyl ether, and dried *in vacuo*. Yield: 0.041 g (0.007 mmol, 85%). ^{1}H NMR (400 MHz, CD_3CN): δ = 8.26 (m, 8H, H11), 8.10 (m, 8H, H11'), 7.81 (d, J = 8.0 Hz, 16H, H6'), 7.61 (d, J = 4.0 Hz, 8H, H10'), 7.57 (s, 8H, H3'), 7.45 (s, 8H, H2'), 7.34 (s, 8H, H2), 7.22 (d, J = 12.0 Hz, 16H, H5), 7.21 (d, J = 12.0 Hz, 16H, H6), 7.16 (s, 8H, H3), 7.11 (d, J = 8.0 Hz, 16H, H5'), 7.05 (d, J = 4.0 Hz, 8H, H10), 4.31–4.15 (m, 32H, H12', H12), 1.52–1.46 (m, 48H, H13', H13). ESI-MS (positive ions): m/z = 552.3891 (calcd for [Ag_8(**1a**)$_4$]$^{8+}$ 552.3747), 652.0094 (calcd for [Ag_8(**1a**)$_4$(PF_6)]$^{7+}$ 651.9946), 784.8342 (calcd for [Ag_8(**1a**)$_4$(PF_6)$_2$]$^{6+}$ 784.8210), 970.7913 (calcd for [Ag_8(**1a**)$_4$(PF_6)$_3$]$^{5+}$ 970.7780).

Synthesis of [Au_8(1a)$_4$](PF_6)$_8$

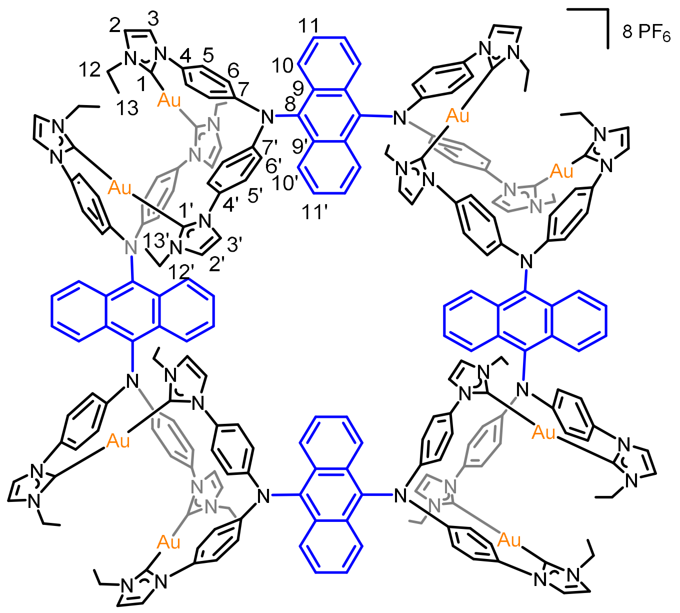

A sample of [Ag_8(**1a**)$_4$](PF_6)$_8$ (0.041 g, 0.007 mmol) was treated [AuCl(THT)] (0.028 g, 0.087 mmol) in acetonitrile (10 mL). The reaction mixture was stirred at ambient temperature for 24 h. The resulting suspension was centrifuged and the reddish-brown solution was separated by filtration. The filtrate was concentrated to 2 mL and diethyl ether (20 mL) was added. This led to the precipitation of [Au_8(**1a**)$_4$](PF_6)$_8$ as a gray solid. The solid was collected by filtration, washed with diethyl ether and dried *in vacuo*. Yield: 0.036 g (0.006 mmol, 86%). ^{1}H NMR (400 MHz, CD_3CN): δ = 8.28 (m, 8H, H11), 8.13 (m, 8H, H11'), 7.86 (d, J = 8.0 Hz, 16H, H6'), 7.61 (d, J = 4.0 Hz, 8H, H10'), 7.55 (s, 8H, H3'), 7.46 (s, 8H, H2'), 7.34 (s, 8H, H2), 7.29 (d, J = 12.0 Hz, 16H, H5), 7.21 (d, J = 12.0 Hz, 16H, H6), 7.16 (s, 8H, H3), 7.10 (d, 16H, J = 16 Hz, H5'), 7.05 (d, J = 4.0 Hz, 8H, H10), 4.35–4.19 (m, 32H, H12', H12), 1.54–1.47 (m, 48H, H13', H13). ^{13}C{^{1}H} NMR (100 MHz, CD_3CN): δ = 183.3 (C1), 182.5 (C1'), 147.6 (C7'), 145.7 (C7), 137.7 (C8), 135.1 (C4), 133.8 (C4'), 132.9 (C9'), 132.1 (C9), 129.7 (C10'), 129.0 (C10), 127.4 (C6'), 126.2 (C5), 125.8 (C11'), 124.4 (C11), 123.8 (C6), 123.4 (C3'), 123.0 (C2), 122.9 (C2'), 122.6 (C3), 117.2 (C5'), 47.7 (C12', C12), 17.4 (C13'), 17.2 (C13). ESI-MS (positive ions): m/z = 641.4421 (calcd for [Au_8(**1a**)$_4$]$^{8+}$ 641.4361), 753.7860 (calcd for [Au_8(**1a**)$_4$(PF_6)]$^{7+}$ 753.7790), 903.5777 (calcd for [Au_8(**1a**)$_4$(PF_6)$_2$]$^{6+}$ 903.5695).

3. Synthesis of ligand precursor H_4-1b$(PF_6)_4$ and of $[Ag_4(1b)_2](PF_6)_4$ and $[Au_4(1b)_2](PF_6)_4$

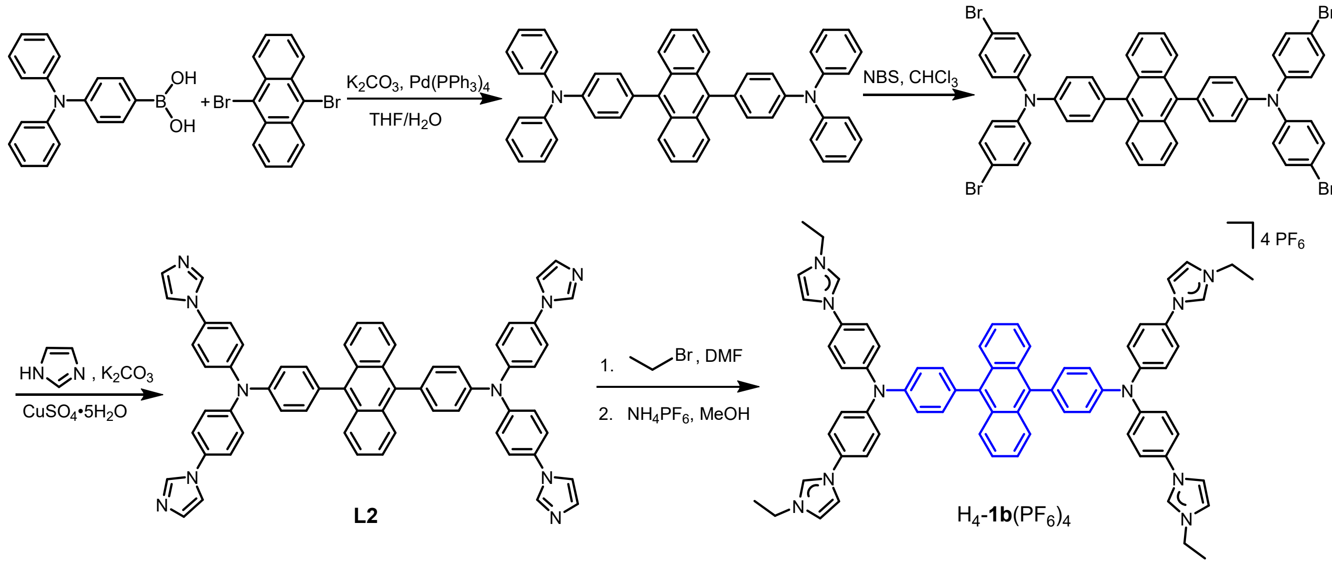

Scheme S2. Synthesis of the tetra-NHC precursor H_4-**1b**$(PF_6)_4$.

Synthesis of *N,N,N',N'*-tetrakis(phenyl)-*p*-(9,10-diphenylanthracene)diamine

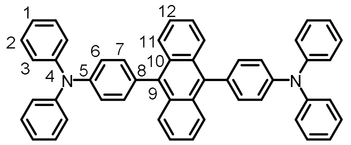

A 50 mL Schlenk flask was charged with the 4-(diphenylamino)phenylboronic acid (0.600 g, 2.08 mmol), 9,10-dibromoanthracene (0.316 g, 0.940 mmol), THF (20 mL), 10 mL of aqueous 1 *M* K_2CO_3 (1.382 g, 10.0 mmol) and $Pd(PPh_3)_4$ (0.080 g, 0.07 mmol). The mixture was heated under reflux for 24 h. After cooling, the mixture was filtered and the solid residue was washed with THF. Drying *in vacuo* afforded the target compound as a yellow solid. Yield: 0.284 g (0.427 mmol, 71%). ^1H NMR (400 MHz, $CDCl_3$): δ = 7.85 (m, 4H, H12), 7.41 (d, *J* = 4.0 Hz, 4H, H11), 7.34 (m, 12H, H2 and H6), 7.31 (m, 12H, H3 and H7), 7.11 (m, 4H, H1). $^{13}C\{^1H\}$ NMR (100 MHz, $CDCl_3$): δ = 147.9, 147.2, 136.9, 132.8, 132.3, 130.3, 129.5, 127.2, 125.1, 124.8, 123.3, 123.2 (Ar-C).

Synthesis of *N,N,N',N'*-tetrakis(4-bromophenyl)-*p*-(9,10-diphenylanthracene)diamine

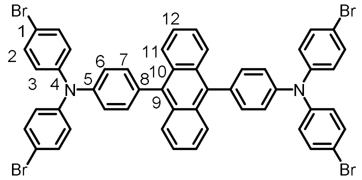

To a solution of *N,N,N',N'*-tetraphenyl-*p*-(9,10-diphenylanthracene)-diamine (0.400 g, 0.602 mmol) in 20 mL of CH_2Cl_2 was added dropwise *N*-bromosuccinimide (0.480 g, 2.70 mmol) in 0.5 mL of DMF. The reaction mixture was stirred for 5 h at the ambient temperature. The solid residue was isolated by filtration and dried to give a pale solid. Yield: 0.440 g (0.449 mmol, 75%). ^1H NMR (400

MHz, $CDCl_3$): δ = 7.80 (m, 4H, H12), 7.44 (d, J = 8.0 Hz, 8H, H2), 7.40 (d, J = 4.0 Hz, 4H, H11), 7.35 (d, J = 8.0 Hz, 4H, H6), 7.28 (d, J = 8.0 Hz, 4H, H7), 7.13 (m, 8H, H3). The solubility of the compound in common organic solvents was rather poor. As a result, the $^{13}C\{^1H\}$ NMR spectrum was not recorded.

Synthesis of ligand precursor H_4-1b(PF_6)$_4$

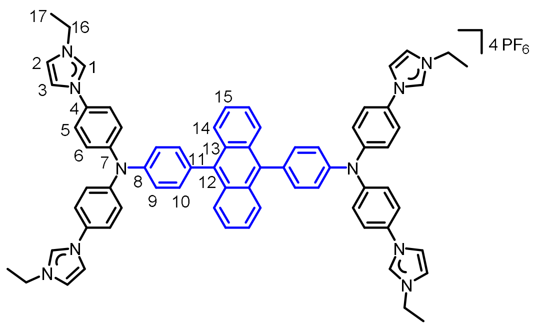

Samples of *N,N,N',N'*-tetrakis(4-bromophenyl)-*p*-(9,10-diphenylanthracene)diamine (0.401 g, 0.409 mmol), imidazole (8.0 g, 117.5 mmol), K_2CO_3 (8.0 g, 57.9 mmol), $CuSO_4\cdot5H_2O$ (0.500 g, 2.002 mmol) were mixed and the mixture was heated to 180 °C for 48 h. The reaction mixture was then cooled to ambient temperature and washed several times with water. The remaining solid residue was brought to dryness to give **L2** as a pale green solid. Yield: 0.363 g (0.391 mmol, 96%). A sample of **L2** (0.310 g, 0.334 mmol) and an excess of ethyl bromide (5.0 mL, 7.3 g, 6.70 mmol) were put in a 50 mL Schlenk flask together with 10 mL of DMF. The mixture was heated to 130 °C for 24 h. After cooling to ambient temperature, the solvents were evaporated to obtain a gray powder. The powder obtained was mixed with NH_4PF_6 (1.0 g, 6.13 mmol) in 30 mL of methanol. The mixture was stirred for 24 h at ambient temperature. Over this period, the hexafluorophosphate salt H_4-**1b**(PF_6)$_4$ precipitated. The precipitate was collected by filtration, washed with methanol and dried *in vacuo*. Yield: 0.409 g (0.252 mmol, 75%). ^1H NMR (400 MHz, CD_3CN): δ = 9.04 (s, 4H, H1), 7.84 (m, 4H, H15), 7.78–7.44 (m, 36H, H2, H3, H5, H6, H9, H10, H14), 4.31 (q, J = 8.0 Hz, 8H, H16), 1.57 (t, J = 8.0 Hz, 12H, H17). $^{13}C\{^1H\}$ NMR (100 MHz, CD_3CN): δ = 149.7 (C7), 146.8 (C8), 135.1 (C1), 137.5, 136.7, 133.8, 130.9, 130.8, 127.7, 127.1, 126.6, 125.5 (C4, C5, C9, C10, C11, C12, C13, C14, C15), 124.8 (C6), 123.8 (C2), 122.9 (C3), 45.1 (C16), 14.0 (C17). ESI-MS (positive ions): m/z = 627.2205 (calcd for [H_4-**1b**(PF_6)$_2$]$^{2+}$ 627.2299).

Synthesis of [Ag_4(**1b**)$_2$](PF_6)$_4$

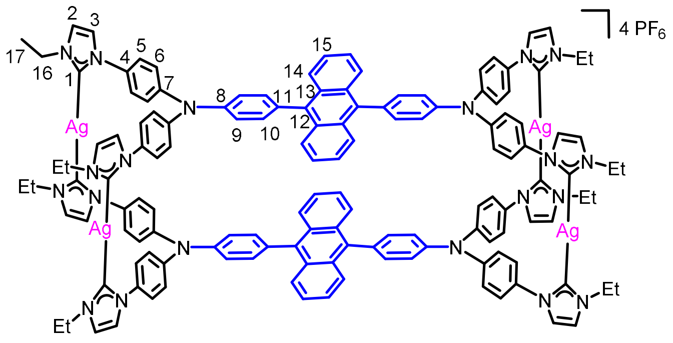

To a mixture of H_4-**1b**(PF_6)$_4$ (0.040 g, 0.025 mmol) and Ag_2O (0.032 g, 0.140 mmol) was added 15 mL dry acetonitrile. The reaction mixture was then heated to 70 °C for 24 h under exclusion of light. After cooling of the reaction mixture to ambient temperature, the resulting suspension was centrifuged to give a green solution which was separated by filtration. The filtrate was concentrated to 2 mL and diethyl ether (20 mL) was added leading to precipitation of [Ag_4(**1b**)$_2$](PF_6)$_4$ as gray solid. The solid was collected by filtration, washed with diethyl ether, and dried *in vacuo*. Yield: 0.031 g (0.010 mmol, 80%). ^{1}H NMR (400 MHz, CD_3CN): δ = 7.58–7.55 (m, 24H, H6 and H14), 7.50 (d, J = 4.0 Hz, 8H, H2), 7.48 (d, J = 4.0 Hz, 8H, H3), 7.36 (d, J = 8.0 Hz, 16H, H5), 7.29 (d, J = 4.0 Hz, 8H, H10), 7.19 (d, J = 4.0 Hz, 8H, H9), 6.95 (m, 8H, H15), 4.40 (q, J = 8.0 Hz, 16H, H16), 1.60 (t, J = 8.0 Hz, 24H, H17). ^{13}C{^{1}H} NMR (100 MHz, CD_3CN): δ = 176.0 (C1), 148.9 (C4), 147.6 (C11), 136.4 (C13), 135.2 (C7), 133.7 (C8), 130.8 (C9), 130.7 (C12), 130.2 (C14), 127.3 (C6), 126.3 (C5), 125.7 (C10), 125.4 (C15), 123.3 (C3), 123.1 (C2), 48.5 (C16), 17.7 (C17). ESI-MS (positive ions): *m/z* = 628.4010 (calcd for [Ag_4(**1b**)$_2$]$^{4+}$ 628.4062), 886.1859 (calcd for [Ag_4(**1b**)$_2$(PF_6)]$^{3+}$ 886.1964).

Synthesis of [Au_4(**1b**)$_2$](PF_6)$_4$

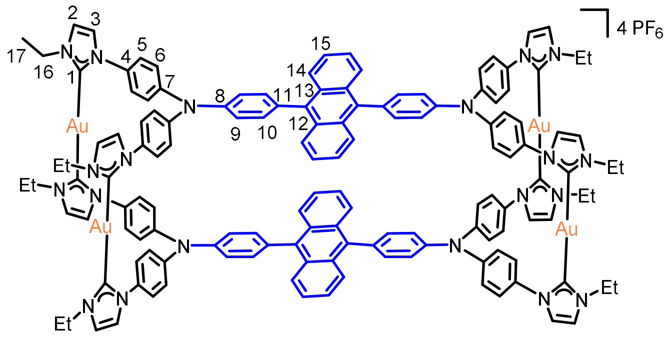

Solid [AuCl(THT)] (0.015 g, 0.047 mmol) was added to a solution of [Ag_4(**1b**)$_2$](PF_6)$_4$ (0.033 g, 0.011 mmol) in dry acetonitrile (10 mL). The reaction mixture was stirred at ambient temperature for 24 h. The resulting suspension was centrifuged to give a reddish-brown solution which was isolated by filtration. The filtrate was concentrated to 2 mL and diethyl ether (20 mL) was added resulting in the precipitation of a gray solid. The precipitate was collected by filtration, washed with diethyl ether and dried *in vacuo*. Yield: 0.032 g (0.009 mmol, 90%). ^{1}H NMR (400 MHz, CD_3CN): δ = 7.58 (m, 24H, H6 and H14), 7.48 (d, J = 2.0 Hz, 8H, H2), 7.46 (d, J

= 2.0 Hz, 8H, H3), 7.36 (d, J = 8.0 Hz, 16H, H5), 7.30 (d, J = 8.4 Hz, 8H, H10), 7.20 (d, J = 8.4 Hz, 8H, H9), 6.96 (m, 8H, H15), 4.48 (q, J = 7.2 Hz, 16H, H16), 1.62 (t, J = 7.2 Hz, 24H, H17). $^{13}C\{^1H\}$ NMR (100 MHz, CD_3CN): δ = 182.3 (C1), 148.9 (C4), 147.2 (C11), 136.4 (C13), 135.4 (C7), 134.8 (C8), 133.6 (C9), 130.1 (C12), 127.3 (C14), 126.3 (C6), 126.0 (C5), 125.4 (C10, C15), 123.8 (C3), 123.3 (C2), 47.8 (C16), 17.4 (C17). ESI-MS (positive ions): *m/z* = 717.4595 (calcd for $[Au_4(\mathbf{1b})_2]^{4+}$ 717.4669).

4. Synthesis of ligand precursor H_4-1c(PF_6)$_4$ and of [Ag_6(1c)$_3$](PF_6)$_6$ and [Au_6(1c)$_3$](PF_6)$_6$

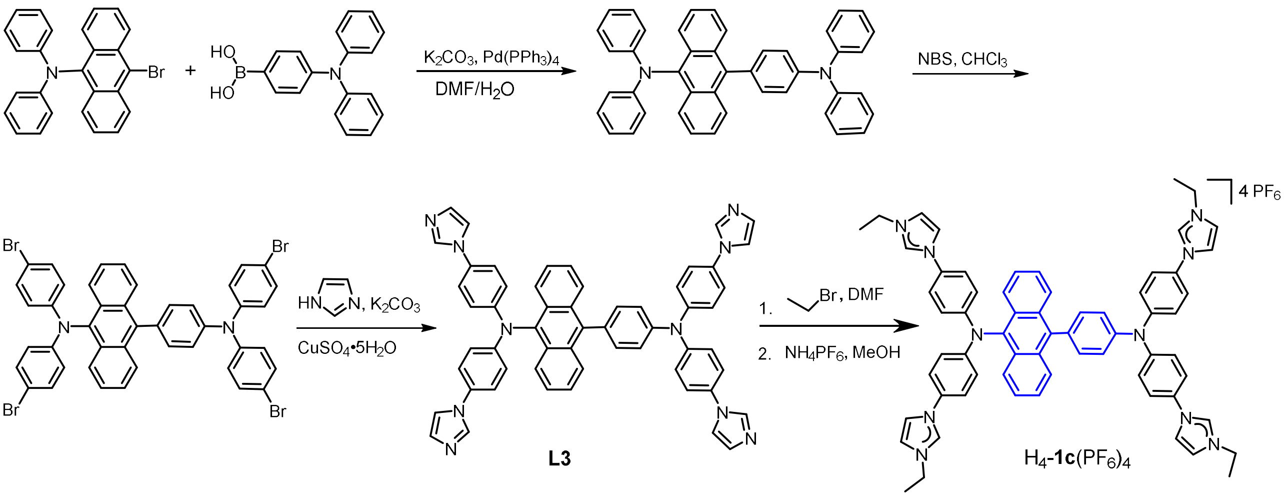

Scheme S3. Synthesis of the tetra-NHC precursor H_4-**1c**(PF_6)$_4$.

Synthesis of 10-(4-(diphenylamino)phenyl)-*N*,*N*-diphenylanthracen-9-amine

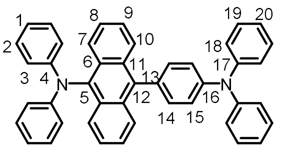

A 50 mL Schlenk flask was charged with 4-(diphenylamino)phenylboronic acid (0.150 g, 0.52 mmol), 10-bromo-*N*,*N*-diphenylanthracen-9-amine (0.200 g, 0.47 mmol), DMF (20 mL), 10 mL of 1 *M* aqueous K_2CO_3 (1.382 g, 10.00 mmol) and Pd(PPh_3)$_4$ (0.040 g, 0.035 mmol). The mixture was heated to 100 °C for 24 h. After cooling to ambient temperature, the mixture was extracted with CH_2Cl_2. The organic phase was washed with water and dried over anhydrous Na_2SO_4 before the solvent was evaporated to give a yellow solid. Yield: 0.252 g (0.428 mmol, 91%). ^{1}H NMR (400 MHz, $CDCl_3$): δ= 8.19–8.17 (m, 2H), 7.87–7.85 (m, 2H), 7.40–7.32 (m, 10H), 7.30–7.28 (m, 6H), 7.19–7.07 (m, 10H), 6.90–6.86 (m, 2H). The solubility of the product was rather poor in common organic solvents. As a result, the ^{13}C{^{1}H} NMR spectrum was not recorded.

Synthesis of 10-(4-(bis(4-bromophenyl)amino)phenyl)-*N*,*N*-bis(4-bromophenyl)anthracene-9-amine

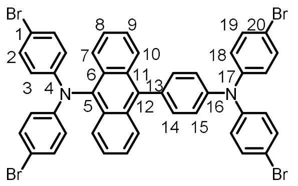

To a solution of 10-(4-(diphenylamino)phenyl)-*N*,*N*-diphenylanthracen-9-amine (0.200 g, 0.340 mmol) in 20 mL of $CHCl_3$ was added dropwise *N*-bromosuccinimide (0.251 g, 1.410 mmol) in 0.5 mL of DMF. The reaction mixture was stirred for 15 h at the ambient temperature. The solvent was evaporated and the remaining solid was washed with water (10 mL). Column chromatography of the

solid on SiO_2 (eluents petroleum ether (60–80 °C) and CH_2Cl_2, 10:1, v:v) and evaporation of the solvent gave a yellow solid. Yield: 0.280 g (0.309 mmol, 91%). ^{1}H NMR (400 MHz, $CDCl_3$): δ = 8.08–8.06 (m, 2H), 7.83–7.81 (m, 2H), 7.64–7.34 (m, 8H), 7.28 (s, 8H), 7.14–7.11 (m, 4H), 6.98–6.96 (m, 4H). ^{13}C{^{1}H} NMR (100 MHz, $CDCl_3$): δ = 146.6, 146.5, 137.9, 135.9, 133.0, 132.7, 132.5, 132.5, 132.4, 131.5, 130.1, 127.8, 127.1, 126.2, 125.7, 123.9, 123.5, 121.9, 116.2, 114.0 (Ar-C).

Synthesis of ligand precursor H_4-1c(PF_6)$_4$

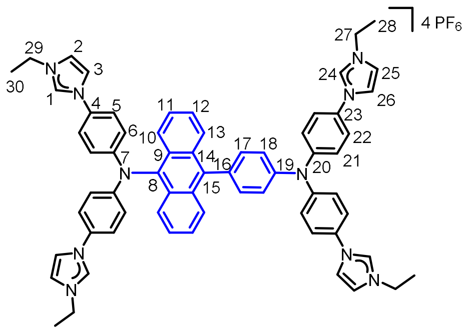

A sample of 10-(4-(bis(4-bromophenyl)amino)phenyl)-*N*,*N*-bis(4-bromophenyl)anthracene-9-amine (0.431 g, 0.477 mmol), imidazole (12.0 g, 176.2 mmol), K_2CO_3 (12.0 g, 86.8 mmol), $CuSO_4\cdot 5H_2O$ (0.800 g, 3.20 mmol) were mixed and the mixture was heated to 190 °C for 48 h. The reaction mixture was then cooled to ambient temperature and washed five times with water (40 mL each). The remaining solid residue was brought to dryness to give **L3** as a pale-green solid. Yield: 0.395 g (0.463 mmol, 97%). A sample of **L3** (0.210 g, 0.246 mmol) and an excess of ethyl bromide (5.0 mL, 7.30 g, 6.70 mmol) were mixed in a 50 mL Schlenk flask with 10 mL of DMF. The mixture was heated to 110 °C for 48 h. After cooling to ambient temperature and evaporation of the solvent, methanol (20 mL) and NH_4PF_6 (0.600 g, 3.68 mmol) were added. The mixture was stirred for 24 h at ambient temperature. Over this period, the hexafluorophosphate salt H_4-**1c**(PF_6)$_4$ precipitated. The solid was collected by filtration, washed with methanol and dried *in vacuo*. Yield: 0.205 g (0.132 mmol, 54%). ^{1}H NMR (400 MHz, CD_3CN): δ = 8.96 (s, 2H, H1), 8.85 (s, 2H, H24), 8.16 (d, J = 8.0 Hz, 2H, H10), 7.91 (d, J = 8.0 Hz, 2H, H13), 7.77 (s, 2H, H3), 7.68 (s, 2H, H26), 7.62–7.47 (m, 24H, H2, H5, H6, H11, H12, H17, H18, H21/22, H25), 7.34 (4H, H21/22), 4.33–4.22 (m, 8H, H29, H27), 1.59–1.50 (m, 12H, H30, H28). ^{13}C{^{1}H} NMR (100 MHz, CD_3CN): δ = 149.6 (C20), 149.1 (C7), 147.0 (C16/19), 139.5 (C15), 136.2 (C8), 135.9 (C5/6), 135.1 (C1), 134.9 (C24), 133.6 (C17/18), 132.4 (C11), 130.7 (C12), 130.7 (C4), 129.7 (C23), 128.7 (C13), 128.6 (C14), 127.0 (C16/19), 126.9 (C9), 125.5 (C5/6), 124.8 (C21/22), 124.6 (C17/18), 124.1 (C10), 123.8 (C2), 123.7 (C25), 122.8 (C3), 122.7 (C26), 122.1 (C21/22), 46.3 (C29, C27), 15.2 (C30, C28). ESI-MS (positive ions): m/z = 242.1320 (calcd for $[H_4\text{-}\mathbf{1c}]^{4+}$ 242.1250).

Synthesis of $[Ag_6(\mathbf{1c})_3](PF_6)_6$

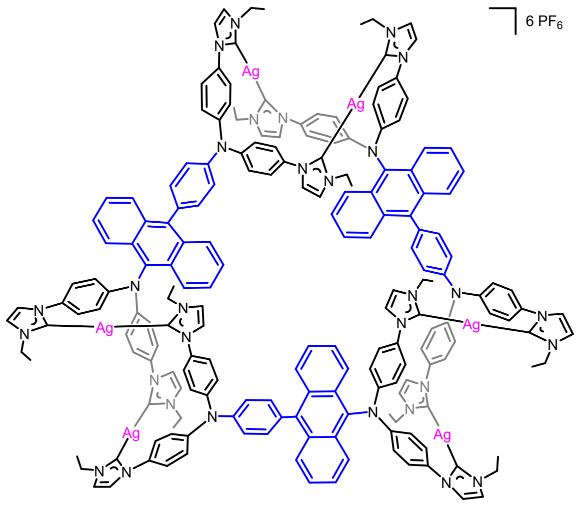

To a mixture of H_4-**1c**$(PF_6)_4$ (0.083 g, 0.054 mmol) and Ag_2O (0.051 g, 0.220 mmol) was added 15 mL of dry acetonitrile. The reaction mixture was heated to 80 ºC for 34 h under exclusion of light. After cooling of the reaction mixture to ambient temperature, the resulting suspension was centrifuged and the green solution was isolated by filtration. The filtrate was concentrated to 1 mL and diethyl ether (20 mL) was added leading to precipitation of $[Ag_6(\mathbf{1c})_3](PF_6)_6$ as a gray solid. The solid was collected by filtration, washed with diethyl ether and dried *in vacuo*. Yield: 0.073 g (0.0165 mmol, 92%). ^{1}H NMR (400 MHz, CD_3CN): δ = 7.63–7.29 (m, 96H), 7.05 (m, 12H), 4.31–4.28 (m, 24H), 1.55–1.50 (m, 36H). ESI-MS (positive ions): *m/z* = 590.4851 (calcd for $[Ag_6(\mathbf{1c})_3]^{6+}$ 590.4738), 737.5645 (calcd for $[Ag_6(\mathbf{1c})_3(PF_6)]^{5+}$ 737.5614).

Synthesis of $[Au_6(\mathbf{1c})_3](PF_6)_6$

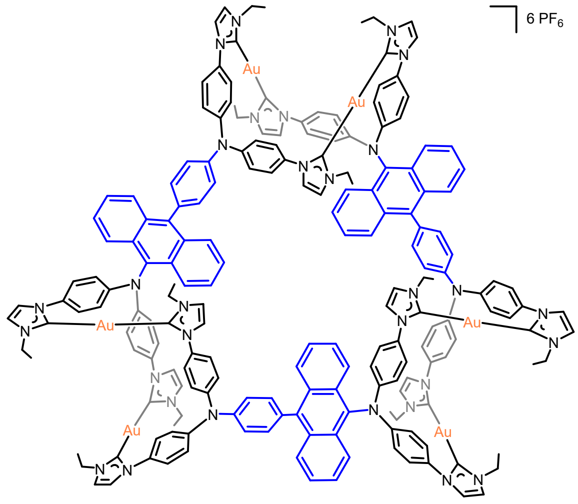

Solid [AuCl(THT)] (0.031 g, 0.097 mmol) was added to a solution of $[Ag_6(\mathbf{1c})_3](PF_6)_6$ (0.073 g, 0.0165 mmol) in dry acetonitrile (10 mL). The reaction mixture was stirred at ambient temperature for 24 h. The resulting suspension was centrifuged and the green solution was isolated by filtration. The filtrate was concentrated to 1 mL and diethyl ether (20 mL) was added leading to the precipitation of a gray solid. The precipitate was collected by filtration, washed with diethyl ether and dried *in vacuo* to give $[Au_6(\mathbf{1c})_3](PF_6)_6$. Yield: 0.075 g (0.0156 mmol, 94%). ^{1}H NMR (400 MHz, CD_3CN): δ = 7.62–7.33 (m, 96H), 7.08 (m, 12H), 4.38–4.32 (m, 24H), 1.58–1.49 (m, 36H). ESI-MS (positive ions): *m/z* = 679.5455 (calcd for $[Au_6(\mathbf{1c})_3]^{6+}$ 679.5354), 844.4354 (calcd for $[Au_6(\mathbf{1c})_3(PF_6)]^{5+}$ 844.4353).

5. Synthesis of ligand precursor H_4-1d(PF_6)$_4$ and of [Ag_8(1d)$_4$](PF_6)$_8$ and [Au_8(1d)$_4$](PF_6)$_8$

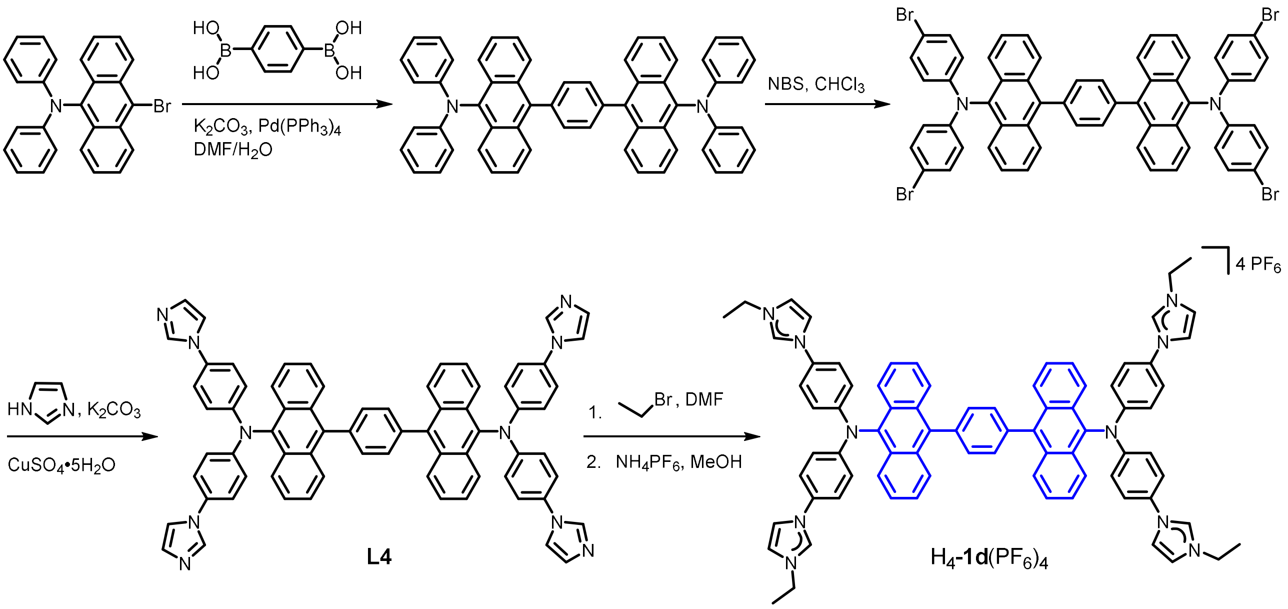

Scheme S4. Synthesis of the tetra-NHC precursor H_4-**1d**(PF_6)$_4$.

Synthesis of 10,10'-(1,4-phenylene)bis(*N*,*N*-diphenylanthracen-9-amine)

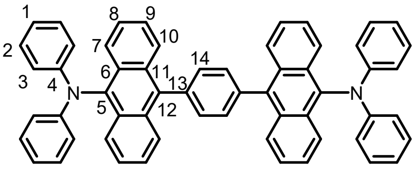

A 50 mL Schlenk flask was charged with the 1,4-phenylenebisboronic acid (0.037 g, 0.223 mmol), 10-bromo-*N*,*N*-diphenylanthracen-9-amine (0.200 g, 0.471mmol), DMF (10 mL), 3 mL of 1 *M* aqueous K_2CO_3 (0.415 g, 3.0 mmol) and Pd(PPh_3)$_4$ (0.04 g, 0.035 mmol). The mixture was heated to 100 °C for 24 h. After cooling to ambient temperature, the mixture was extracted with dichloromethane (3 × 20 mL). The organic phase was washed with water and dried over anhydrous Na_2SO_4. The solvent was then evaporated to give a yellow solid. Yield: 0.121 g (0.158 mmol, 71%). No additional purification was necessary.

Synthesis of 10,10'-(1,4-phenylene)bis(*N*,*N*-bis(4-bromophenyl)anthracen-9-amine)

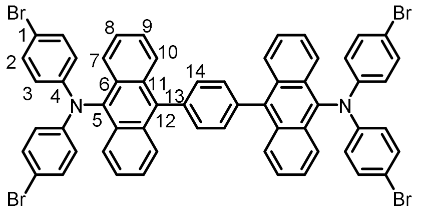

To a solution of 10,10'-(1,4-phenylene)bis(*N*,*N*-diphenylanthracen-9-amine) (0.340 g, 0.444 mmol) in 20 mL of $CHCl_3$ was added dropwise *N*-bromosuccinimide (0.348 g, 1.960 mmol) in 0.5 mL of DMF. The resulting mixture was stirred for 5 h at the ambient temperature. The formed solid residue was isolated by filtration and dried to give a

yellow solid. Yield: 0.440 g (0.407 mmol, 92%). ^{1}H NMR (400 MHz, $CDCl_3$): δ= 8.15–8.12 (m, 4H), 7.99–7.97 (m, 4H), 7.73 (s, 4H, H14), 7.52–7.48 (m, 8H), 7.32 (d, J = 8.0 Hz, 8H, H2), 7.02 (d, J = 8.0 Hz, 8H, H3). The solubility of the compound in common organic solvents was rather poor. Thus, the $^{13}C\{^1H\}$ NMR spectrum was not recorded.

Synthesis of ligand precursor H_4-1d(PF_6)$_4$

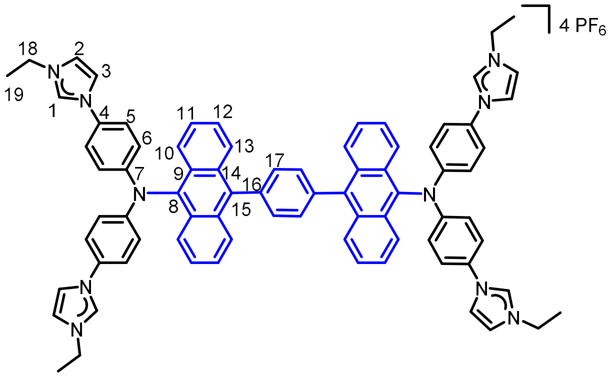

A sample of 10,10'-(1,4-phenylene)bis(*N*,*N*-bis(4-bromophenyl)-anthracene-9-amine) (0.301 g, 0.279 mmol), imidazole (8.0 g, 117.5 mmol), K_2CO_3 (8.0 g, 58.0 mmol), $CuSO_4 \cdot 5H_2O$ (0.500 g, 2.00 mmol) were mixed. The mixture was heated to 190 °C for 48 h. Then the reaction mixture was then cooled to ambient temperature and was washed several times with water. The remaining solid residue was brought to dryness to give **L4** as a pale-green solid. Yield: 0.270 g (0.262 mmol, 94%). A sample of **L4** (0.250 g, 0.243 mmol) and an excess of ethyl bromide 6.0 mL (8.76 g, 8.04 mmol) were mixed with 15 mL of DMF in a Schlenk flask. The reaction mixture was heated to 110 °C for 48 h. After cooling of the reaction mixture to ambient temperature, the solvent was removed. Subsequently, NH_4PF_6 (0.800 g, 4.91 mmol) in 20 mL of methanol was added. The mixture was stirred for 24 h at ambient temperature. Over this period, the hexafluorophosphate salt H_4-**1d**(PF_6)$_4$ precipitated. The solid was collected by filtration, washed with methanol and dried *in vacuo*. Yield: 0.290 g (0.168 mmol, 69%). ^{1}H NMR (400 MHz, CD_3CN): δ= 8.82 (s, 4H, H1), 8.23–8.20 (m, 4H, H11), 8.10–8.07 (m, 4H, H12), 7.79 (s, 4H, H17), 7.69 (s, 4H, H3), 7.61–7.58 (m, 8H, H13, H10), 7.57 (s, 4H, H2), 7.51 (d, J = 8.0 Hz, 8H, H5), 7.39 (d, J = 8.0 Hz, 8H, H6), 4.26 (q, J = 8.0 Hz, 8H, H18), 1.53 (t, J = 8.0 Hz, 12H, H19). $^{13}C\{^1H\}$ NMR (100 MHz, CD_3CN): δ= 149.2 (C4), 140.0 (C14), 139.0 (C16), 136.4 (C9), 135.1 (C1), 132.6 (C15), 132.3 (C17), 130.9 (C8), 129.9 (C7), 129.0 (C12), 128.7 (C13), 127.2 (C10), 124.7 (C5), 124.2 (C11), 123.8 (C2), 122.7 (C3), 122.2 (C6), 46.4 (C18), 15.3 (C19). ESI-MS (positive ions): m/z = 286.1442 (calcd for [H_4-**1d**]$^{4+}$ 286.1407), 429.8531 (calcd for [H_4-**1d**(PF_6)]$^{3+}$ 429.8423).

Synthesis of $[Ag_8(\mathbf{1d})_4](PF_6)_8$

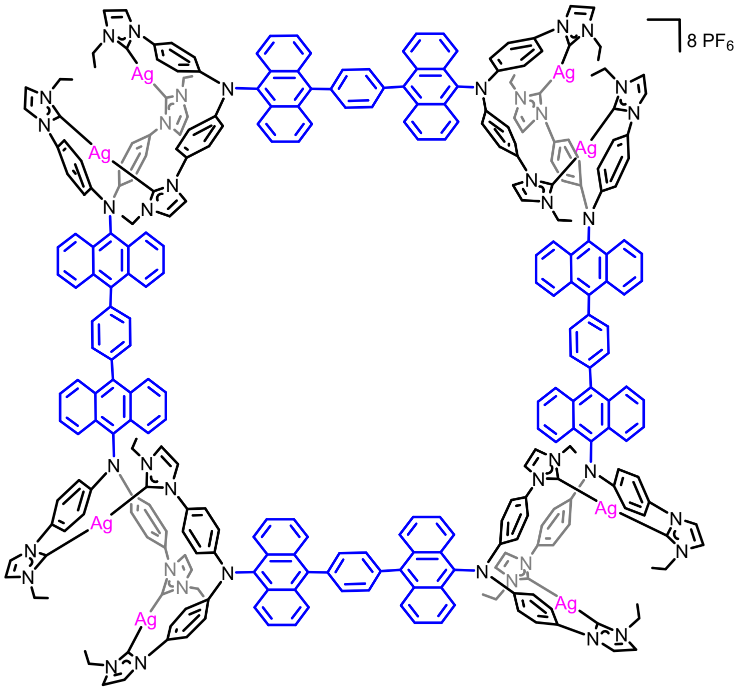

To a mixture of H_4-**1d**$(PF_6)_4$ (0.073 g, 0.042 mmol) and Ag_2O (0.036 g, 0.155 mmol) was added 20 mL dry acetonitrile. The reaction mixture was heated to 80 °C for 36 h under exclusion of light. After cooling of the reaction mixture to ambient temperature, the resulting suspension was centrifuged and the green solution was separated by filtration. The filtrate was concentrated to 1 mL, and diethyl ether (20 mL) was added leading to precipitation of $[Ag_8(\mathbf{1d})_4](PF_6)_8$ as a gray solid. The solid was collected by filtration, washed with diethyl ether and dried *in vacuo*. Yield: 0.068 g (0.0103 mmol, 98%). ^{1}H NMR (400 MHz, CD_3CN): δ = 8.35 (d, J = 8.0 Hz, 8H), 8.21 (d, J = 4.0 Hz, 8H), 8.10–8.01 (m, 16H), 7.77 (s, 32H), 7.64–7.61 (m, 24H), 7.48–7.30 (m, 64H), 7.17–7.10 (m, 24H), 4.28–4.24 (m, 32H), 1.54–1.51 (m, 48H). ESI-MS (positive ions): *m/z* = 678.5414 (calcd for $[Ag_8(\mathbf{1d})_4]^{8+}$ 678.5470), 796.1883 (calcd for $[Ag_8(\mathbf{1d})_4(PF_6)]^{7+}$ 796.1915), 953.0233 (calcd for $[Ag_8(\mathbf{1d})_4(PF_6)_2]^{6+}$ 953.0508).

Synthesis of $[Au_8(\mathbf{1d})_4](PF_6)_8$

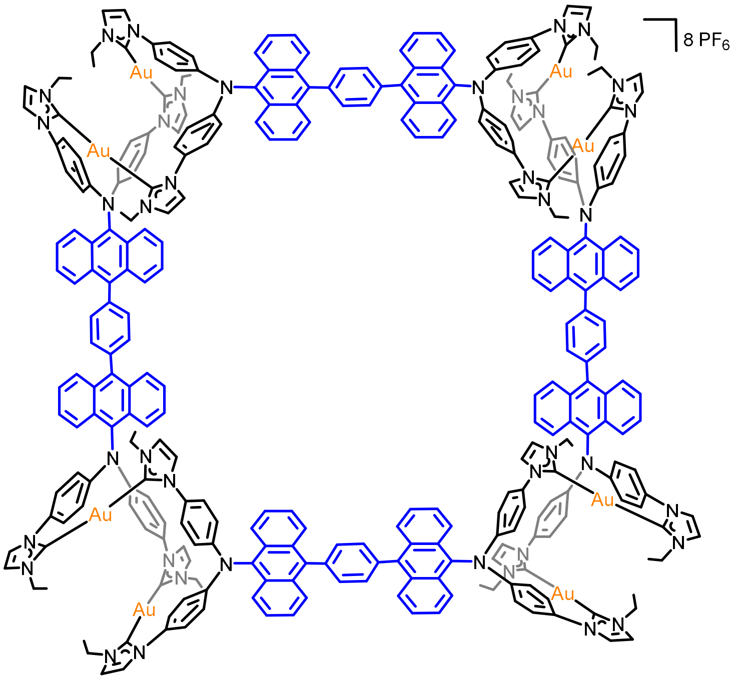

Solid [AuCl(THT)] (0.025 g, 0.078 mmol) was added to a solution of $[Ag_8(\mathbf{1d})_4](PF_6)_8$ (0.068 g, 0.0103 mmol) in dry acetonitrile (20 mL). The reaction mixture was stirred at ambient temperature for 24 h. The resulting suspension was centrifuged and the green solution was separated by filtration. The filtrate was concentrated to 1 mL and diethyl ether (20 mL) was added resulting in the precipitation of $[Au_8(\mathbf{1d})_4](PF_6)_8$ as a gray solid. The solid was collected by filtration, washed with diethyl ether and dried *in vacuo*. Yield: 0.032 g (0.004 mmol, 39%). ^{1}H NMR (400 MHz, CD_3CN): δ = 8.38 (d, J = 8.0 Hz, 8H), 8.22–8.19 (m, 8H), 8.10–8.01 (m, 16H), 7.86 (d, J = 8.0 Hz, 16H), 7.78 (d, J = 8.0 Hz, 16H), 7.65–7.59 (m, 24H), 7.48–7.35 (m, 48H), 7.30 (d, J = 8.0 Hz, 16H), 7.22–7.18 (m, 8H), 7.11 (d, J = 8.0 Hz, 16H), 4.37–4.25 (m, 32H), 1.56–1.44 (m, 48H). ESI-MS (positive ions): m/z = 767.5905 (calcd for $[Au_8(\mathbf{1d})_4]^{8+}$ 767.6084), 897.9578 (calcd for $[Au_8(\mathbf{1d})_4(PF_6)]^{7+}$ 897.9760).

6. Selected NMR and ESI-MS spectra of new compounds

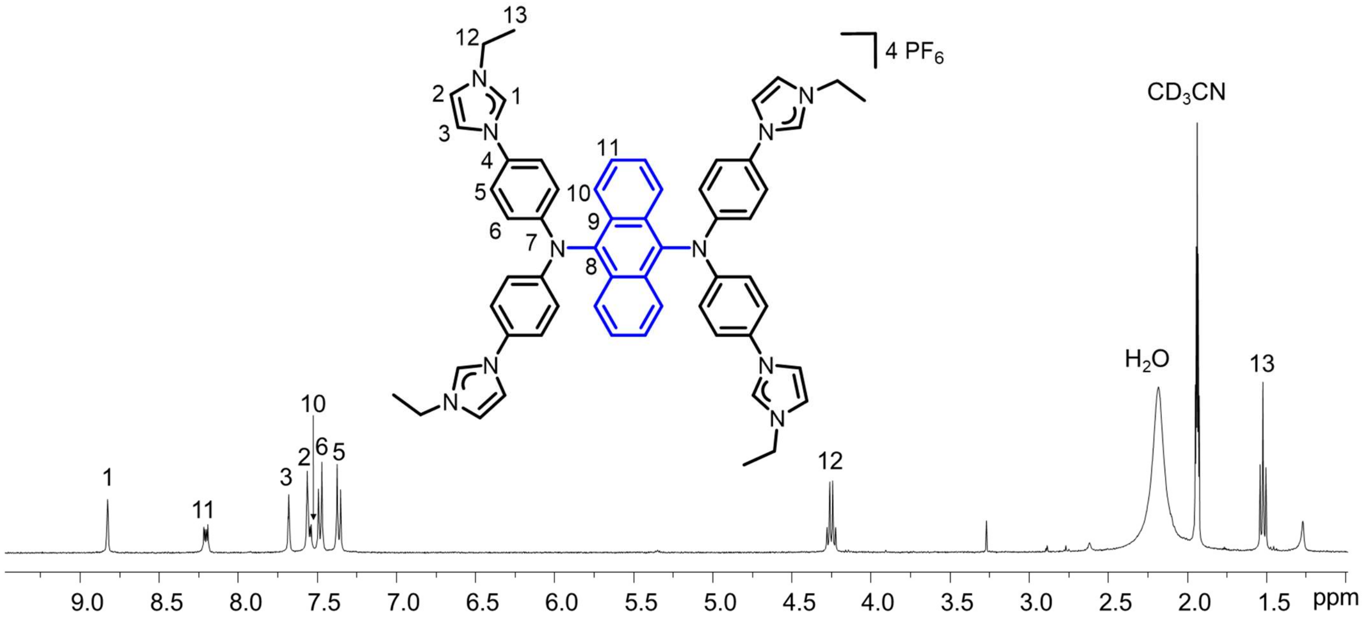

Figure S1. ^{1}H NMR spectrum of H_4-**1a**$(PF_6)_4$ (400 MHz, CD_3CN, 298 K).

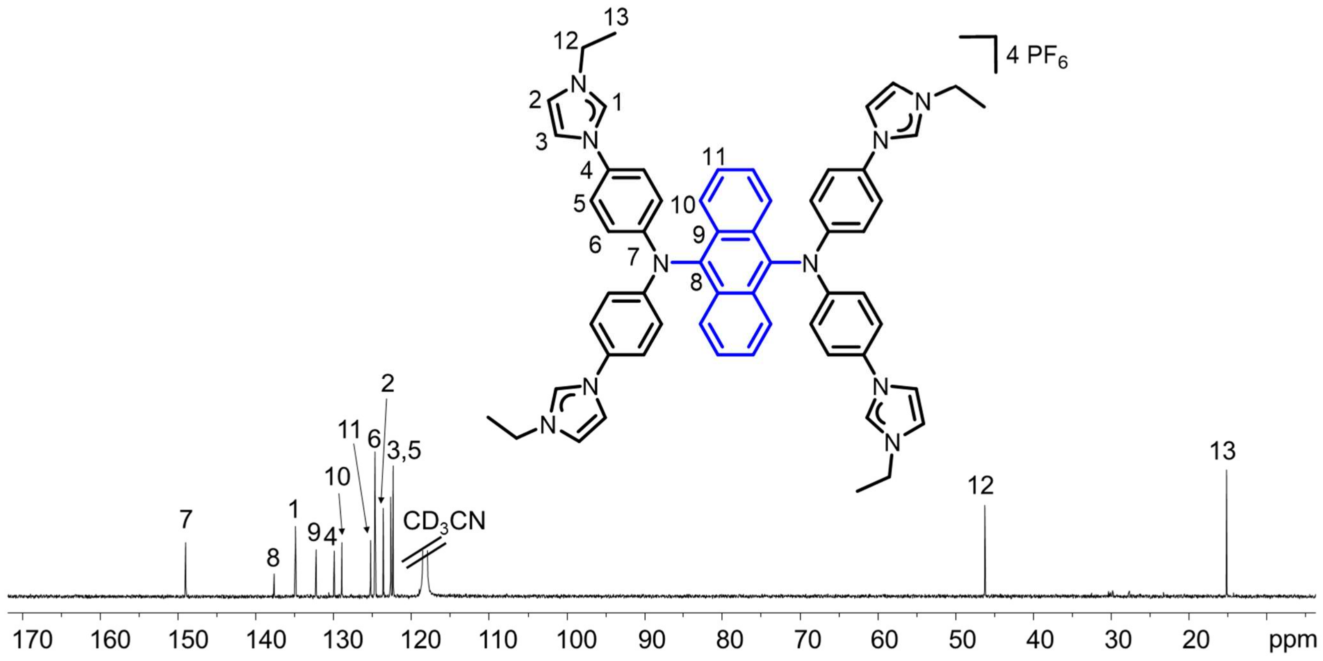

Figure S2. $^{13}C\{^{1}H\}$ NMR spectrum of H_4-**1a**$(PF_6)_4$ (100 MHz, CD_3CN, 298 K).

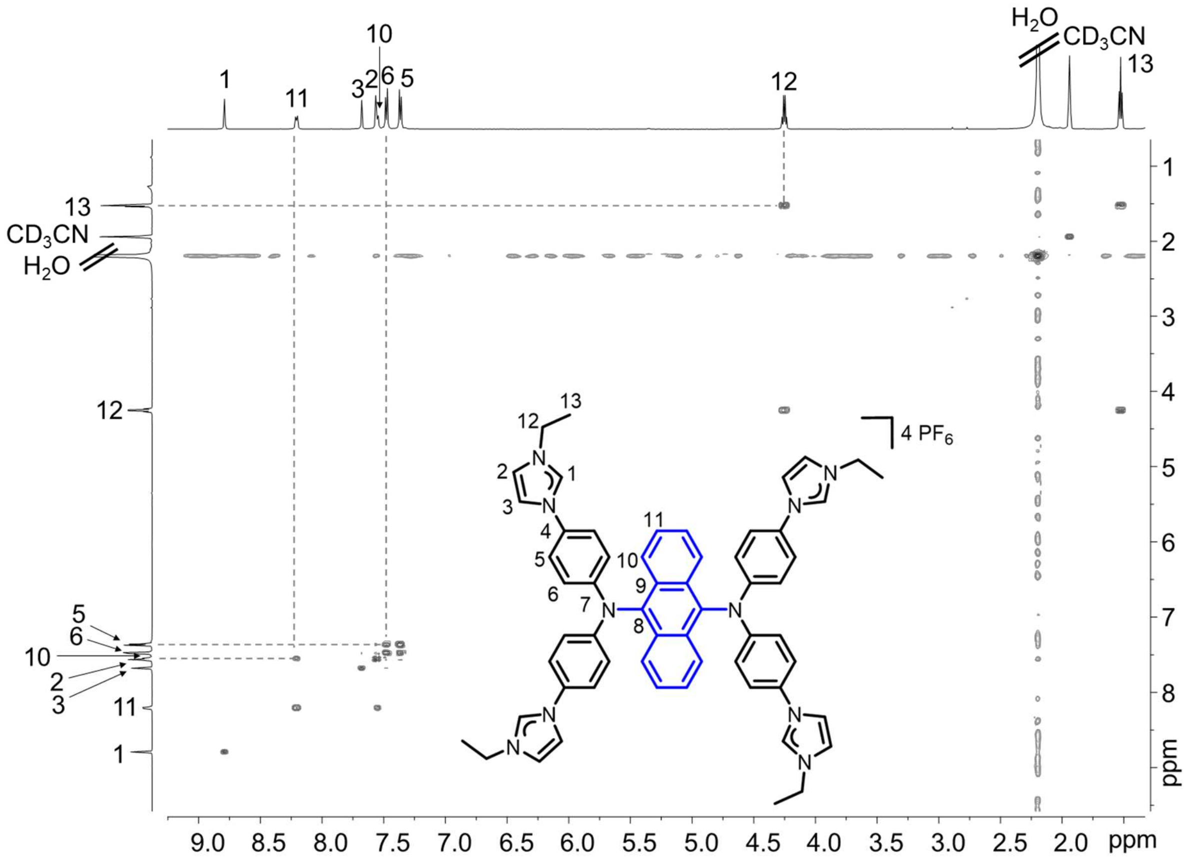

Figure S3. ^{1}H-^{1}H COSY spectrum of H_4-**1a**$(PF_6)_4$ (400 MHz, CD_3CN, 298 K).

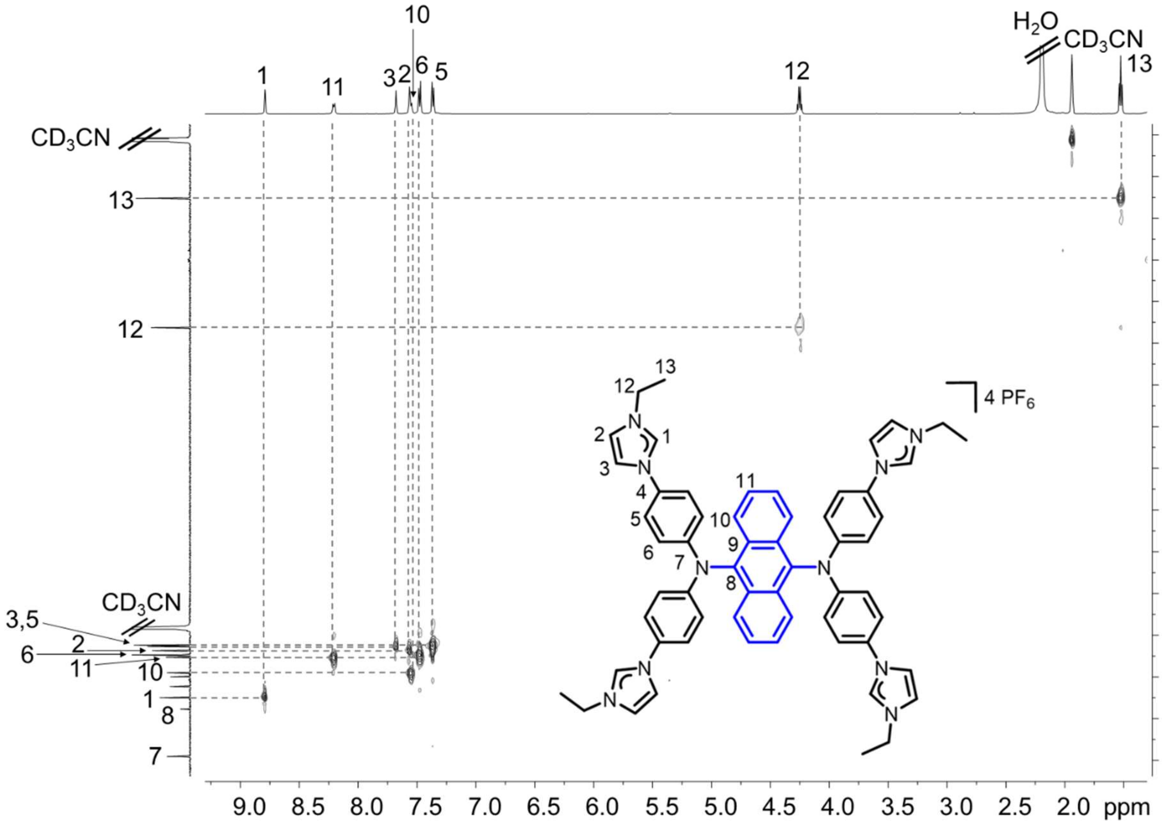

Figure S4. ^{1}H-^{13}C HSQC spectrum of H_4-**1a**$(PF_6)_4$ (400 MHz, CD_3CN, 298 K).

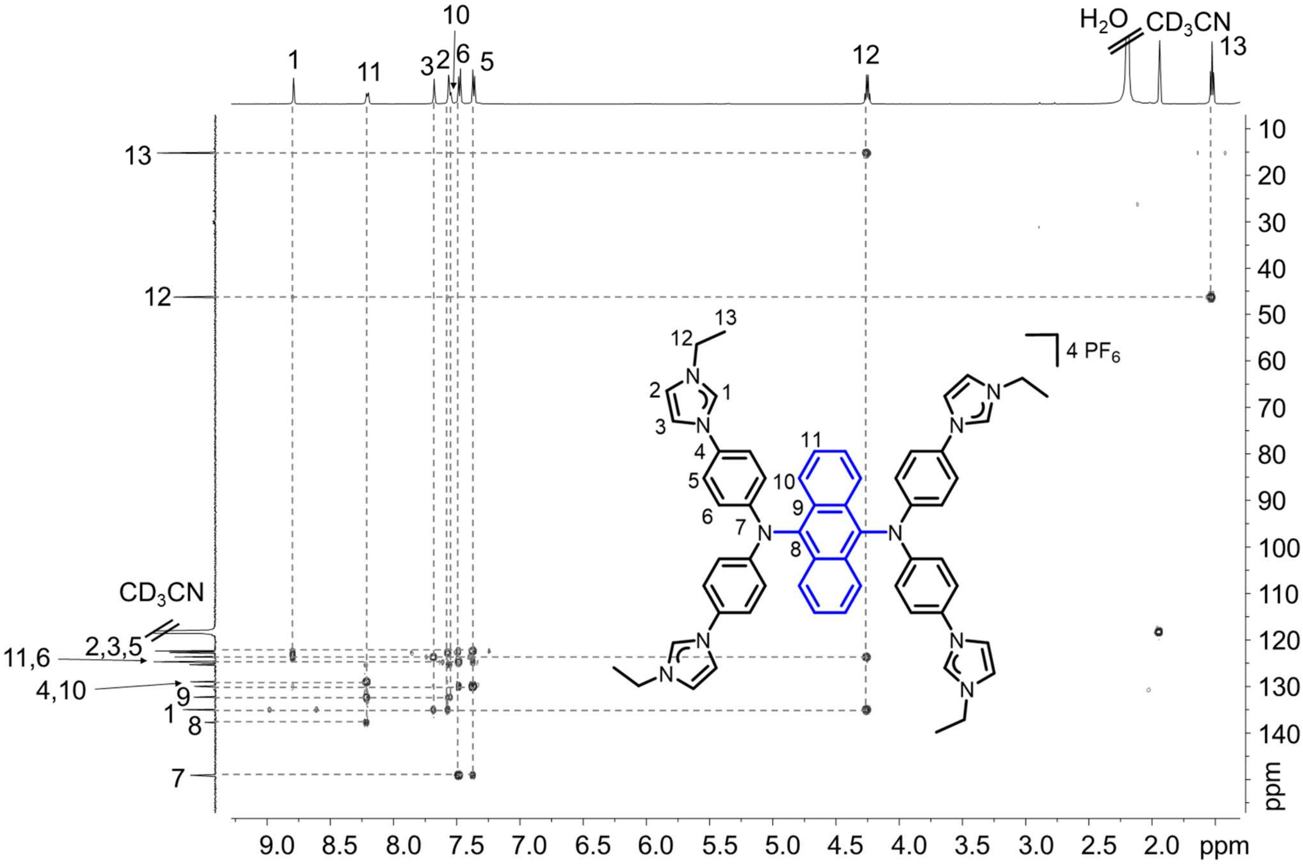

Figure S5. ^{1}H-^{13}C HMBC spectrum of H_4-**1a**$(PF_6)_4$ (400 MHz, CD_3CN, 298 K).

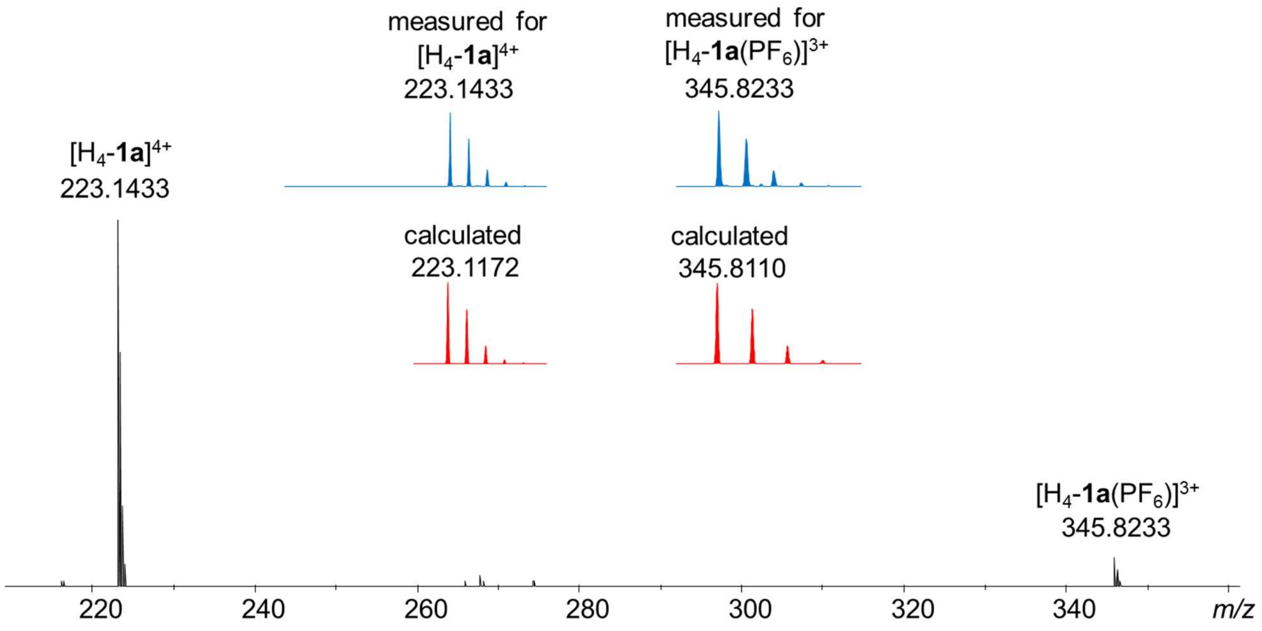

Figure S6. ESI mass spectrum (positive ions) of H_4-**1a**$(PF_6)_4$. Isotope distribution of selected cations are shown as inset (experimentally measured distribution on top and calculated distribution at the bottom).

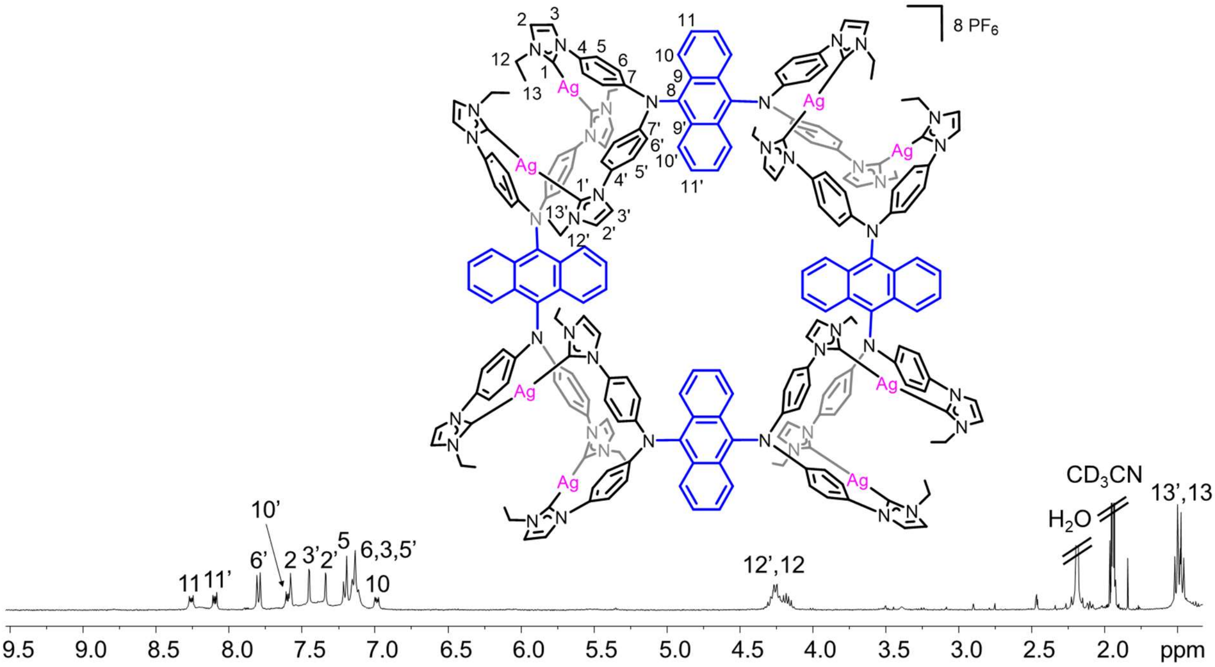

Figure S7. ^{1}H NMR spectrum of $[Ag_8(\mathbf{1a})_4](PF_6)_8$ (400 MHz, CD_3CN, 298 K).

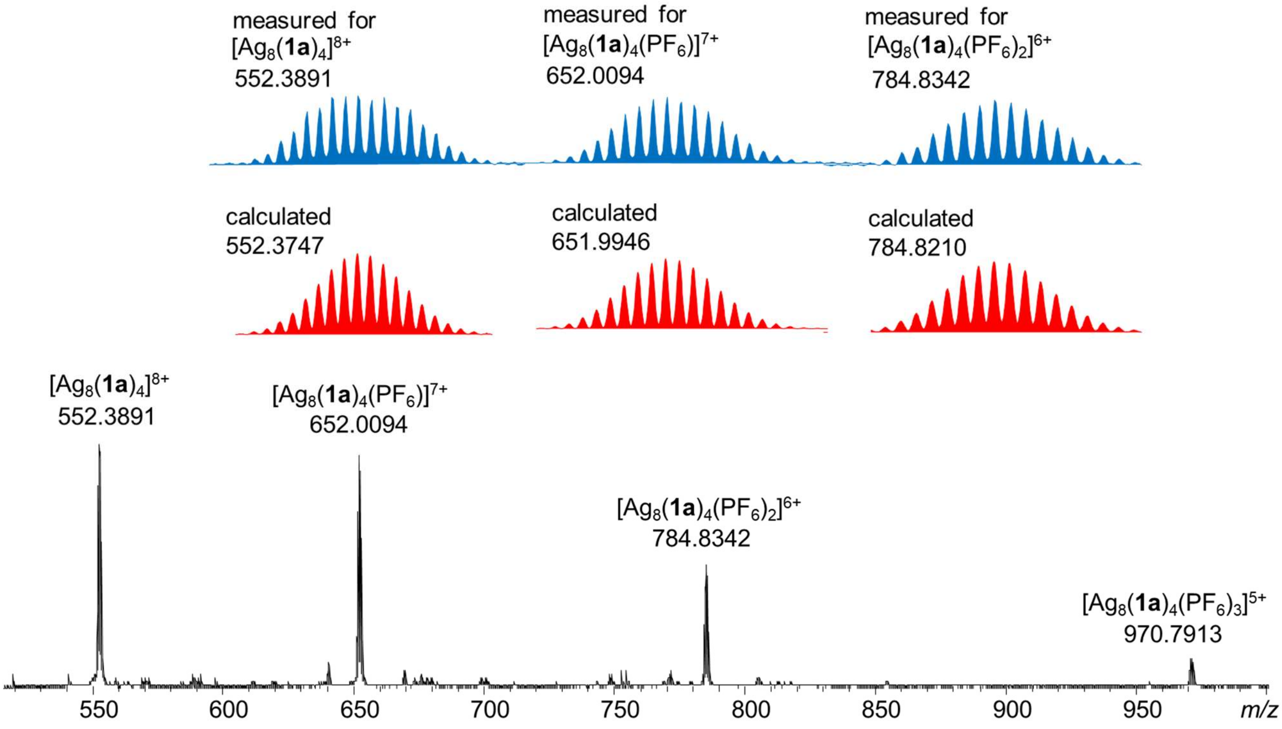

Figure S8. ESI mass spectrum (positive ions) of $[Ag_8(\mathbf{1a})_4](PF_6)_8$. Isotope distribution of selected cations are shown as inset (experimentally measured distribution on top and calculated distribution at the bottom).

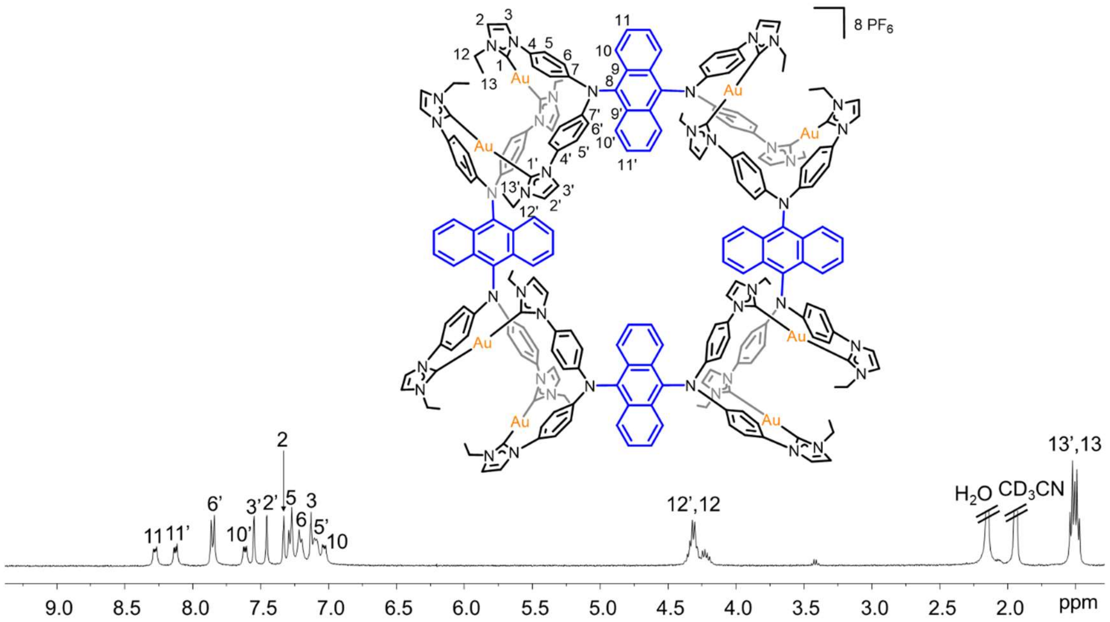

Figure S9. ^{1}H NMR spectrum of $[Au_8(\mathbf{1a})_4](PF_6)_8$ (400 MHz, CD_3CN, 298 K).

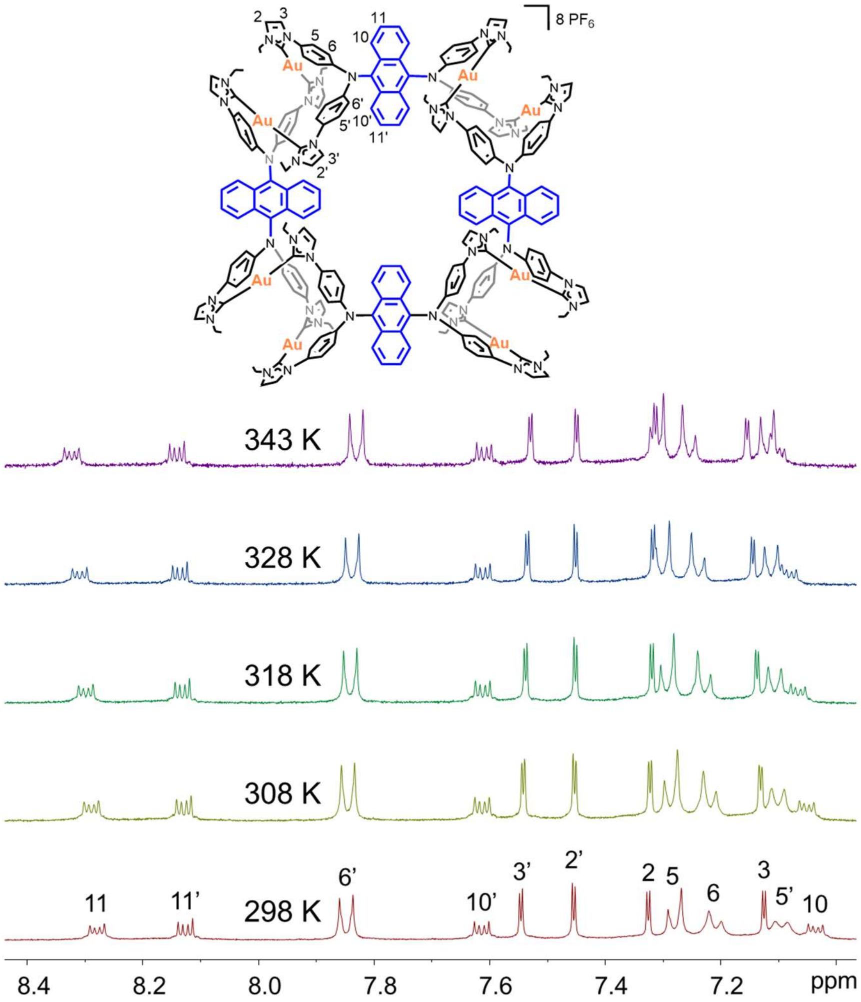

Figure S10. Sections of the ^{1}H VT NMR spectra of $[Au_8(\mathbf{1a})_4](PF_6)_8$ (400 MHz, CD_3CN, 298 K).

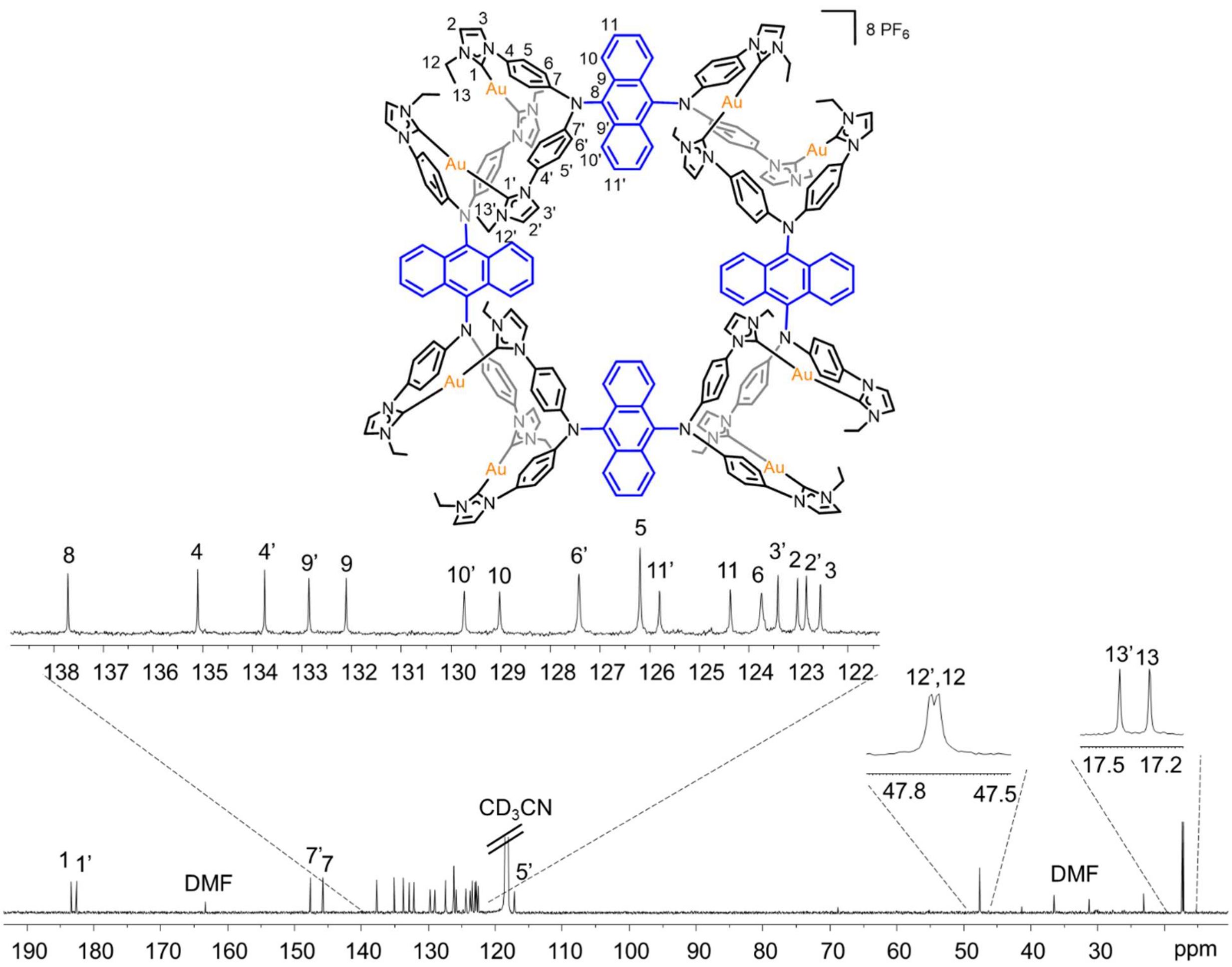

Figure S11. $^{13}C\{^1H\}$ NMR spectrum of $[Au_8(\mathbf{1a})_4](PF_6)_8$ (100 MHz, CD_3CN, 298 K).

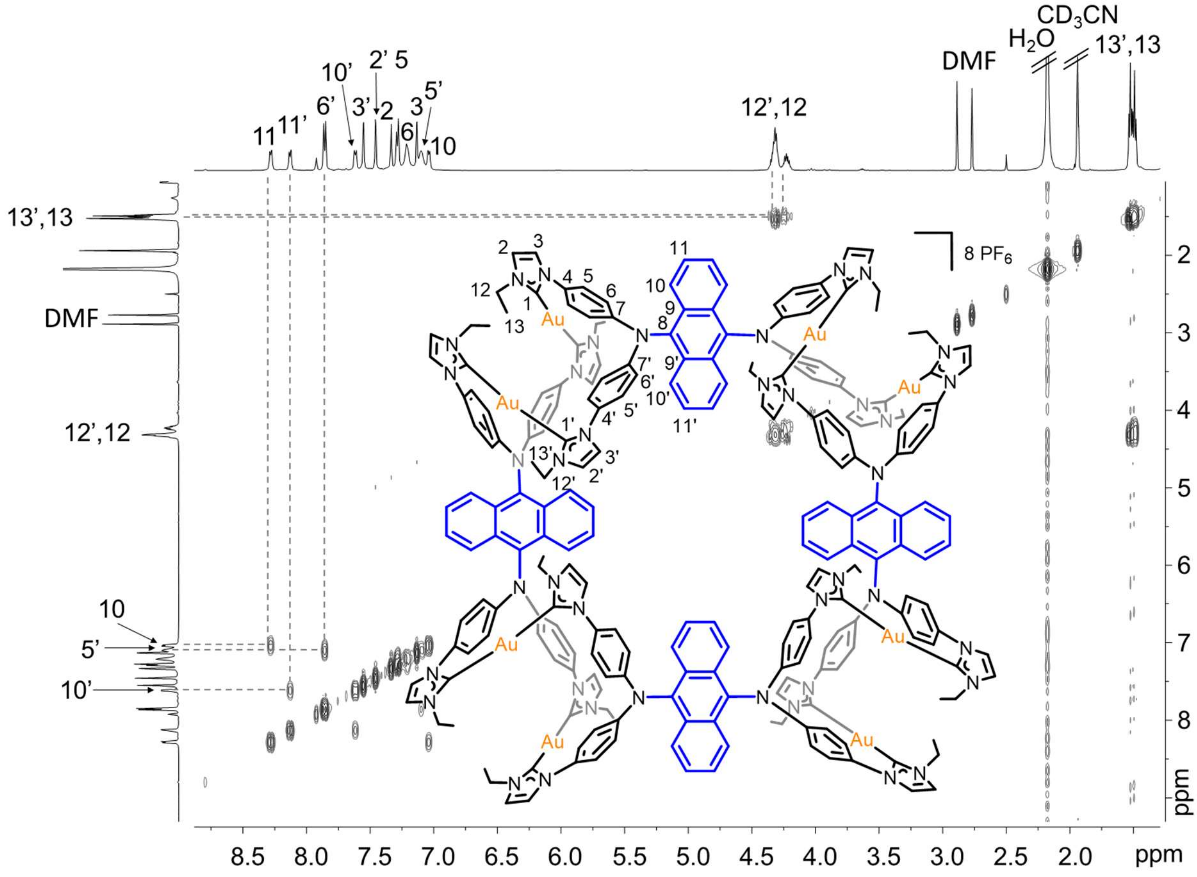

Figure S12. ^{1}H-^{1}H COSY spectrum of [Au_8(**1a**)$_4$](PF_6)$_8$ (400 MHz, CD_3CN, 298 K).

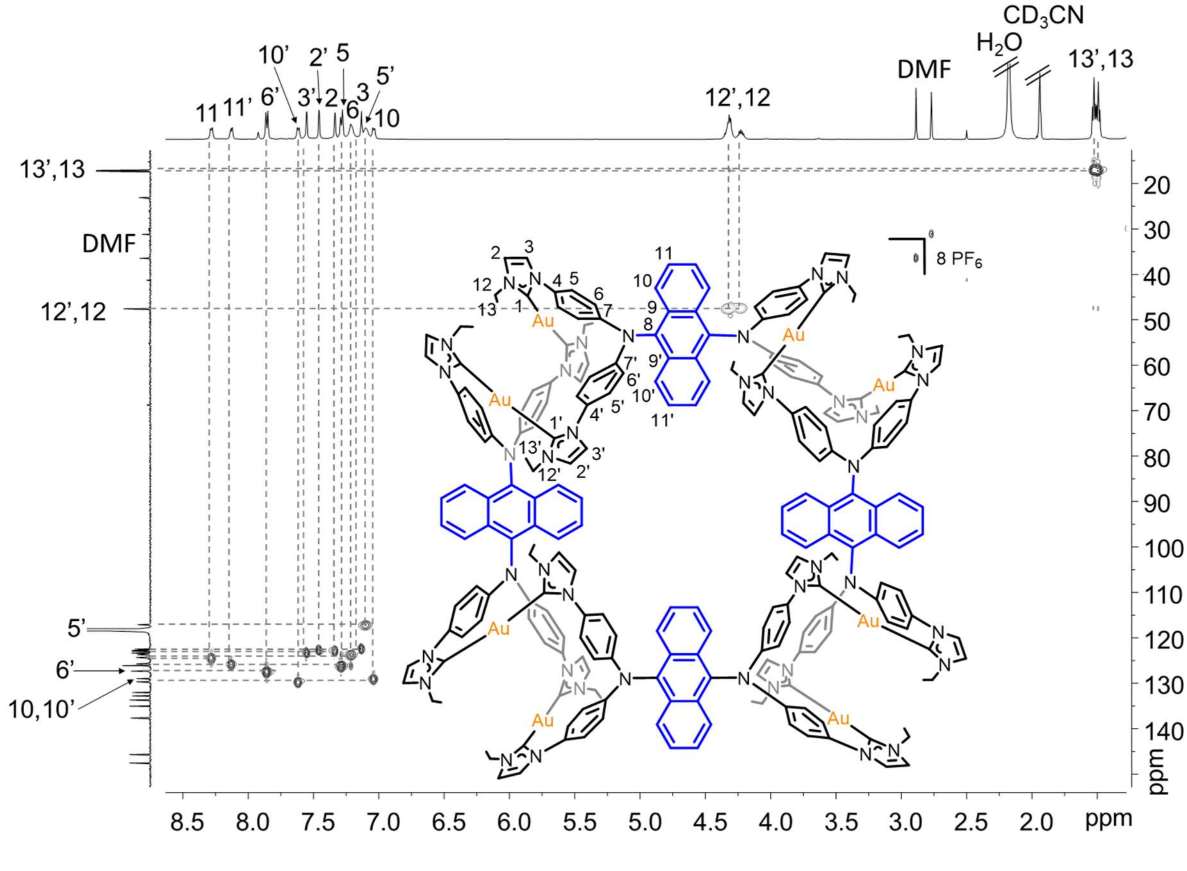

Figure S13. ^{1}H-^{13}C HSQC spectrum of [Au_8(**1a**)$_4$](PF_6)$_8$ (400 MHz, CD_3CN, 298 K).

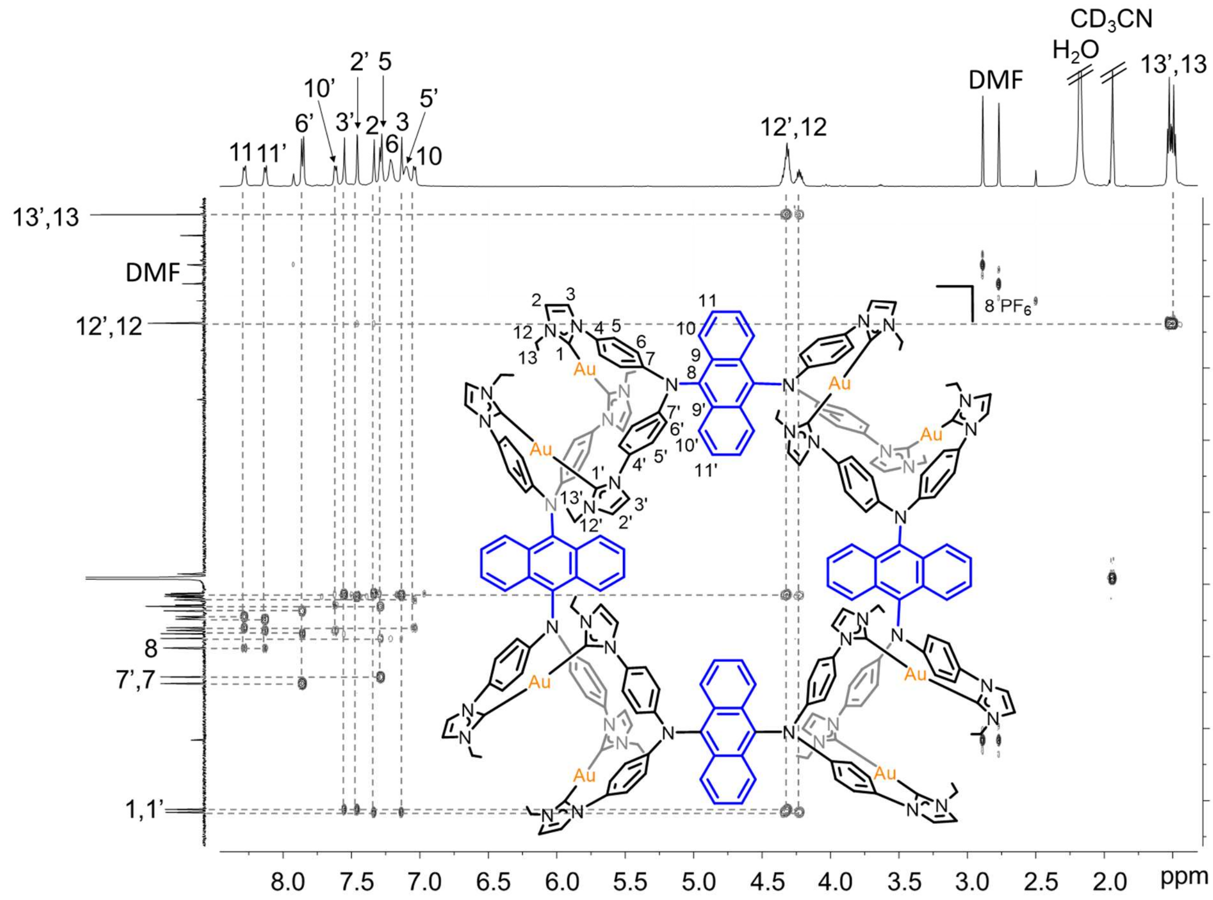

Figure S14. ^{1}H-^{13}C HMBC spectrum of $[Au_8(\mathbf{1a})_4](PF_6)_8$ (400 MHz, CD_3CN, 298 K).

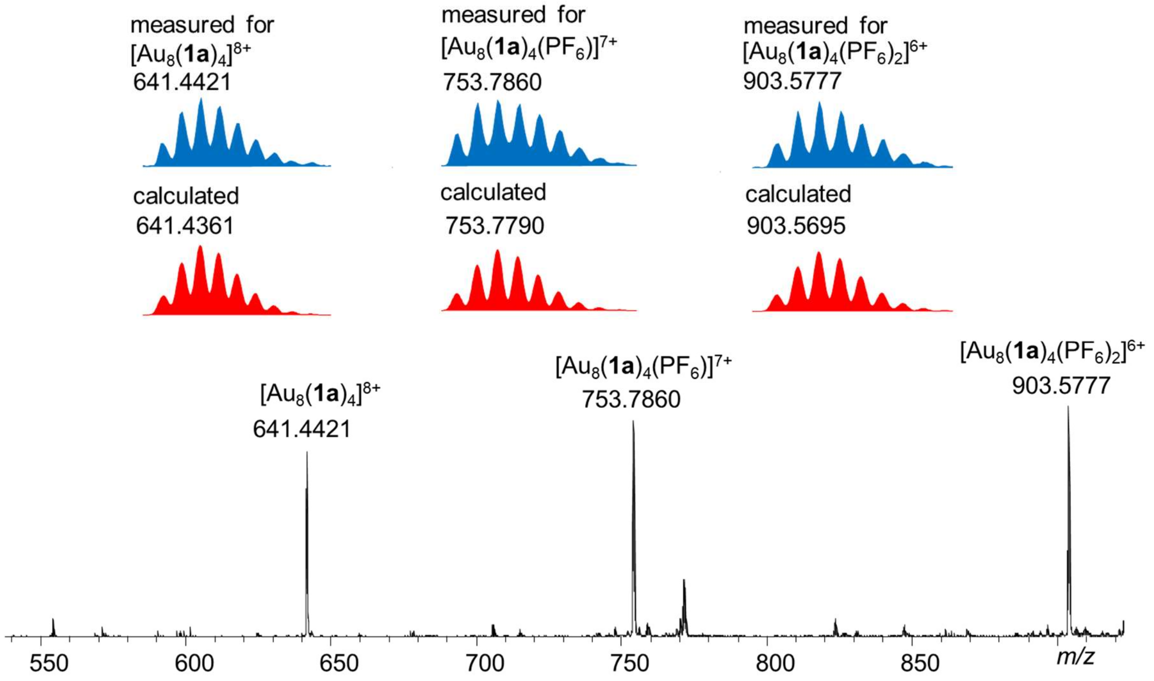

Figure S15. ESI mass spectrum (positive ions) of $[Au_8(\mathbf{1a})_4](PF_6)_8$. Isotope distribution of selected cations are shown as inset (experimentally measured distribution on top and calculated distribution at the bottom).

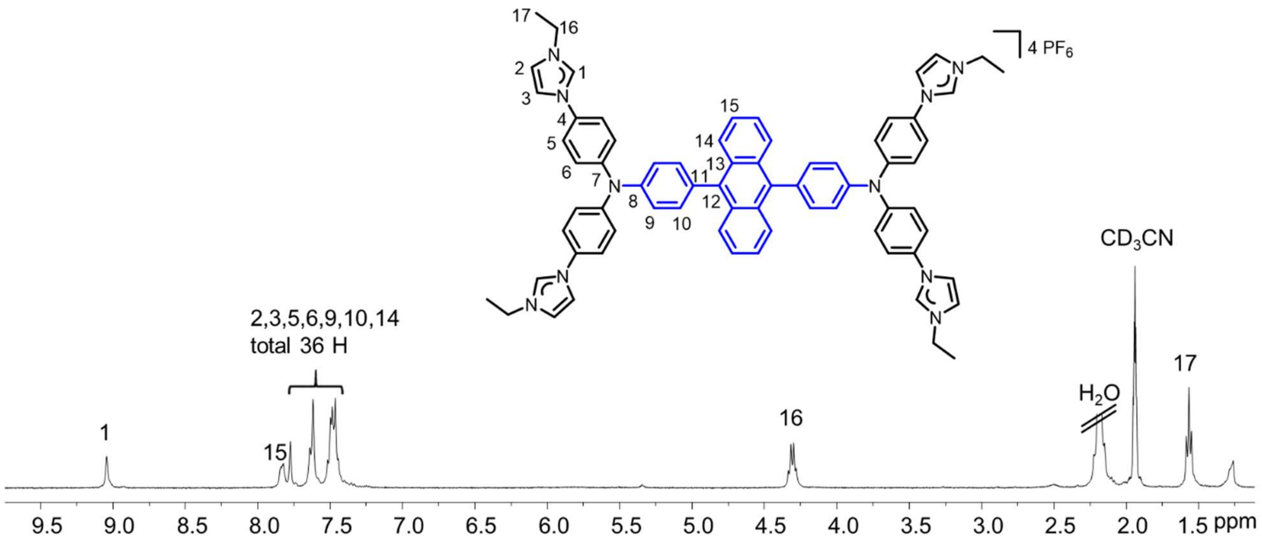

Figure S16. ^{1}H NMR spectrum of H$_4$-**1b**(PF$_6$)$_4$ (400 MHz, CD$_3$CN, 298 K).

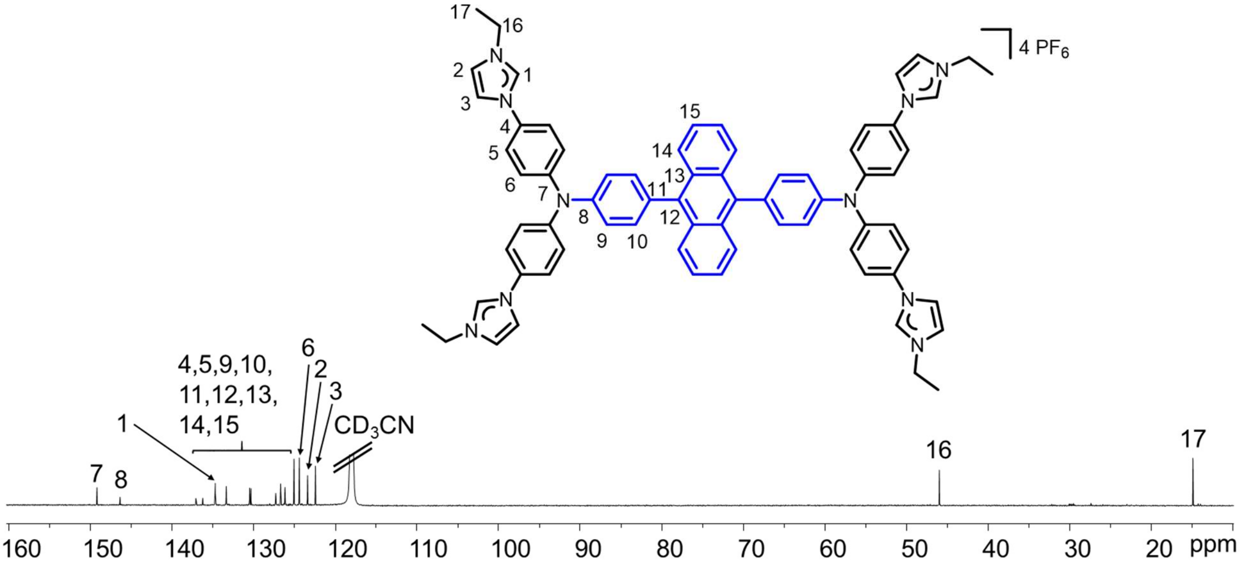

Figure S17. ^{13}C{^{1}H} NMR spectrum of H$_4$-**1b**(PF$_6$)$_4$ (100 MHz, CD$_3$CN, 298 K).

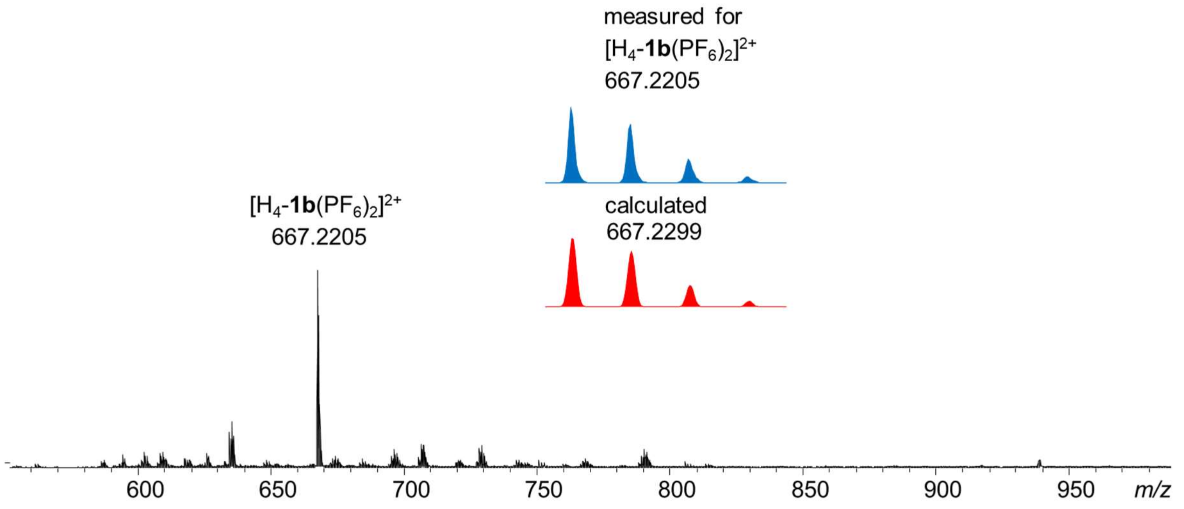

Figure S18. ESI mass spectrum (positive ions) of H_4-**1b**$(PF_6)_4$. Isotope distribution of a selected cation is shown as inset (experimentally measured distribution on top and calculated distribution at the bottom).

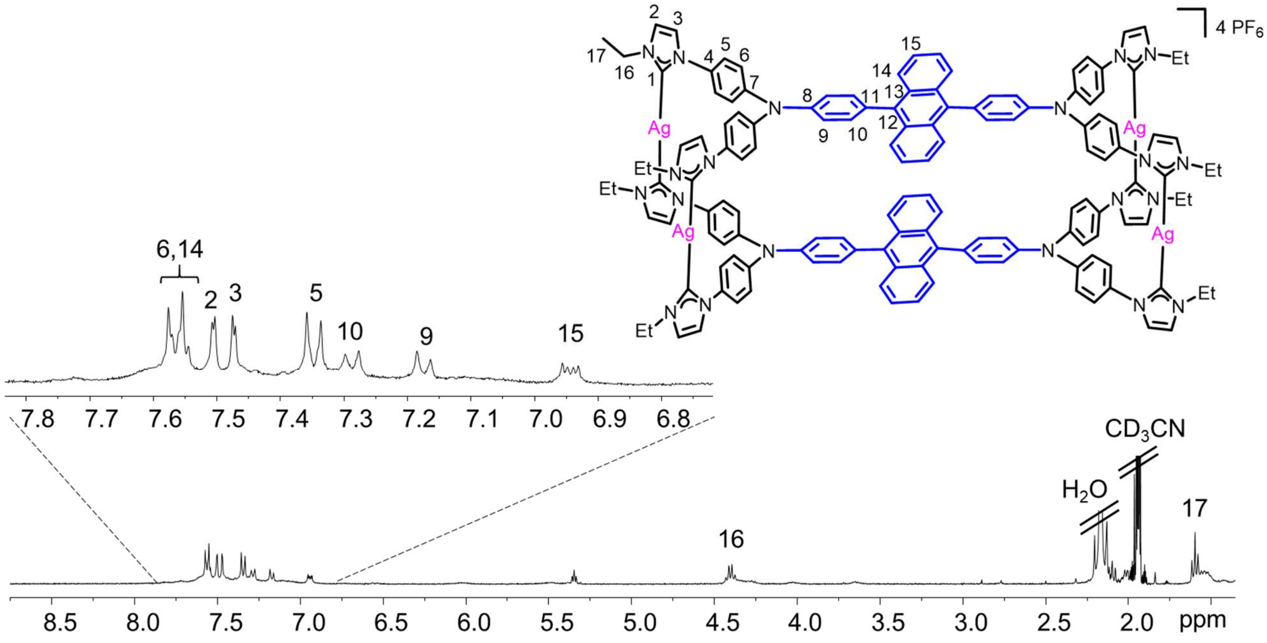

Figure S19. ^{1}H NMR spectrum of $[Ag_4(\mathbf{1b})_2](PF_6)_4$ (400 MHz, CD_3CN, 298 K).

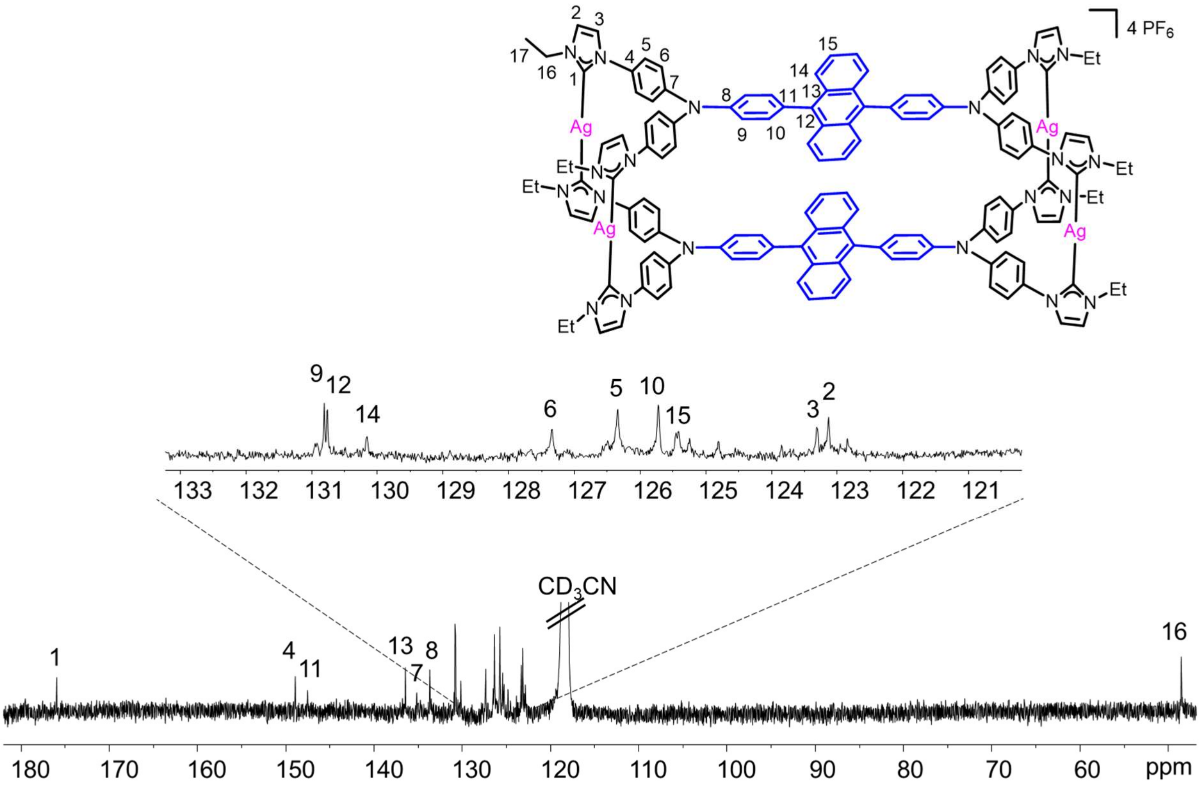

Figure S20. $^{13}C\{^{1}H\}$ NMR spectrum of $[Ag_4(\mathbf{1b})_2](PF_6)_4$ (100 MHz, CD_3CN, 298 K).

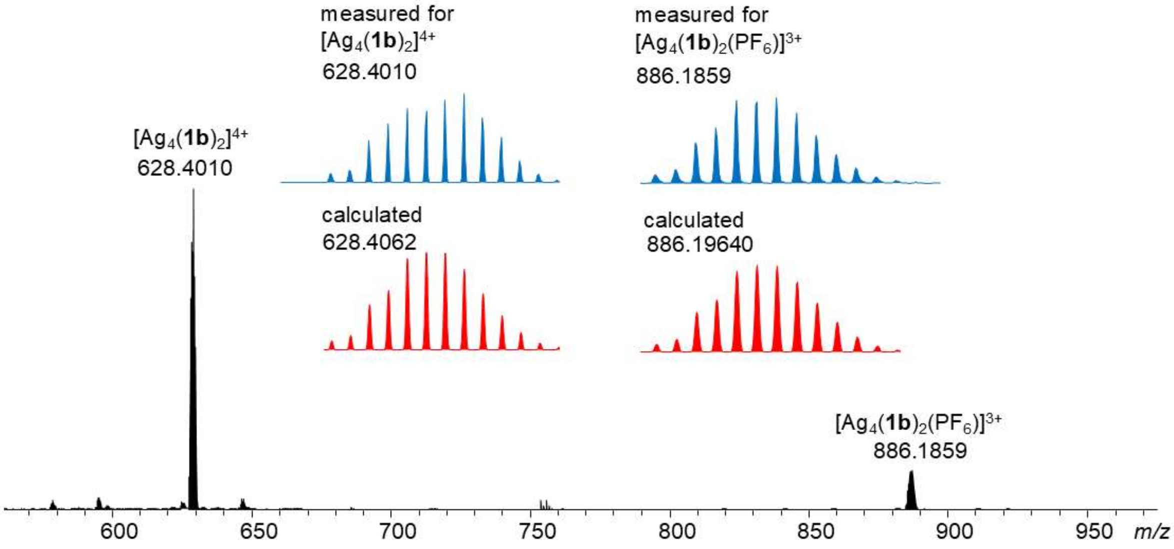

Figure S21. ESI mass spectrum (positive ions) of $[Ag_4(\mathbf{1b})_2](PF_6)_4$. Isotope distribution of selected cations are shown as inset (experimentally measured distribution on top and calculated distribution at the bottom).

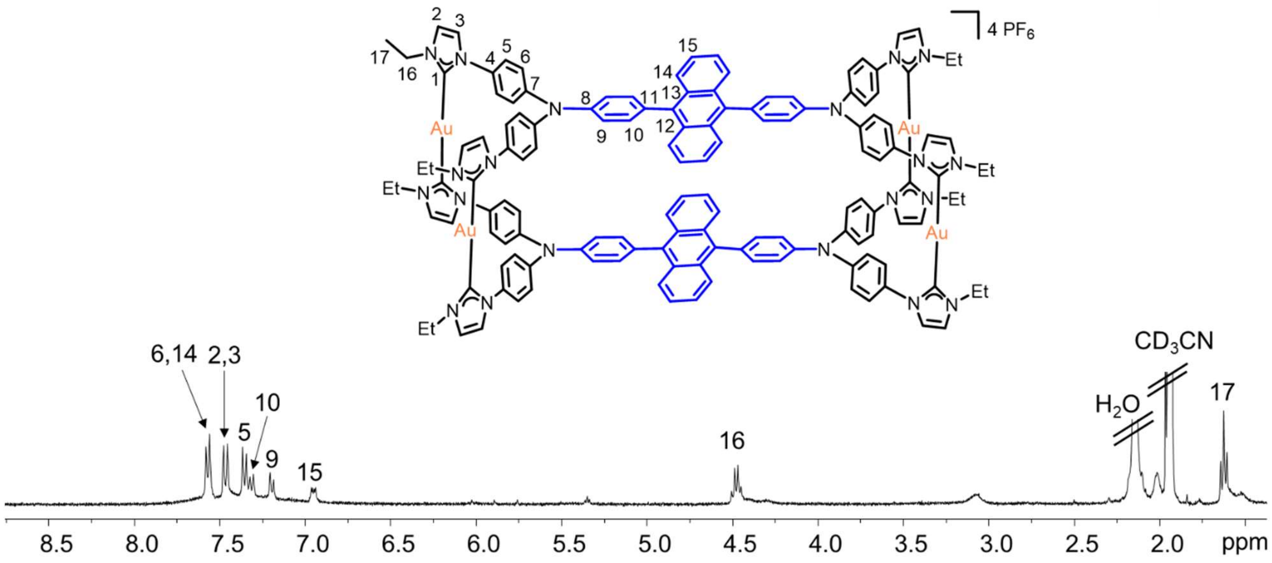

Figure S22. ^{1}H NMR spectrum of $[Au_4(\mathbf{1b})_2](PF_6)_4$ (400 MHz, CD_3CN, 298 K).

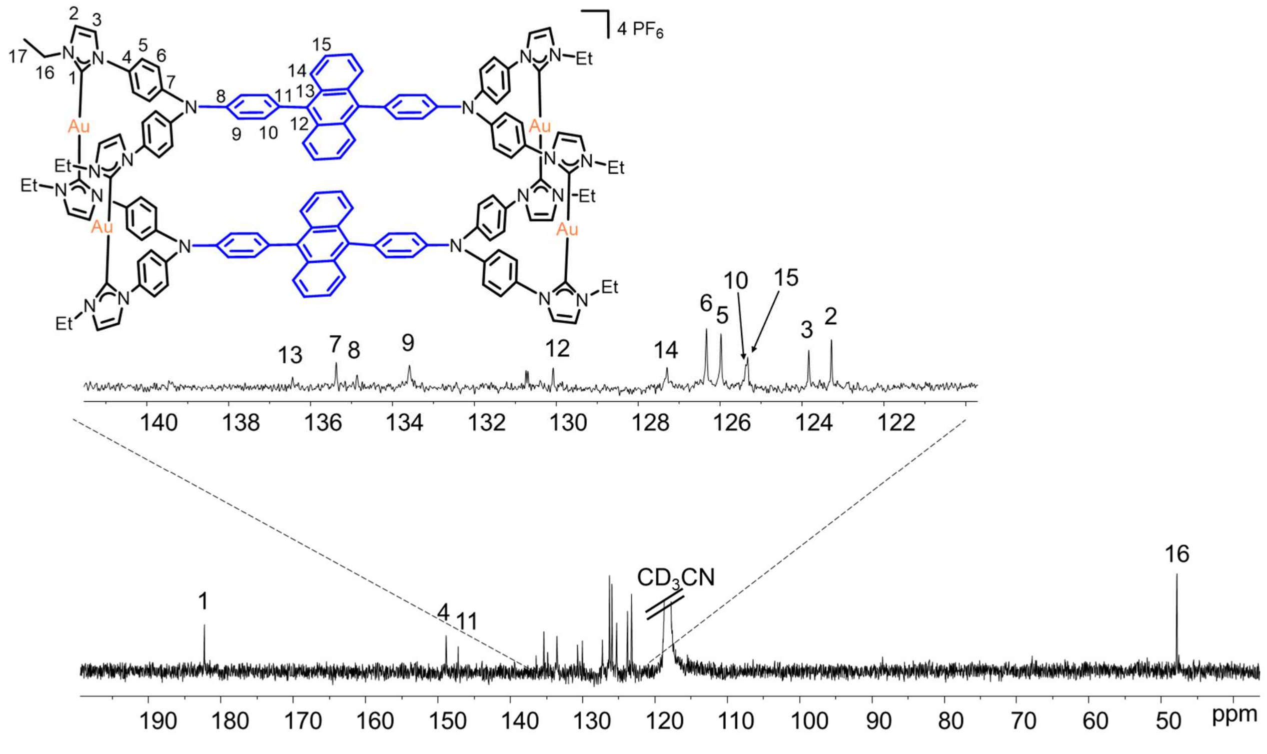

Figure S23. $^{13}C\{^{1}H\}$ NMR spectrum of $[Au_4(\mathbf{1b})_2](PF_6)_4$ (100 MHz, CD_3CN, 298 K).

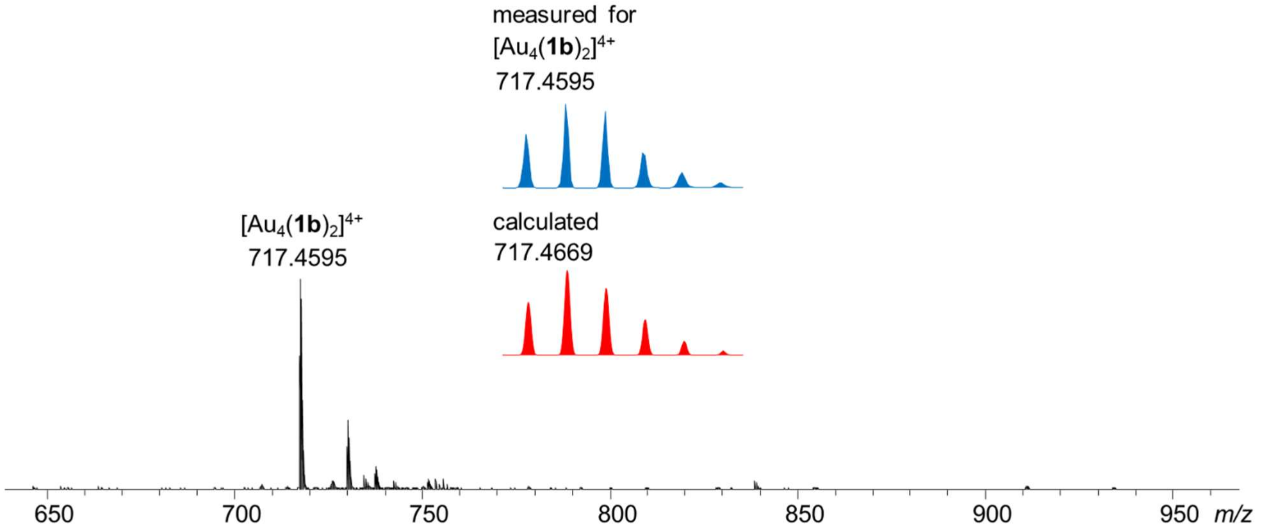

Figure S24. ESI mass spectrum (positive ions) of $[Au_4(\mathbf{1b})_2](PF_6)_4$. Isotope distribution of a selected cation is shown as inset (experimentally measured distribution on top and calculated distribution at the bottom).

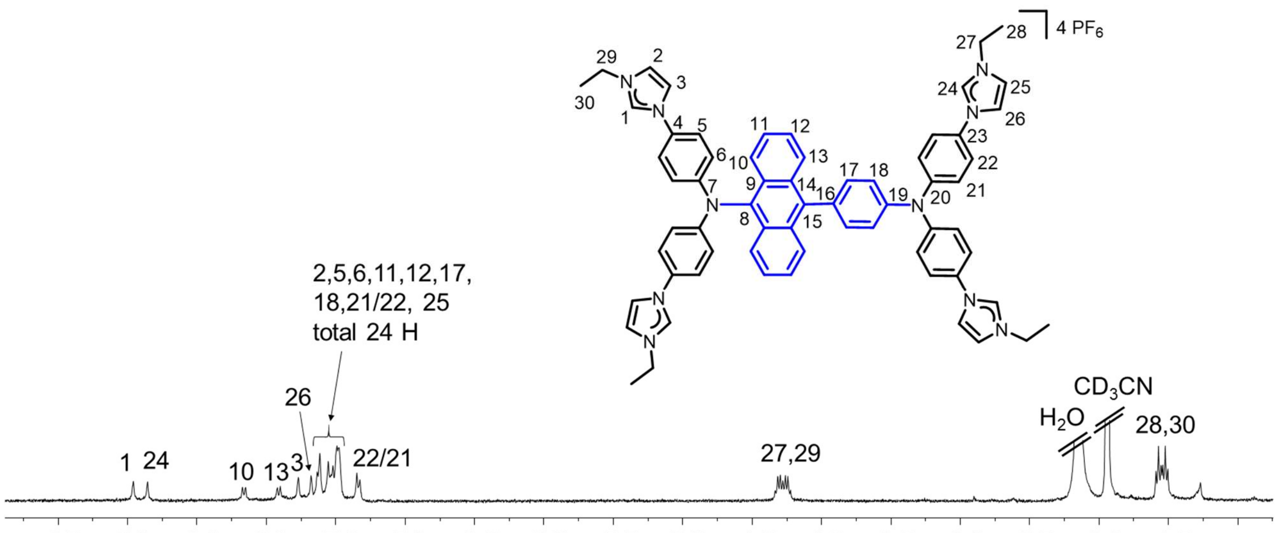

Figure S25. ^{1}H NMR spectrum of H_4-**1c**$(PF_6)_4$ (400 MHz, CD_3CN, 298 K).

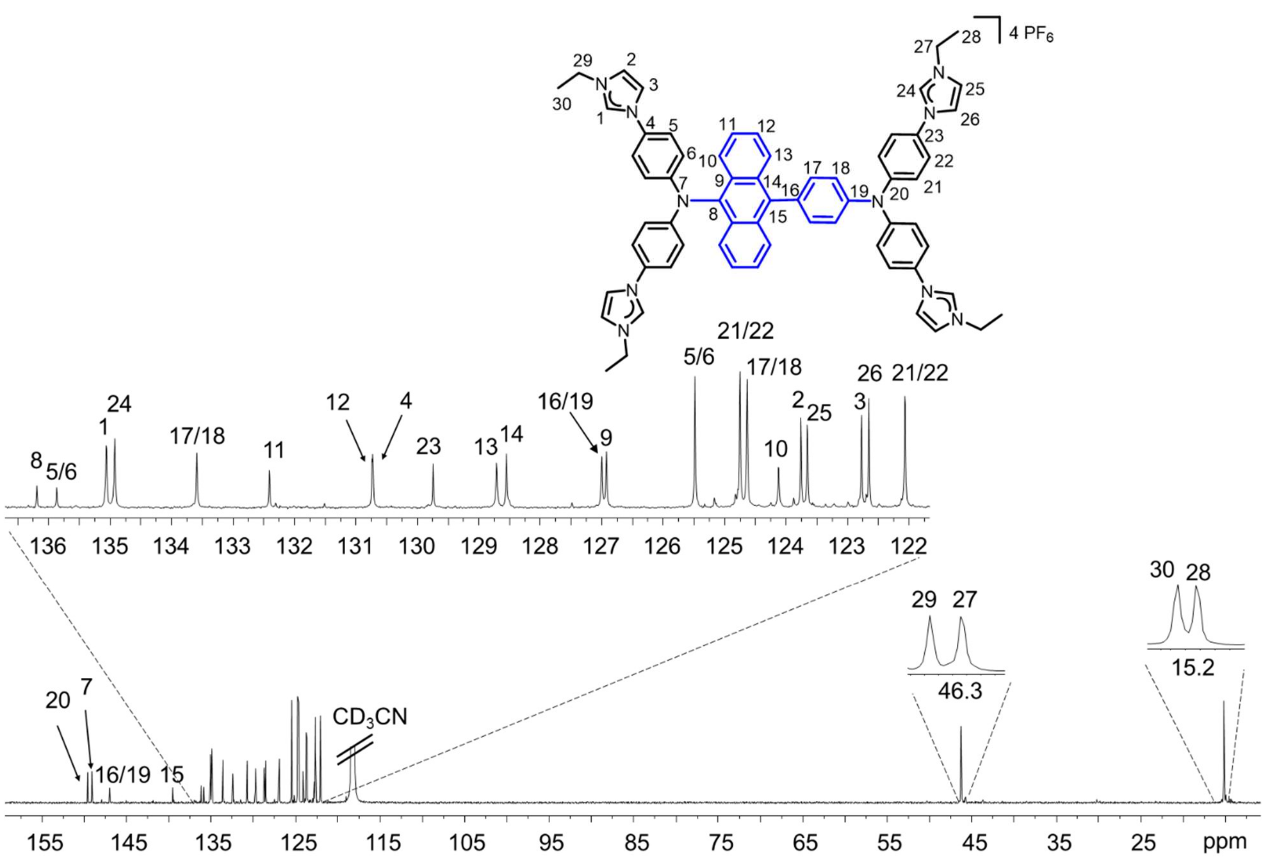

Figure S26. $^{13}C\{^{1}H\}$ NMR spectrum of H_4-**1c**$(PF_6)_4$ (100 MHz, CD_3CN, 298 K).

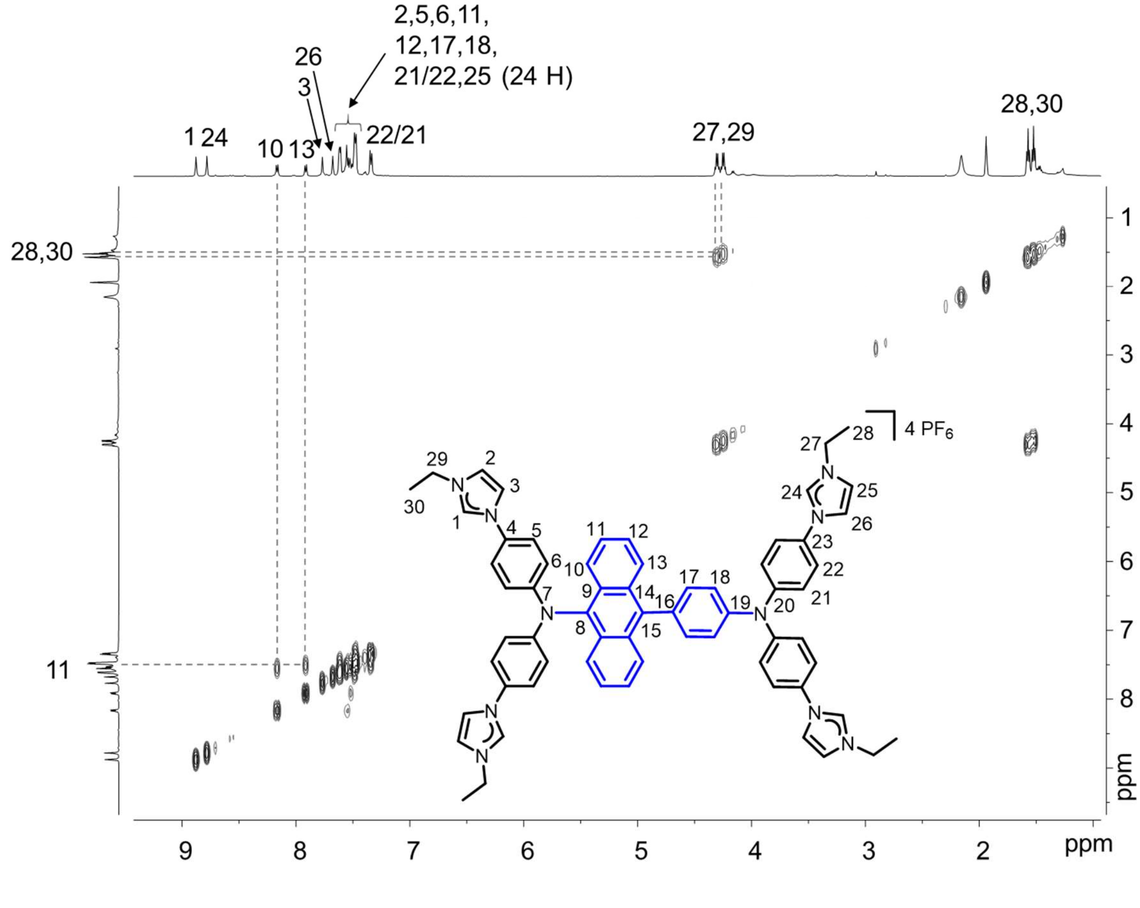

Figure S27. ^{1}H-^{1}H COSY spectrum of H_4-**1c**$(PF_6)_4$ (400 MHz, CD_3CN, 298 K).

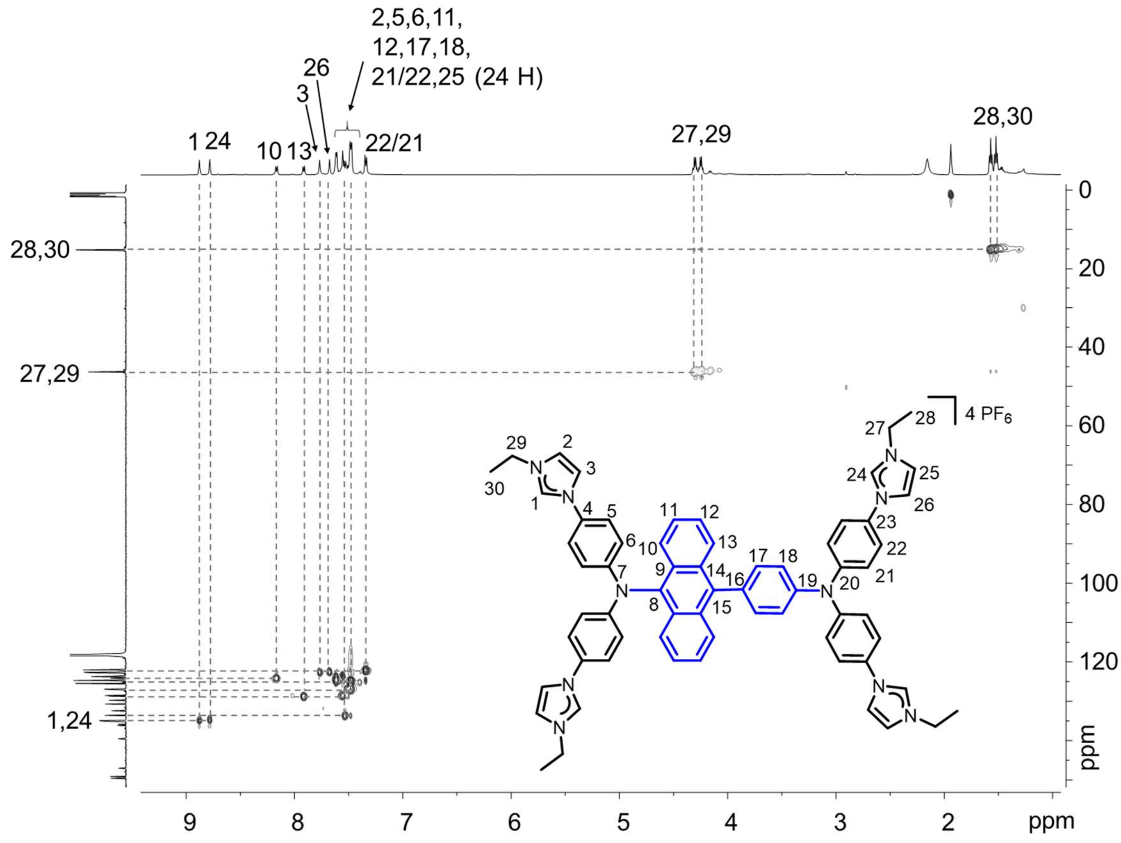

Figure S28. ^{1}H-^{13}C HSQC spectrum of H_4-**1c**$(PF_6)_4$ (400 MHz, CD_3CN, 298 K).

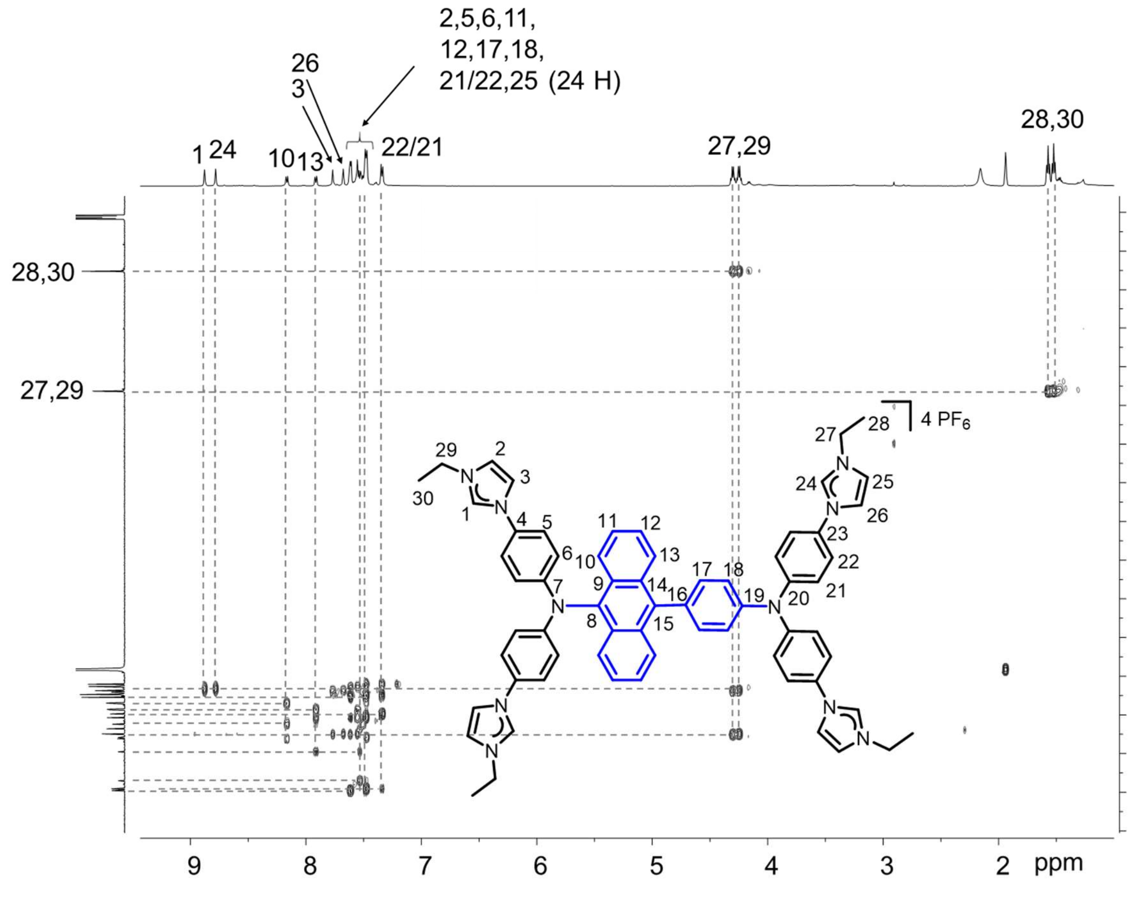

Figure S29. ^{1}H-^{13}C HMBC spectrum of H_4-**1c**$(PF_6)_4$ (400 MHz, CD_3CN, 298 K).

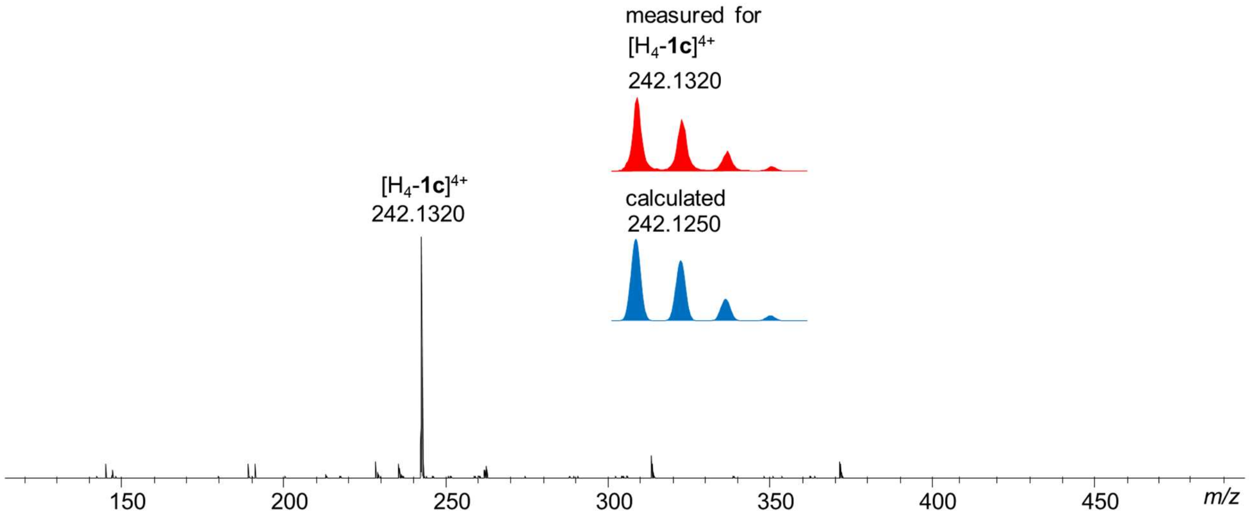

Figure S30. ESI mass spectrum (positive ions) of H_4-**1c**$(PF_6)_4$. Isotope distribution of a selected cation is shown as inset (experimentally measured distribution on top and calculated distribution at the bottom).

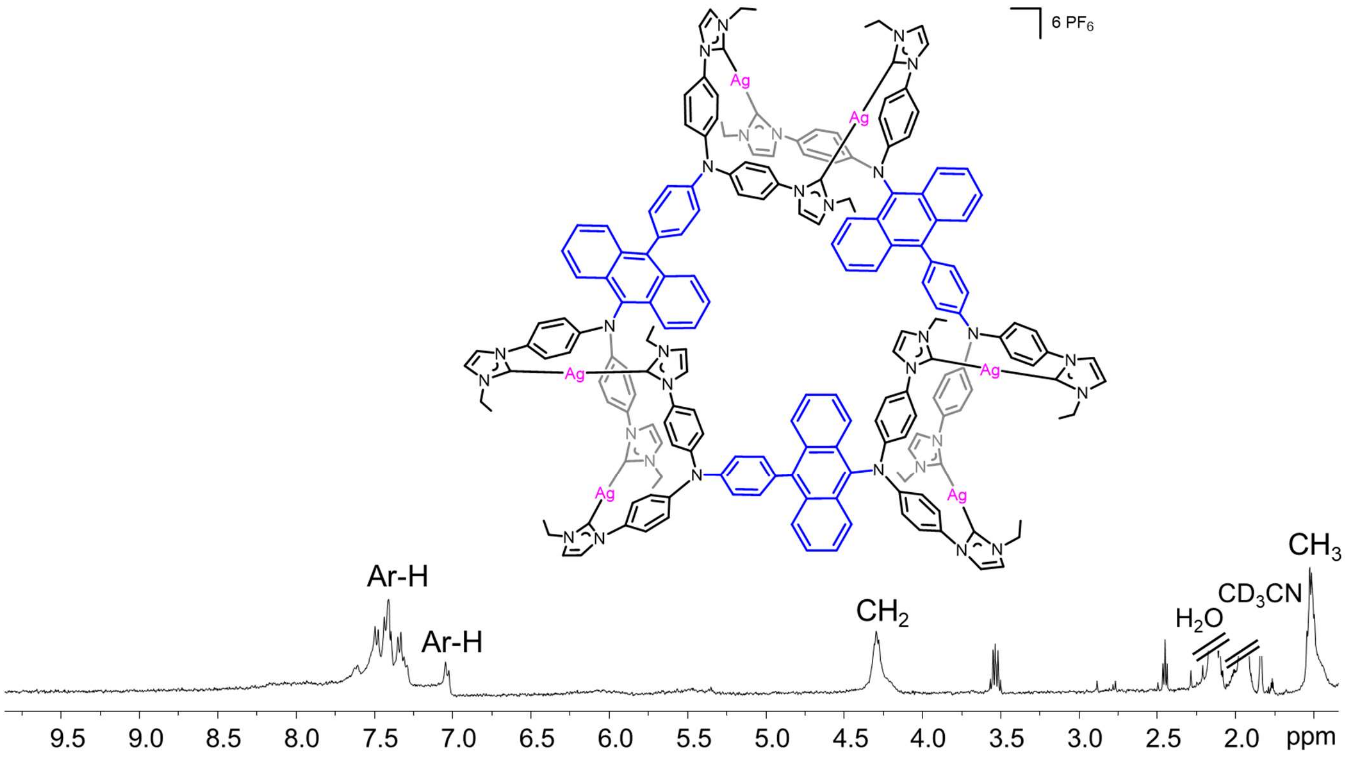

Figure S31. ^{1}H NMR spectrum of $[Ag_6(\mathbf{1c})_3](PF_6)_6$ (400 MHz, CD_3CN, 298 K).

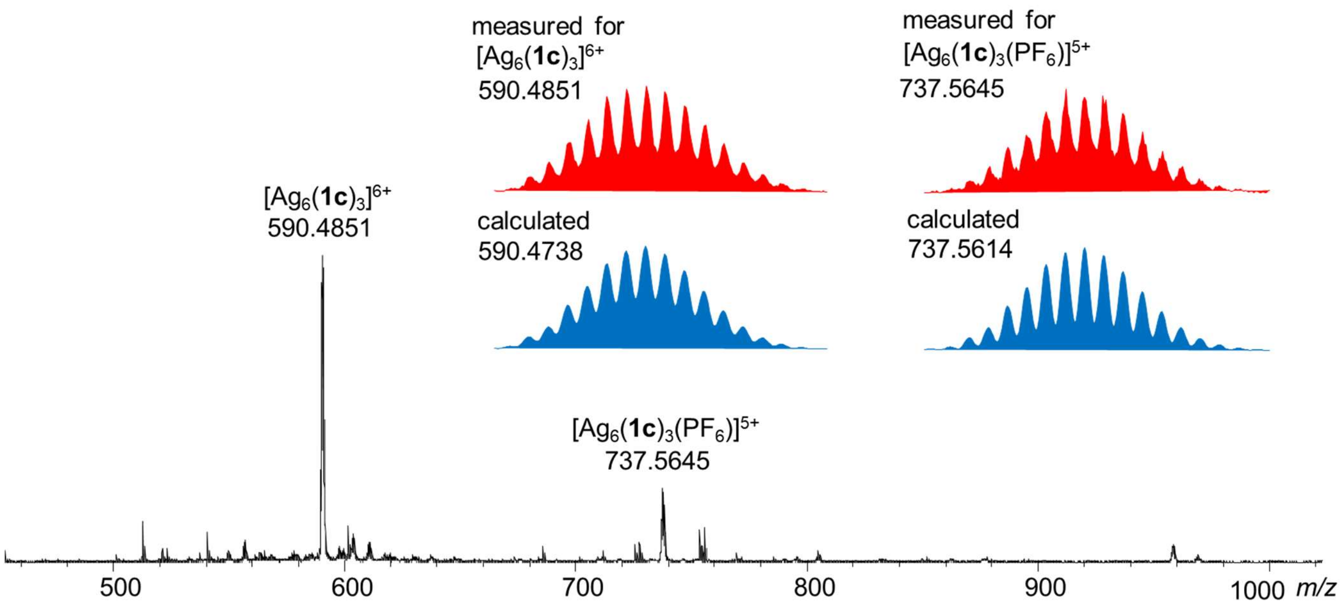

Figure S32. ESI mass spectrum (positive ions) of [Ag_6(**1c**)$_3$](PF_6)$_6$. Isotope distribution of selected cations are shown as inset (experimentally measured distribution on top and calculated distribution at the bottom).

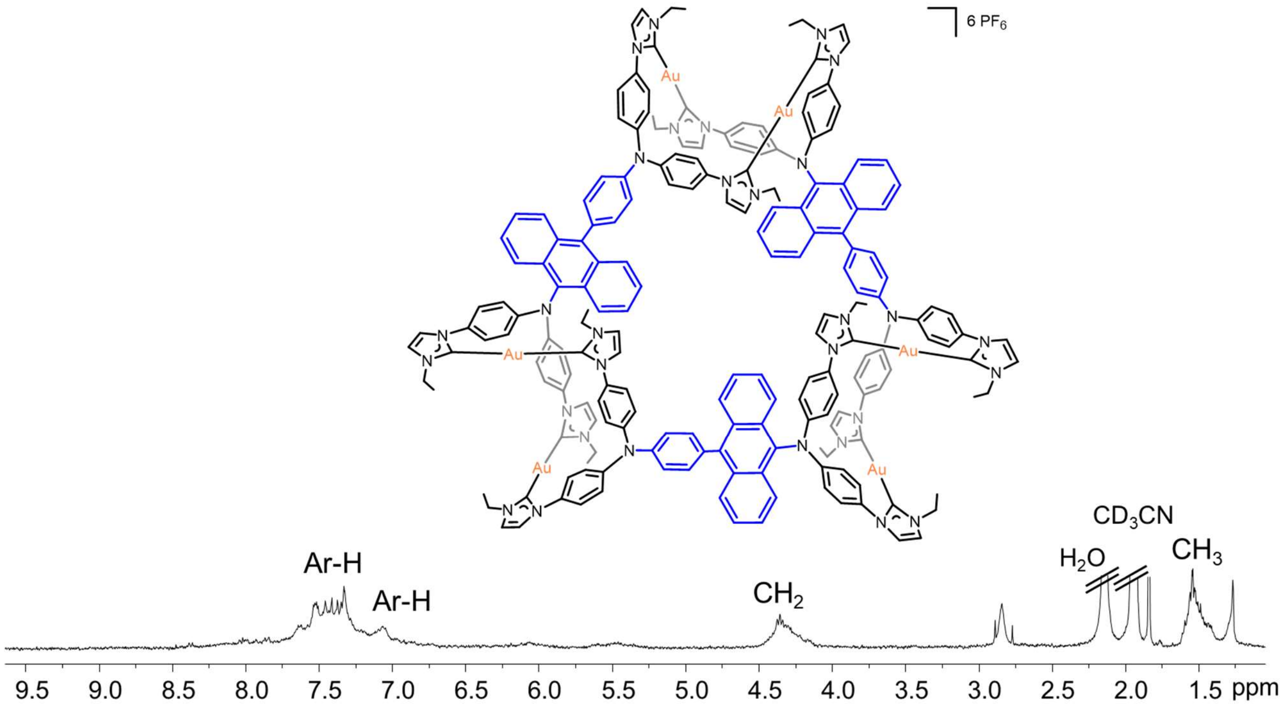

Figure S33. ^{1}H NMR spectrum of [Au_6(**1c**)$_3$](PF_6)$_6$ (400 MHz, CD_3CN, 298 K).

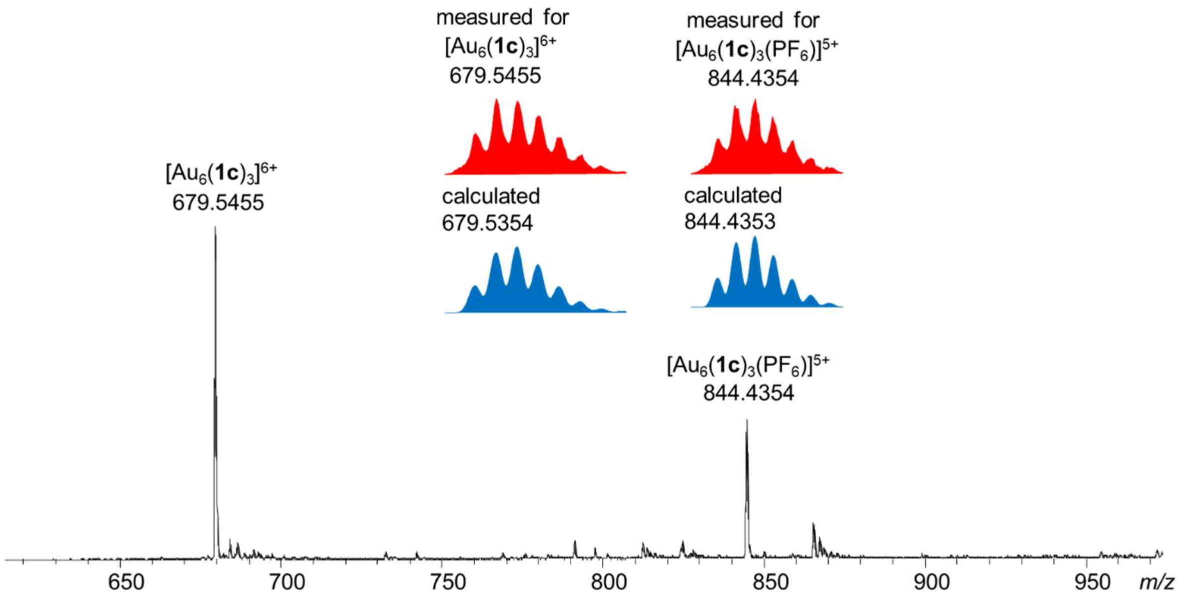

Figure S34. ESI mass spectrum (positive ions) of [Au_6(**1c**)$_3$](PF_6)$_6$. Isotope distribution of selected cations are shown as inset (experimentally measured distribution on top and calculated distribution at the bottom).

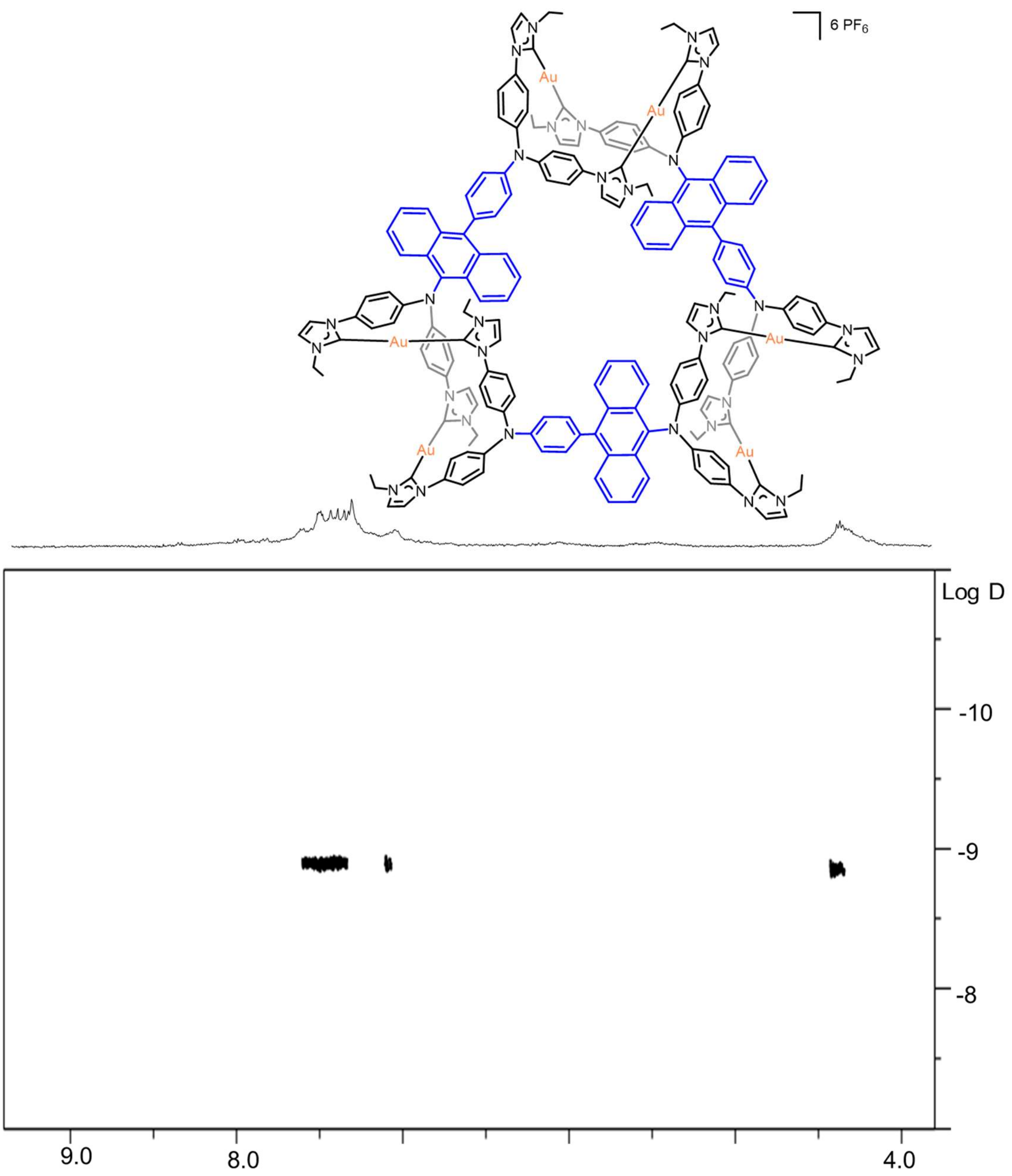

Figure S35. ^{1}H DOSY spectrum of $[Au_6(\mathbf{1c})_3](PF_6)_6$ (400 MHz, CD_3CN, 298 K).

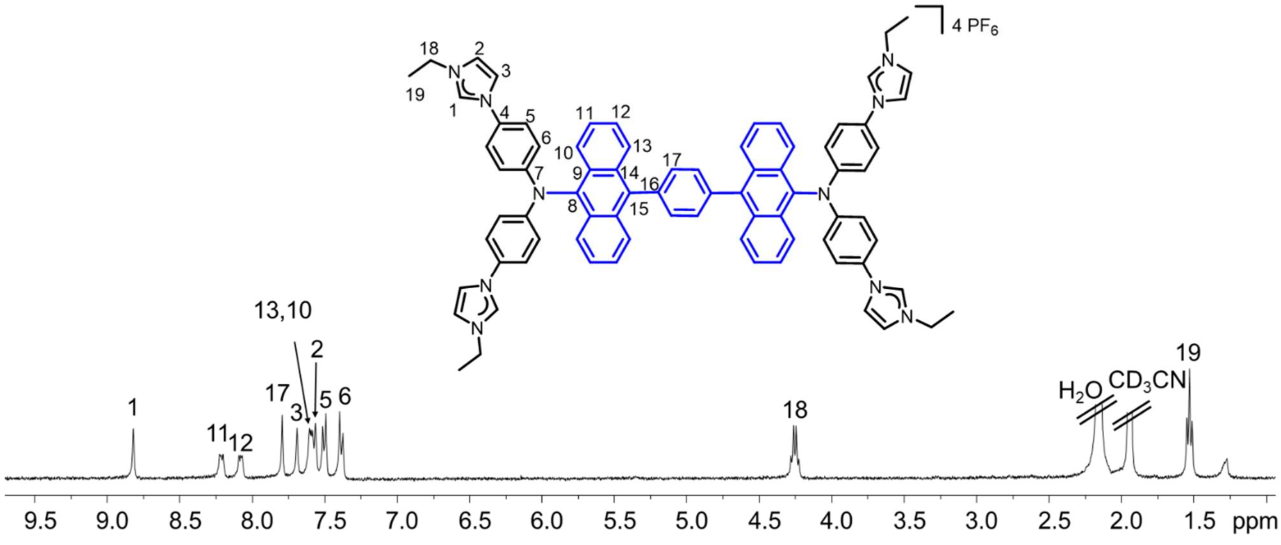

Figure S36. ^{1}H NMR spectrum of H_4-**1d**$(PF_6)_4$ (400 MHz, CD_3CN, 298 K).

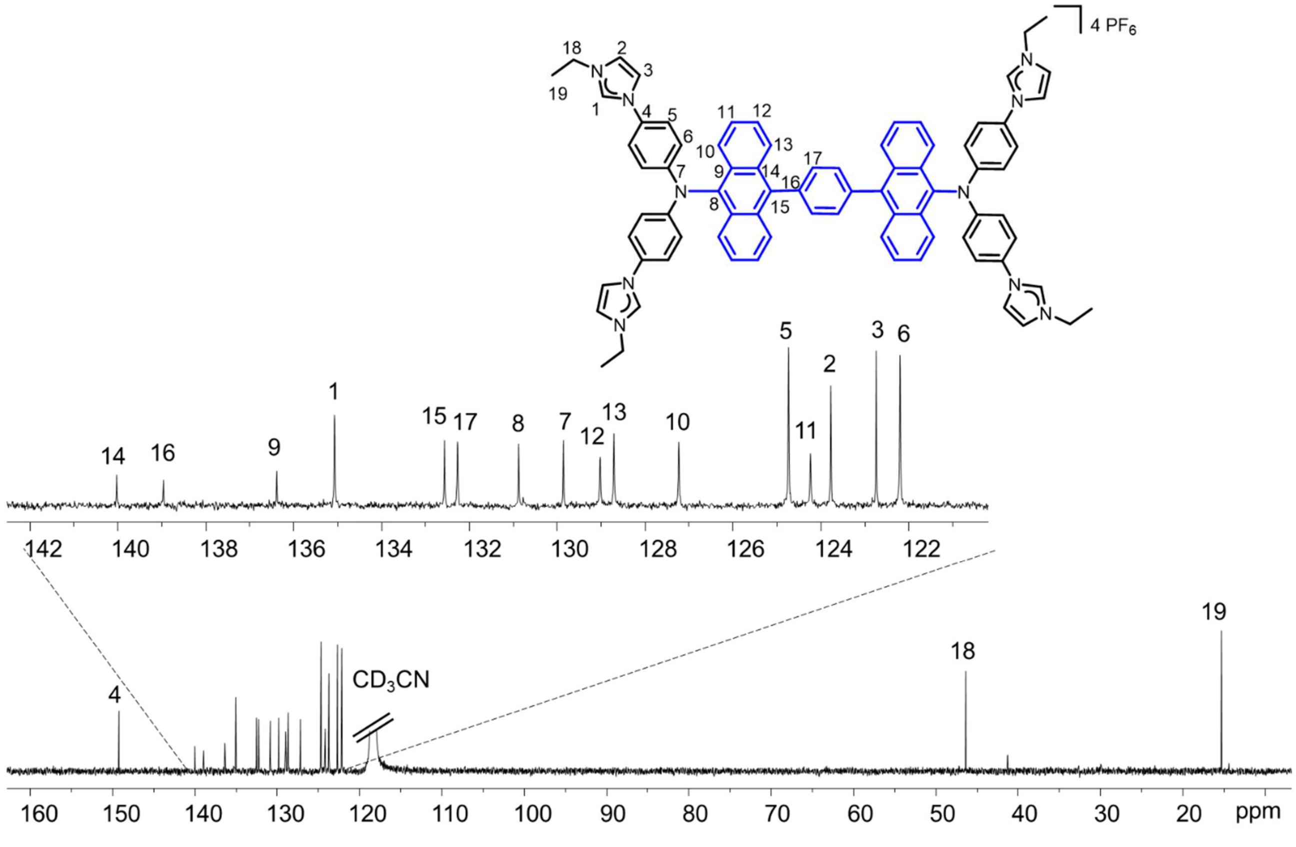

Figure S37. $^{13}C\{^{1}H\}$ NMR spectrum of H_4-**1d**$(PF_6)_4$ (100 MHz, CD_3CN, 298 K).

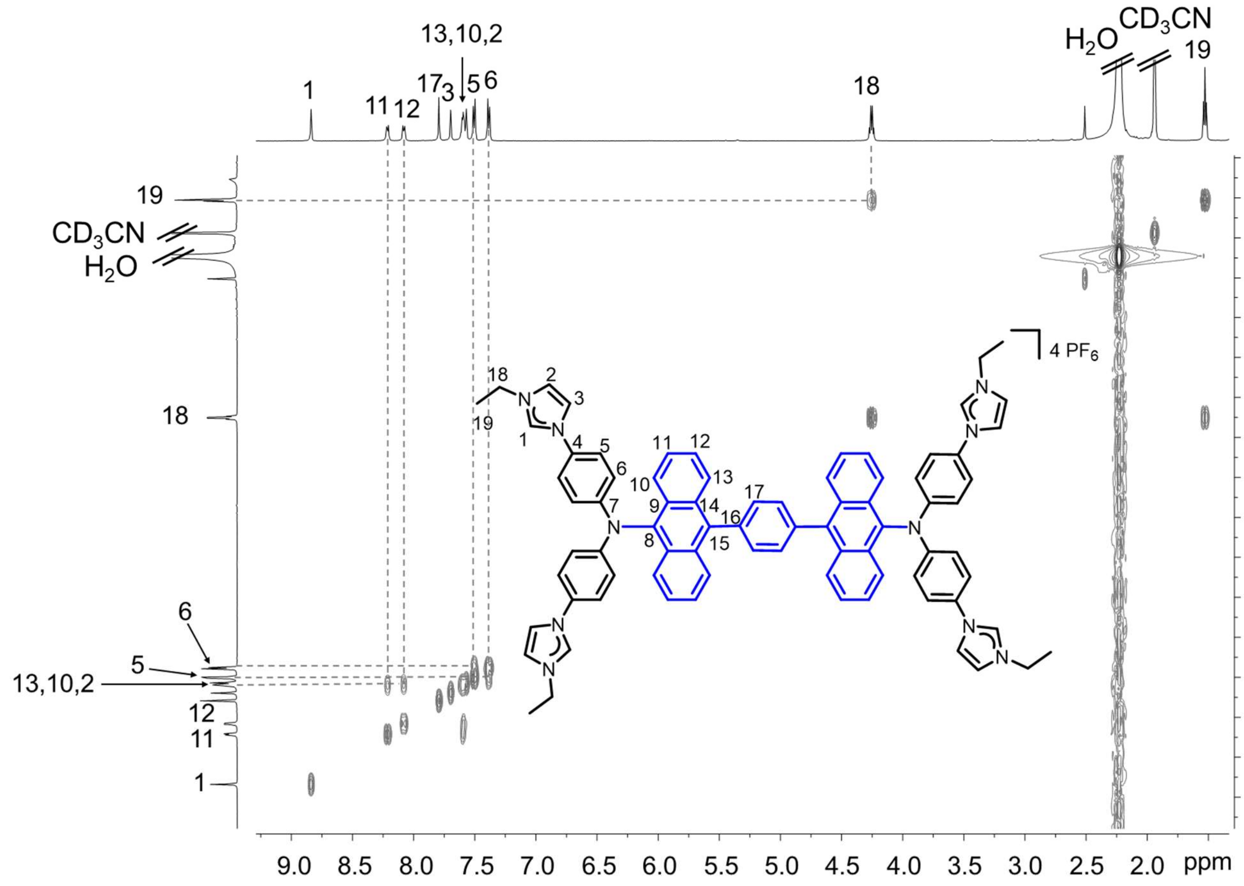

Figure S38. ^1H-^1H COSY spectrum of H_4-**1d**$(PF_6)_4$ (400 MHz, CD_3CN, 298 K).

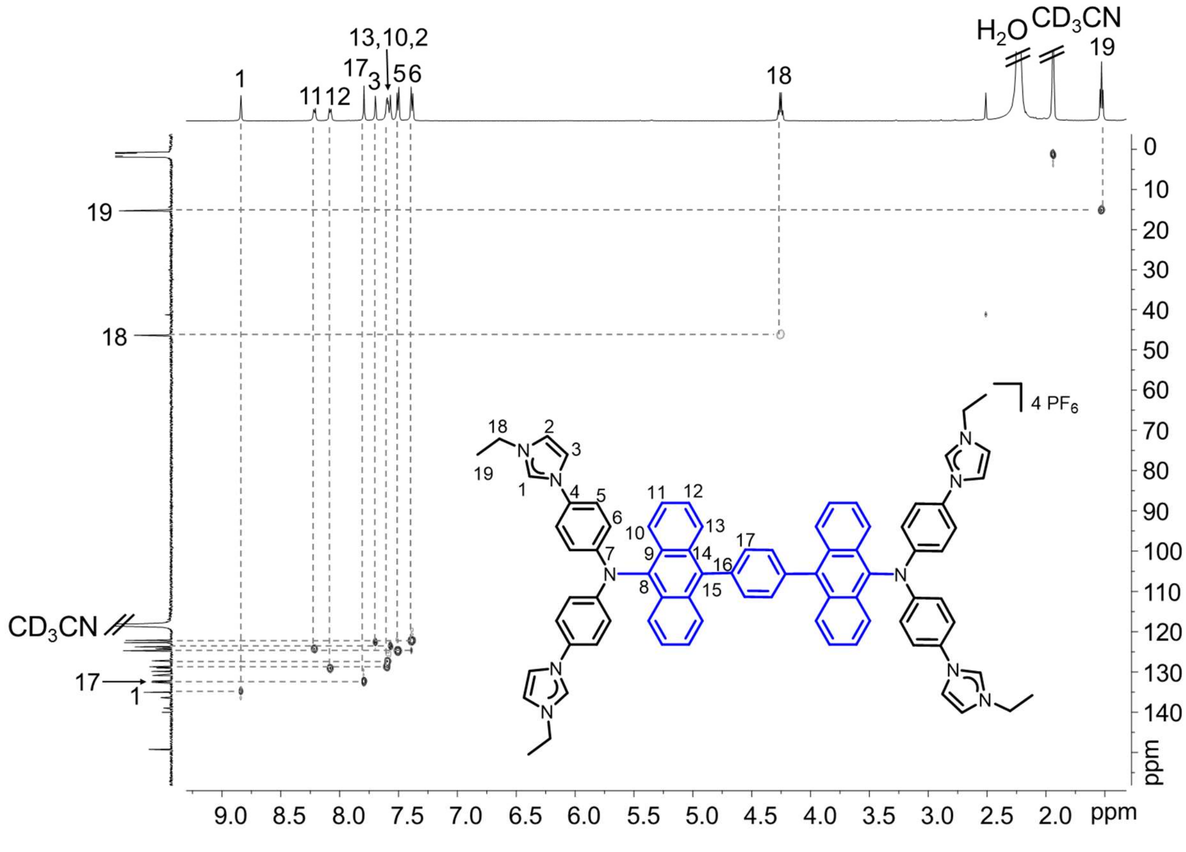

Figure S39. ^1H-^{13}C HSQC spectrum of H_4-**1d**$(PF_6)_4$ (400 MHz, CD_3CN, 298 K).

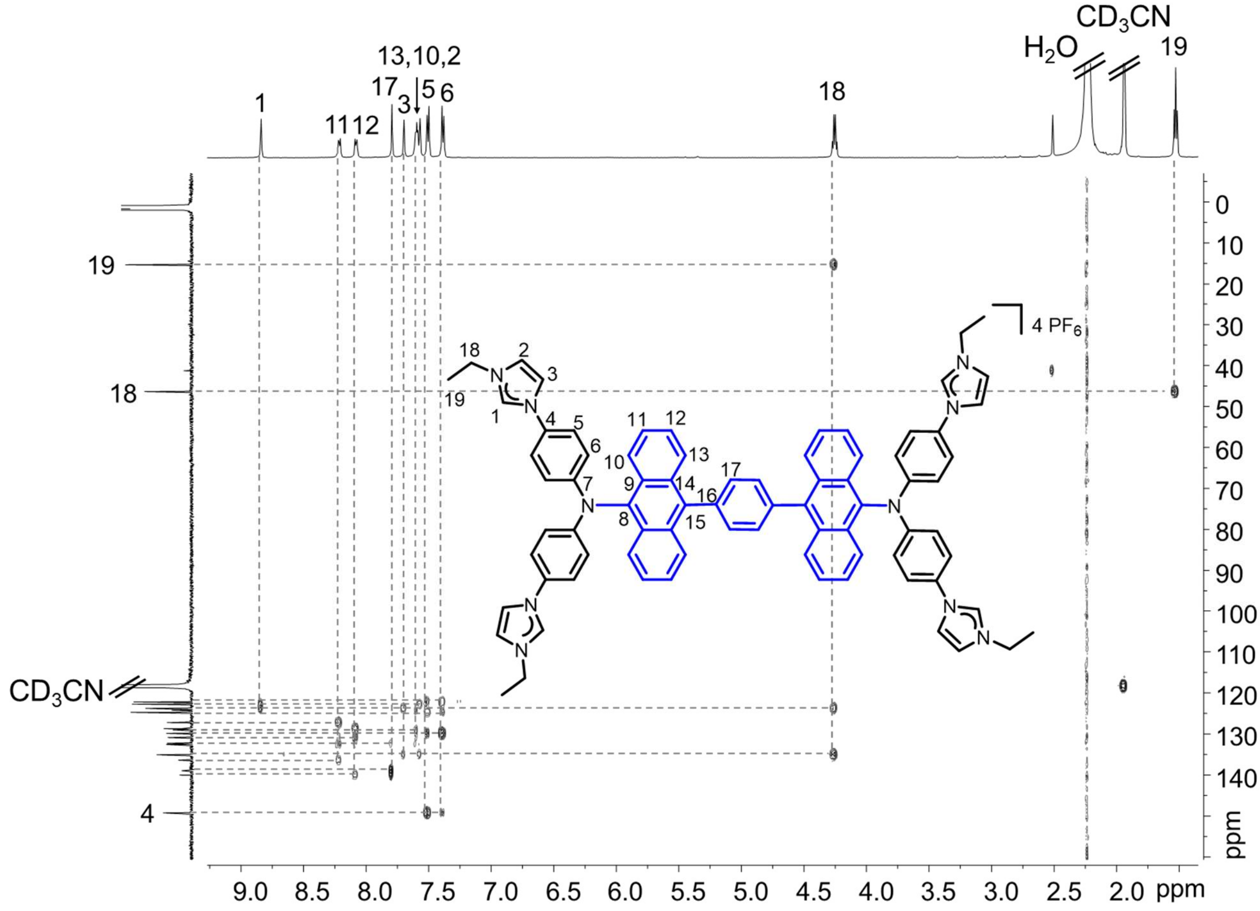

Figure S40. ^{1}H-^{13}C HMBC spectrum of H_4-**1d**$(PF_6)_4$ (400 MHz, CD_3CN, 298 K).

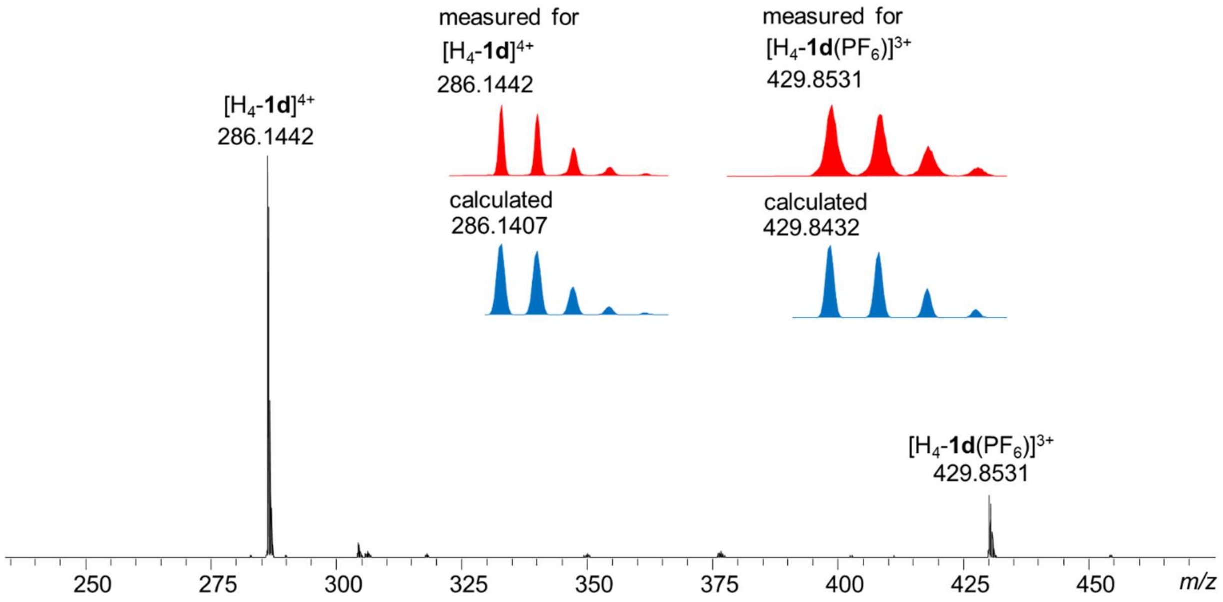

Figure S41. ESI mass spectrum (positive ions) of H_4-**1d**$(PF_6)_4$. Isotope distribution of selected cations are shown as inset (experimentally measured distribution on top and calculated distribution at the bottom).

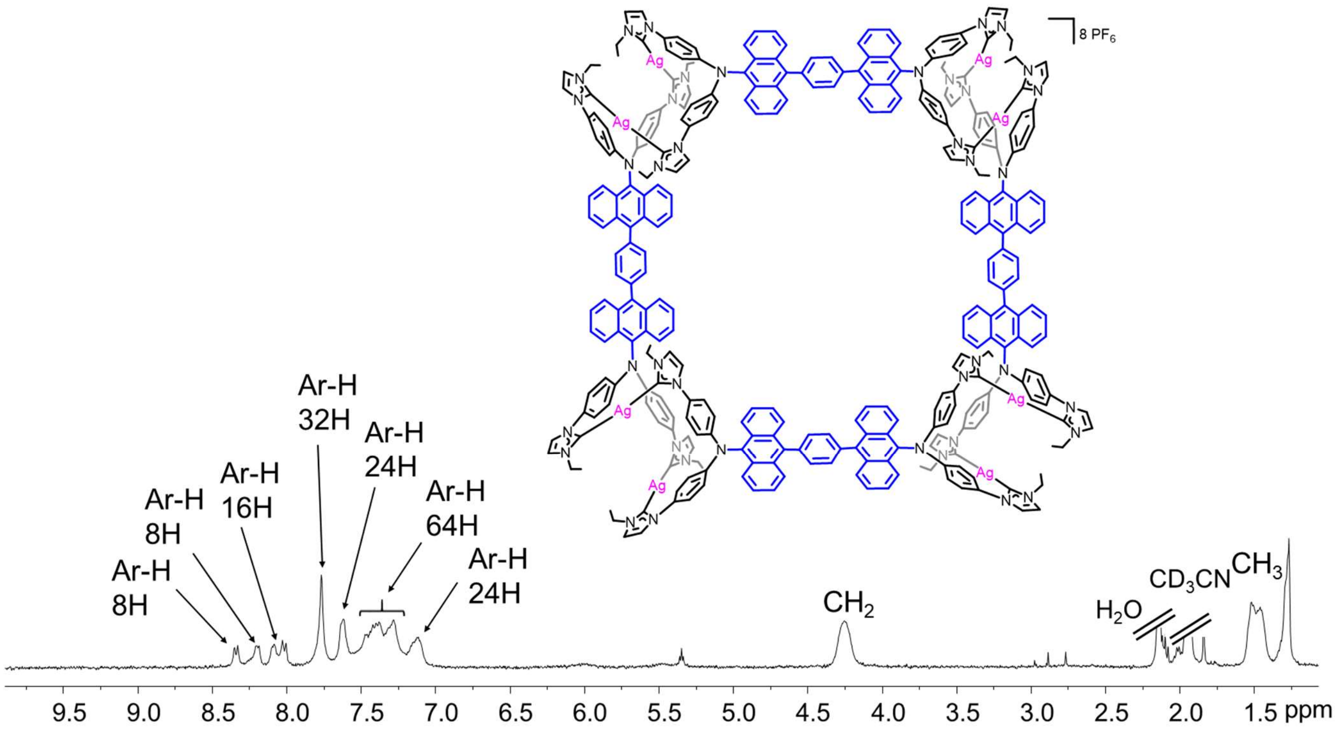

Figure S42. ^{1}H NMR spectrum of $[Ag_8(\mathbf{1d})_4](PF_6)_8$ (400 MHz, CD_3CN, 298 K).

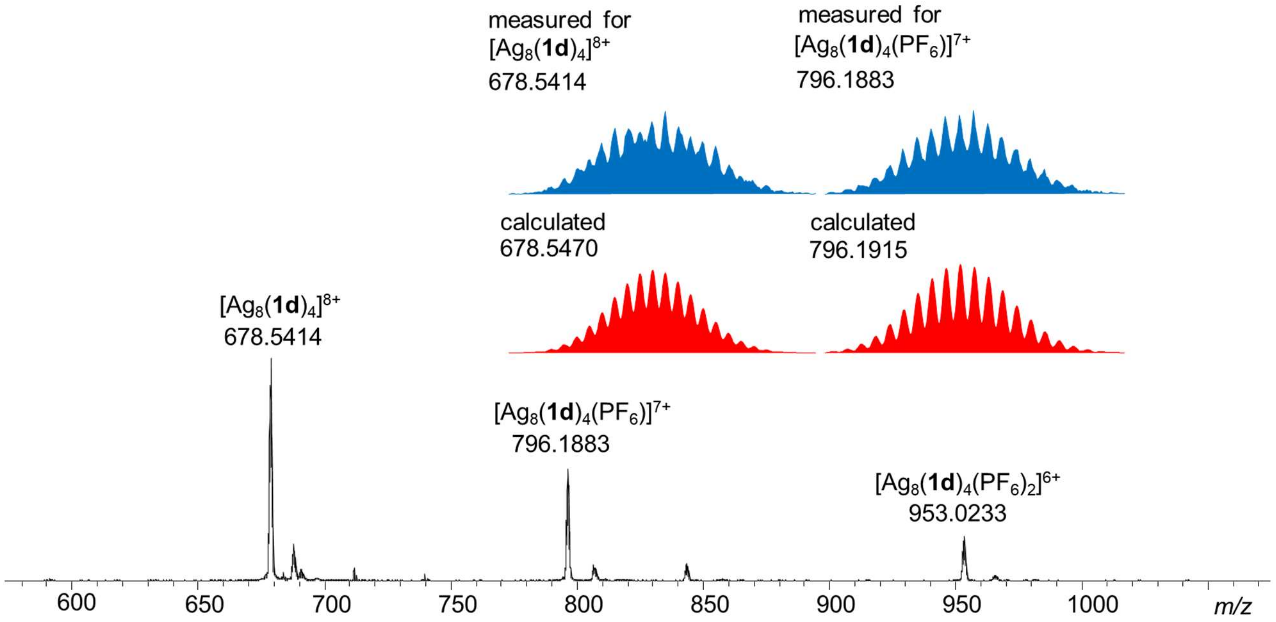

Figure S43. ESI mass spectrum (positive ions) of $[Ag_8(\mathbf{1d})_4](PF_6)_8$. Isotope distribution of selected cations are shown as inset (experimentally measured distribution on top and calculated distribution at the bottom).

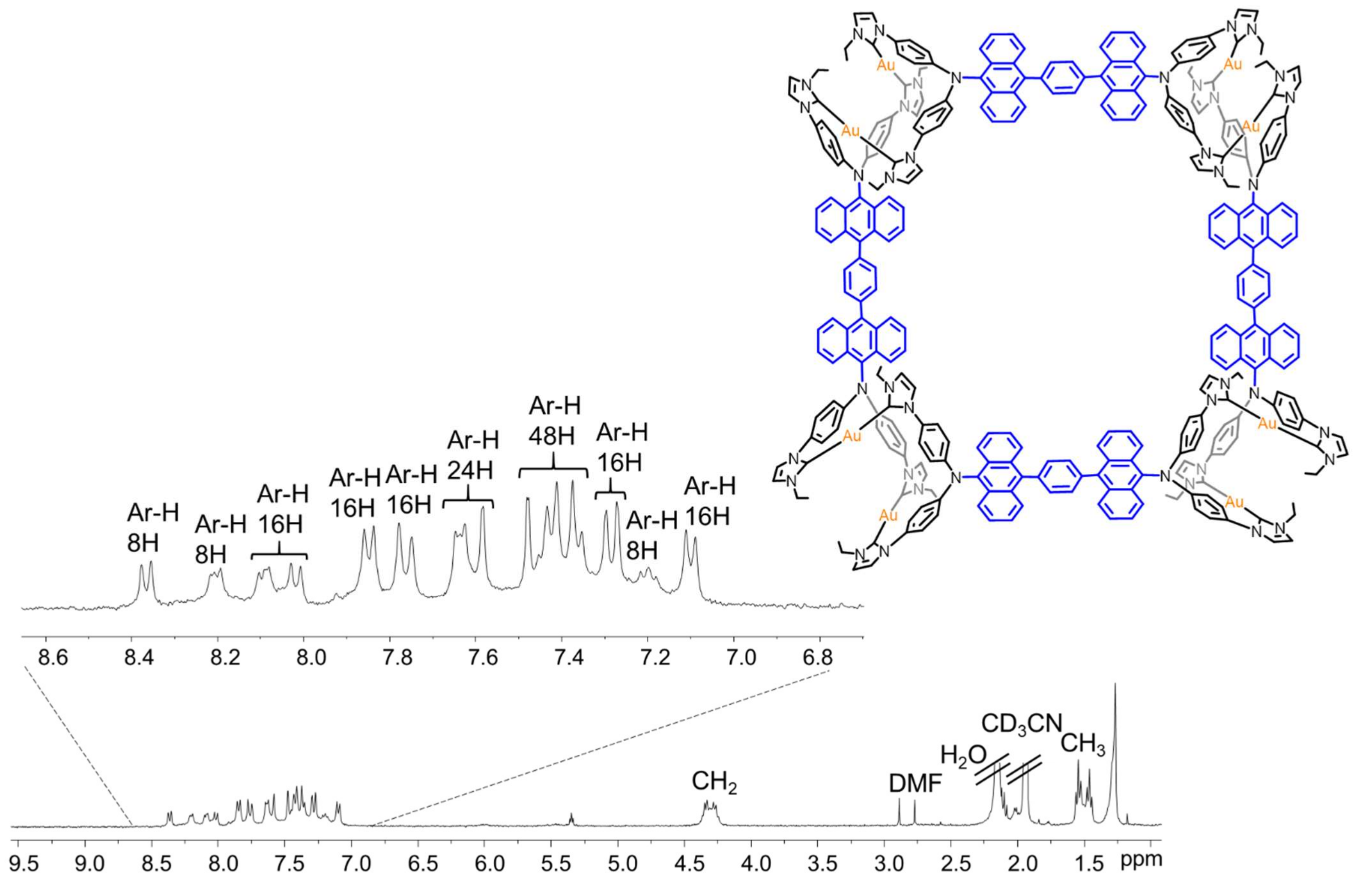

Figure S44. ^{1}H NMR spectrum of $[Au_8(\mathbf{1d})_4](PF_6)_8$ (400 MHz, CD_3CN, 298 K).

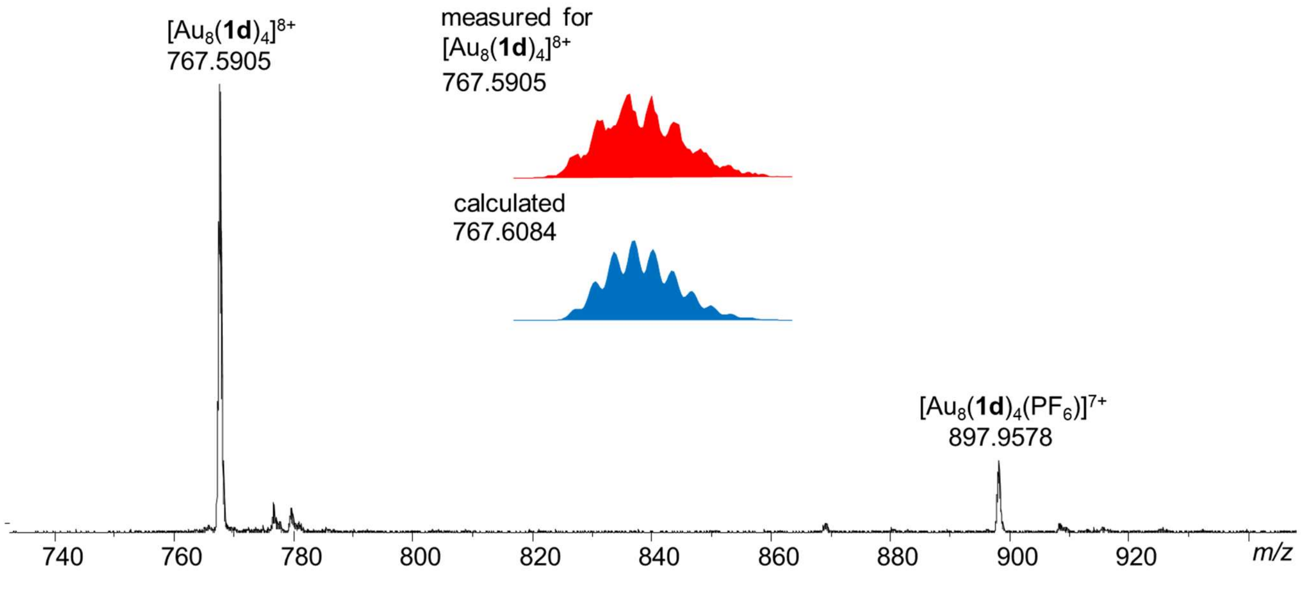

Figure S45. ESI mass spectrum (positive ions) of $[Au_8(\mathbf{1d})_4](PF_6)_8$. Isotope distribution of a selected cation is shown as inset (experimentally measured distribution on top and calculated distribution at the bottom).

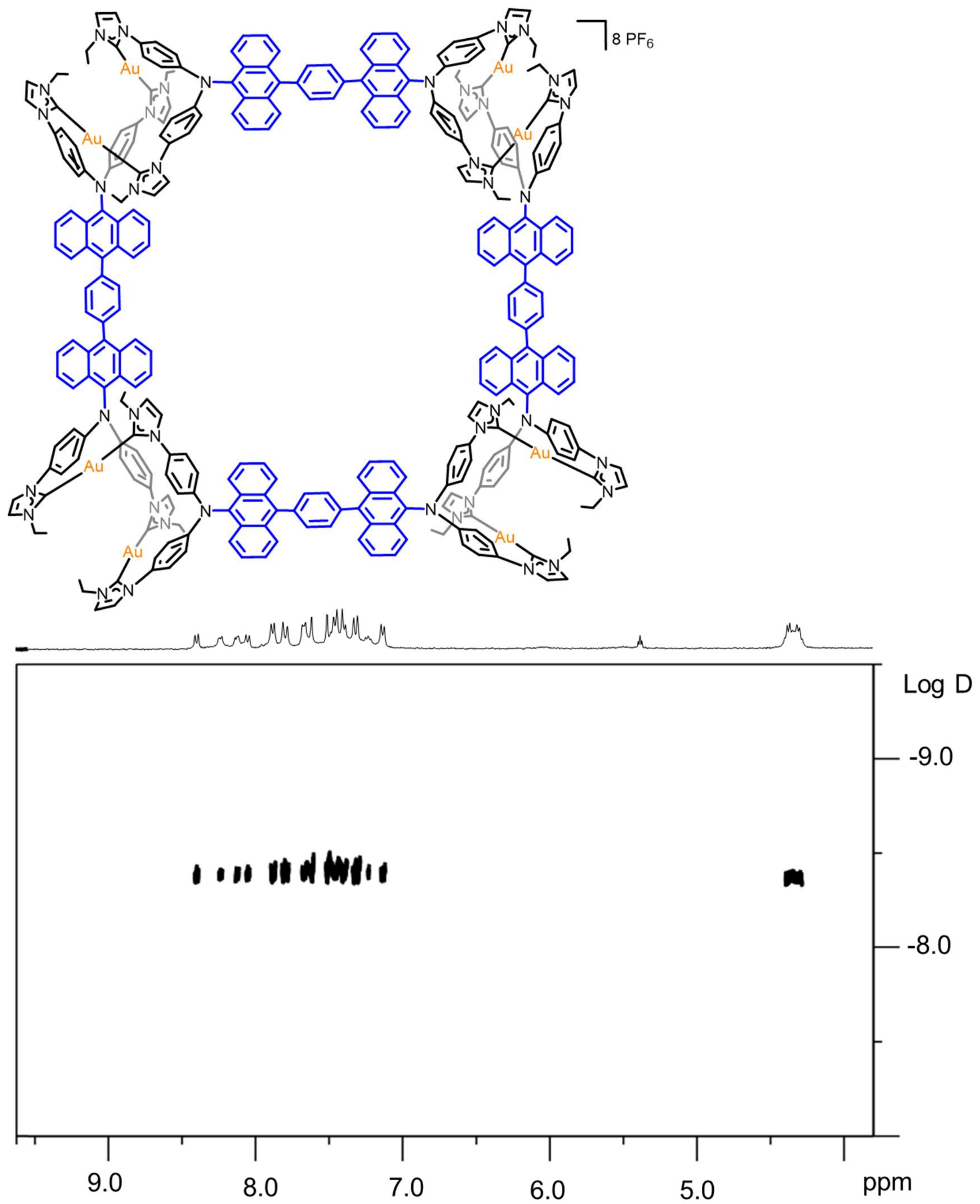

Figure S46. ^{1}H DOSY spectrum of $[Au_8(\mathbf{1d})_4](PF_6)_8$ (400 MHz, CD_3CN, 298 K).

7. X-ray crystallography

Single crystals of the ligand precursors H_4-**1a**$(PF_6)_4{\cdot}8DMSO$, H_4-**1b**$(PF_6)_4$, H_4-**1c**$(PF_6)_4{\cdot}2CH_3CN$, H_4-**1d**$(PF_6)_4{\cdot}4CH_3CN$ and of the Au-NHC complexes $[Au_8($**1a**$)_4](PF_6)_8{\cdot}6Et_2O{\cdot}2CH_3CN$, $[Au_4($**1b**$)_2](PF_6)_{2.8}(SbF_6)_{1.2}$, $[Au_6($**1c**$)_3](PF_6)_6$ and $[Au_8($**1d**$)_4](PF_6)_8$ were grown by slow diffusion of diethyl ether into acetonitrile solutions of the compounds at ambient temperature. In order to enhance the crystallinity of $[Au_4($**1b**$)_2](PF_6)_4$, a small amount of $NaSbF_6$ was added to the acetonitrile solution of the compound. This procedure led to the formation of single crystals which, however contained disordered PF_6^- and SbF_6^- anions. In a typical data collection, a suitable crystal was selected and mounted in inert oil or sealed in a glass tube with growth solution and transferred to the cold gas stream of a Bruker D8 Venture diffractometer with graphite-monochromated Mo Kα radiation (λ = 0.71073 Å). The data were corrected for absorption using the SADABS program. Structure solutions were found using the Olex2 program using Charge Flipping and were refined with the ShelXL refinement package using Least Squares minimization.[S2–S4] Hydrogen atoms were added to the structure models in calculated positions. Standard crystallographic methods were used to model disorder with constraints and restraints applied where necessary.

The value of the wR_2 factor is rather large for H_4-**1b**$(PF_6)_4$ (alert B level in checkCIF) due to the poor diffraction properties of the crystals. For crystals of $[Au_8($**1a**$)_4](PF_6)_8$ low bond precision on C–C bonds (alert B level in checkCIF) may be due to thermal motion in some areas of the rather large assembly. Both the anions and the tetracation in $[Au_4($**1b**$)_2](PF_6)_{2.8}(SbF_6)_{1.2}$ are severely disordered. While the structure of the tetranuclear tetracation could be established unambiguously from the data obtained, the disorder prevented a detailed discussing of metric parameters. For crystals of $[Au_6($**1c**$)_3](PF_6)_6$ the dataset was truncated at 1.1 Å. Nevertheless, the quality of the data is sufficient to show the connectivity within the compound. The alerts (both A and B level in checkCIF) arise from the poor diffraction properties of the crystals and weak high angle data.

Table S1. Crystal and refinement data for ligand precursor H_4-**1a**$(PF_6)_4$·8DMSO

Empirical formula	$C_{74}H_{104}N_{10}F_{24}O_8P_4S_8$
Formula weight	2098.03
Temperature, K	185.0
Crystal system	triclinic
Space group	$P\bar{1}$
a/Å	10.4174(11)
b/Å	14.9743(18)
c/Å	17.1486(18)
α/°	92.076(4)
β/°	105.717(3)
γ/°	109.527(3)
Volume/Å^3	2403.1(5)
Z	1
ρ_{calc} (g·cm^{-3})	1.450
μ (mm^{-1})	0.354
F (000)	1086
Crystal size/mm^3	0.17 × 0.15 × 0.14
Radiation	Mo Kα (λ = 0.71073)
2Θ range for data collection/°	4.98 to 50.91
Index ranges	$-12 \leq h \leq 12, -18 \leq k \leq 18, -20 \leq l \leq 20$
Reflections collected	41829
Independent reflections	8822 [R_{int} = 0.0550]
Data/restraints/parameters	8822/181/650
Goodness-of-fit on F^2	1.051
Final R indexes [$I \geq 2\sigma(I)$]	R_1 = 0.0918, wR_2 = 0.2399
Final R indexes [all data]	R_1 = 0.1322, wR_2 = 0.2755
Largest diff. peak/hole / e Å^{-3}	1.16/–0.79
CCDC	2168112

Table S2. Crystal and refinement data for complex $[Au_8(\mathbf{1a})_4](PF_6)_8·6Et_2O·2CH_3CN$

Empirical formula	$C_{260}H_{274}N_{42}Au_8F_{48}O_6P_8$
Formula weight	6818.69
Temperature/K	150.0
Crystal system	monoclinic
Space group	*C2/c*
a/Å	61.016(9)
b/Å	34.543(4)
c/Å	44.156(7)
α/°	90
β/°	130.912(7)
γ/°	90
Volume/Å^3	70331(18)
Z	8
ρ_{calc} (g·cm^{-3})	1.288
μ (mm^{-1})	3.434
F (000)	26880
Crystal size/mm^3	0.21 × 0.15 × 0.12
Radiation	Mo Kα (λ = 0.71073)
2Θ range for data collection/°	4.36 to 50.23
Index ranges	$-72 \leq h \leq 72$, $-34 \leq k \leq 41$, $-52 \leq l \leq 52$
Reflections collected	447165
Independent reflections	62307 [R_{int} = 0.0614]
Data/restraints/parameters	62307/1791/3306
Goodness-of-fit on F^2	1.059
Final *R* indexes [$I \geq 2\sigma(I)$]	R_1 = 0.0972, wR_2 = 0.2856
Final *R* indexes [all data]	R_1 = 0.1333, wR_2 = 0.3247
Largest diff. peak/hole / e Å^{-3}	4.70/–3.53
CCDC	2342093

Table S3. Crystal and refinement data for ligand precursor H_4-**1b**$(PF_6)_4$

Empirical formula	$C_{70}H_{64}N_{10}F_{24}N_{10}P_4$
Formula weight	1625.19
Temperature/K	210.0
Crystal system	monoclinic
Space group	*C2/c*
a/Å	22.956(7)
b/Å	20.697(7)
c/Å	18.076(5)
α/°	90
β/°	103.112(10)
γ/°	90
Volume/Å^3	8364(4)
Z	4
ρ_{calc} (g·cm^{-3})	1.291
μ (mm^{-1})	0.187
F (000)	3320
Crystal size/mm^3	0.21 × 0.09 × 0.09
Radiation	Mo K*α* (λ = 0.71073)
2Θ range for data collection/°	4.98 to 50.61
Index ranges	$-27 \le h \le 27$, $-24 \le k \le 24$, $-21 \le l \le 21$
Reflections collected	97260
Independent reflections	7389 [R_{int} = 0.0592]
Data/restraints/parameters	7389/268/498
Goodness-of-fit on F^2	1.679
Final *R* indexes [$I \ge 2\sigma(I)$]	R_1 = 0.1446, wR_2 = 0.3945
Final *R* indexes [all data]	R_1 = 0.1794, wR_2 = 0.4296
Largest diff. peak/hole / e Å^{-3}	1.48/–0.59
CCDC	2342095

Table S4. Crystal and refinement data for complex $[Au_4(\mathbf{1b})_2](PF_6)_{2.8}(SbF_6)_{1.2}$

Empirical formula	$C_{140}H_{120}N_{20}Au_4F_{24}P_{2.8}Sb_{1.2}$
Formula weight	3559.21
Temperature/K	210.0
Crystal system	monoclinic
Space group	*P*2/*m*
a/Å	19.3918(10)
b/Å	12.7720(8)
c/Å	22.0005(13)
α/°	90
β/°	100.567(2)
γ/°	90
Volume/Å^3	5356.5(5)
Z	1
ρ_{calc} (g·cm^{-3})	1.103
μ (mm^{-1})	2.957
F (000)	1735
Crystal size/mm^3	0.18 × 0.16 × 0.15
Radiation	Mo Kα (λ = 0.71073)
2Θ range for data collection/°	3.97 to 50.76
Index ranges	$-23 \leq h \leq 23$, $-5 \leq k \leq 15$, $-26 \leq l \leq 25$
Reflections collected	37374
Independent reflections	10280 [R_{int} = 0.0722]
Data/restraints/parameters	10280/387/663
Goodness-of-fit on F^2	1.036
Final *R* indexes [$I \geq 2\sigma(I)$]	R_1 = 0.0662, wR_2 = 0.1955
Final *R* indexes [all data]	R_1 = 0.1066, wR_2 = 0.2220
Largest diff. peak/hole / e Å^{-3}	1.95/–1.35
CCDC	2342091

Table S5. Crystal and refinement data for ligand precursor H_4-**1c**$(PF_6)_4 \cdot 2CH_3CN$

Empirical formula	$C_{68}H_{66}N_{12}F_{24}P_4$
Formula weight	1631.20
Temperature/K	220.0
Crystal system	triclinic
Space group	$P\bar{1}$
a/Å	11.5208(9)
b/Å	14.9493(13)
c/Å	22.5781(19)
α/°	106.174(2)
β/°	96.725(2)
γ/°	91.439(2)
Volume/Å^3	3702.2(5)
Z	2
ρ_{calc} (g·cm^{-3})	1.463
μ (mm^{-1})	0.213
F (000)	1668
Crystal size/mm^3	0.24 × 0.12 × 0.11
Radiation	Mo Kα (λ = 0.71073)
2Θ range for data collection/°	3.82 to 50.70
Index ranges	$-13 \leq h \leq 13$, $-18 \leq k \leq 17$, $-27 \leq l \leq 27$
Reflections collected	46438
Independent reflections	13279 [R_{int} = 0.0672]
Data/restraints/parameters	13279/399/1014
Goodness-of-fit on F^2	1.185
Final R indexes [$I \geq 2\sigma(I)$]	R_1 = 0.1082, wR_2 = 0.2975
Final R indexes [all data]	R_1 = 0.1722, wR_2 = 0.3500
Largest diff. peak/hole / e Å^{-3}	0.98/–0.84
CCDC	2342089

Table S6. Crystal and refinement data for complex $[Au_6(\mathbf{1c})_3](PF_6)_6$

Empirical formula	$C_{192}H_{168}N_{30}Au_6F_{36}N_{30}P_6$
Formula weight	4984.18
Temperature/K	261.0
Crystal system	triclinic
Space group	$P\bar{1}$
a/Å	19.905(4)
b/Å	28.930(8)
c/Å	31.841(5)
α/°	103.820(9)
β/°	108.130(7)
γ/°	109.827(6)
Volume/Å^3	15128(6)
Z	2
ρ_{calc} (g·cm^{-3})	1.086
μ (mm^{-1})	2.991
F (000)	4838
Crystal size/mm^3	0.14 × 0.12 × 0.10
Radiation	Mo Kα (λ = 0.71073)
2Θ range for data collection/°	3.24 to 36.00
Index ranges	$-17 \leq h \leq 17$, $-25 \leq k \leq 25$, $-27 \leq l \leq 27$
Reflections collected	65022
Independent reflections	19746 [R_{int} = 0.0976]
Data/restraints/parameters	19746/6274/2224
Goodness-of-fit on F^2	1.158
Final R indexes [$I \geq 2\sigma(I)$]	R_1 = 0.0911, wR_2 = 0.2677
Final R indexes [all data]	R_1 = 0.1203, wR_2 = 0.3024
Largest diff. peak/hole / e Å^{-3}	2.48/–2.15
CCDC	2342092

Table S7. Crystal and refinement data for ligand precursor H_4-**1d**$(PF_6)_4 \cdot 4CH_3CN$

Empirical formula	$C_{86}H_{80}N_{14}F_{24}N_{14}P_4$
Formula weight	1889.52
Temperature/K	220.0
Crystal system	monoclinic
Space group	$P2_1/c$
a/Å	13.3469(8)
b/Å	13.9809(9)
c/Å	23.6211(14)
α/°	90
β/°	90.100(2)
γ/°	90
Volume/Å^3	4407.7(5)
Z	2
ρ_{calc} (g·cm^{-3})	1.424
μ (mm^{-1})	0.190
F (000)	1940
Crystal size/mm^3	0.25 × 0.19 × 0.05
Radiation	Mo Kα (λ = 0.71073)
2Θ range for data collection/°	3.39 to 50.74
Index ranges	$-16 \le h \le 16$, $-16 \le k \le 16$, $-27 \le l \le 28$
Reflections collected	41066
Independent reflections	8035 [R_{int} = 0.0693]
Data/restraints/parameters	8035/78/601
Goodness-of-fit on F^2	1.049
Final R indexes [$I \ge 2\sigma(I)$]	R_1 = 0.0784, wR_2 = 0.2252
Final R indexes [all data]	R_1 = 0.1198, wR_2 = 0.2663
Largest diff. peak/hole / e Å^{-3}	0.55/–0.51
CCDC	2342090

Table S8. Crystal and refinement data for complex $[Au_8(\mathbf{1d})_4](PF_6)_8$

Empirical formula	$C_{312}H_{256}N_{40}Au_8F_{48}P_8$
Formula weight	7301.04
Temperature/K	227.0
Crystal system	orthorhombic
Space group	*Cmcm*
a/Å	35.386(7)
b/Å	22.366(4)
c/Å	67.926(13)
α/°	90
β/°	90
γ/°	90
Volume/Å^3	53761(18)
Z	4
ρ_{calc} (g·cm^{-3})	0.902
μ (mm^{-1})	2.249
F (000)	14368
Crystal size/mm^3	0.16 × 0.14 × 0.11
Radiation	Mo Kα (λ = 0.71073)
2Θ range for data collection/°	3.60 to 50.13
Index ranges	$-42 \leq h \leq 36$, $-26 \leq k \leq 26$, $-80 \leq l \leq 54$
Reflections collected	100091
Independent reflections	24046 [R_{int} = 0.0934]
Data/restraints/parameters	24046/444/900
Goodness-of-fit on F^2	1.063
Final R indexes [$I \geq 2\sigma (I)$]	R_1 = 0.0814, wR_2 = 0.2296
Final R indexes [all data]	R_1 = 0.1335, wR_2 = 0.2530
Largest diff. peak/hole / e Å^{-3}	1.70/–1.53
CCDC	2342094

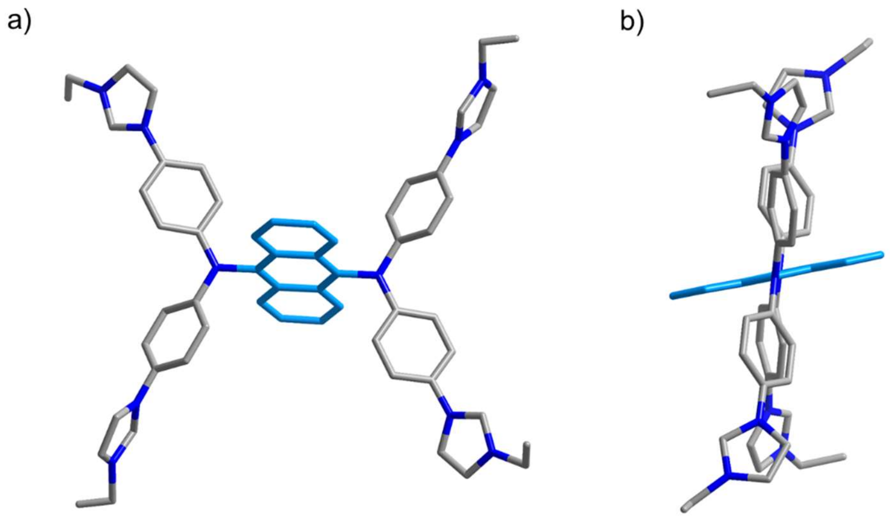

	C_{anth}– C_{anth}– C_{anth}– C_{anth}– C_{anth}– C_{anth}
C_{phenyl}–N–C_{phenyl}	72.26°

Figure S47. Two views of the molecular structure of the tetracation H_4-**1a**$^{4+}$ in H_4-**1a**$(PF_6)_4$·8DMSO. The cation H_4-**1a**$^{4+}$ resides on a crystallographic inversion center. Hydrogen atoms have been omitted for clarity.

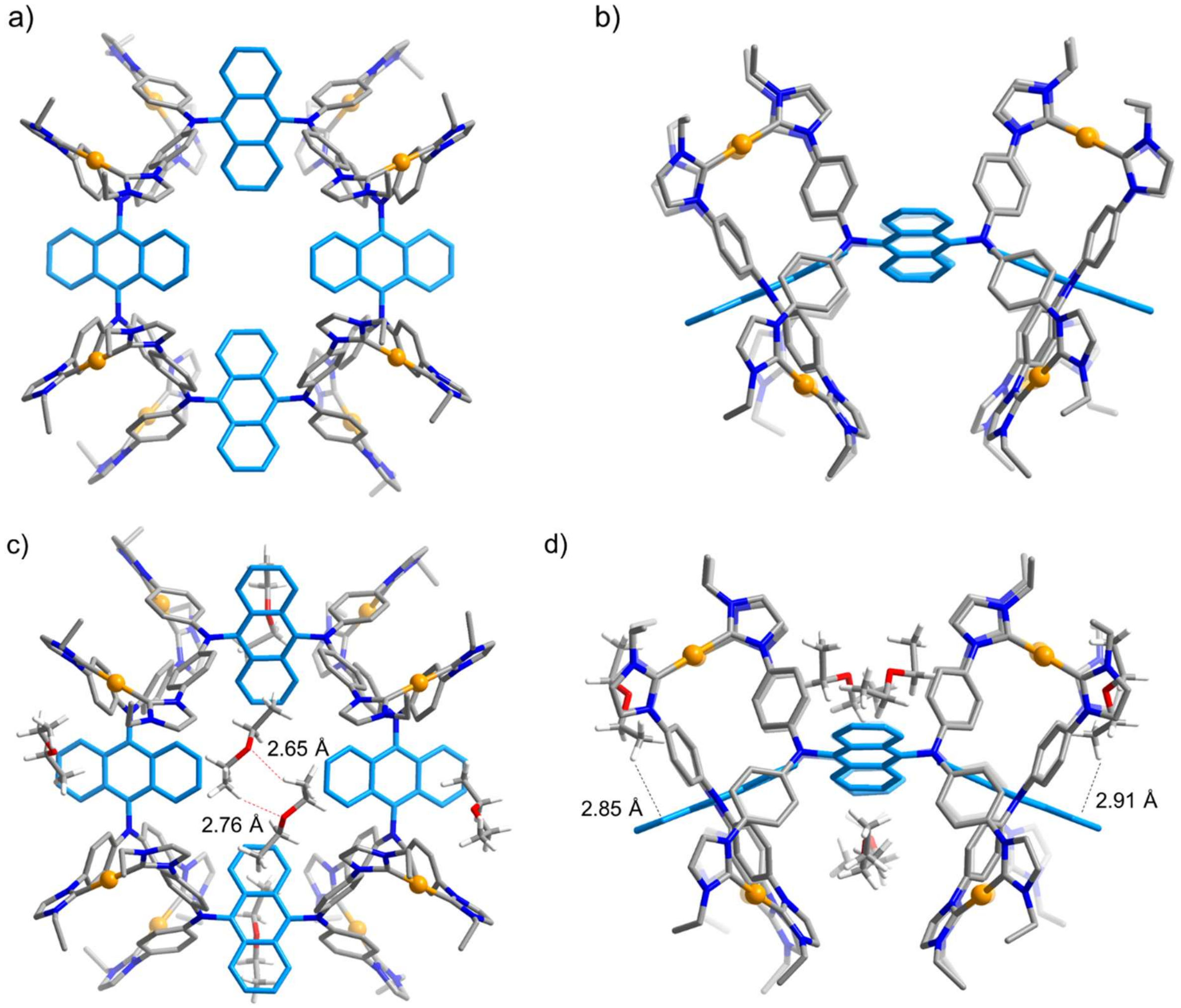

	C_{anth}– C_{anth}– C_{anth}– C_{anth}– C_{anth}– C_{anth}
C_{phenyl}–N–C_{phenyl}	76.62°, 78.38°
C_{phenyl}–N–C_{phenyl}	76.91°, 78.49°
C_{phenyl}–N–C_{phenyl}	77.42°, 77.95°
C_{phenyl}–N–C_{phenyl}	78.70°, 78.37

Figure S48. a and b) Two views of the complex octacation $[Au_8(\mathbf{1a})_4]^{8+}$ in $[Au_8(\mathbf{1a})_4](PF_6)_8{\cdot}6Et_2O{\cdot}2CH_3CN$. c) Depiction of the encapsulated diethyl ether molecules in $[Au_8(\mathbf{1a})_4]^{4+}$. D) Illustration of the intramolecular hydrogen π-bond interactions.

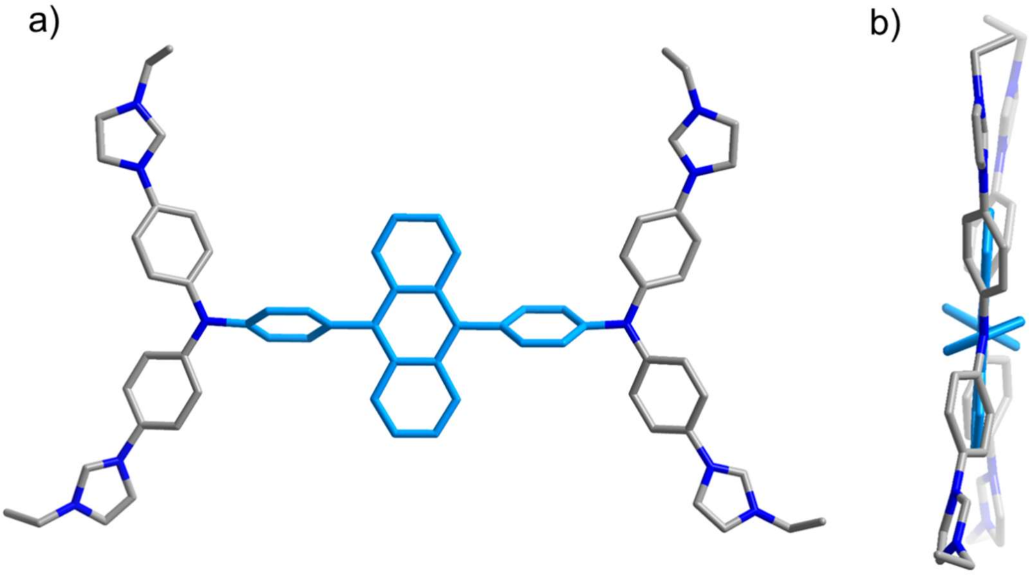

	C_{anth}– C_{anth}– C_{anth}– C_{anth}– C_{anth}– C_{anth}
C_{phenyl}–N–C_{phenyl}	10.29°

Figure S49. Two views of the molecular structure of the tetracation H_4-**1b**$^{4+}$ in H_4-**1b**$(PF_6)_4$.

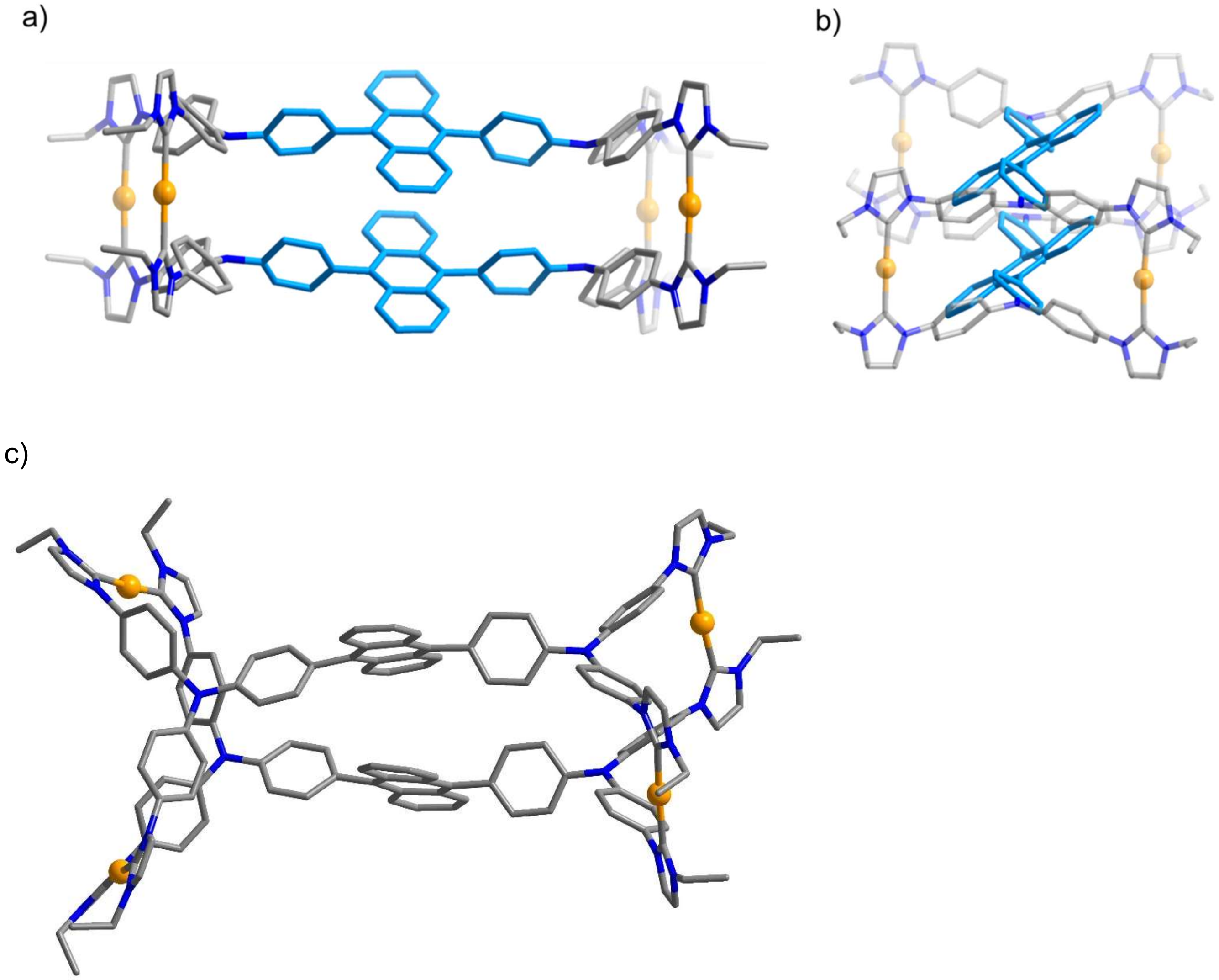

Figure S50. Depictions of the tetracation $[Au_4(\mathbf{1b})_2]^{4+}$ in $[Au_4(\mathbf{1b})_2](PF_6)_{2.8}(SbF_6)_{1.2}$.
a and b) Molecular structure obtained from low quality X-ray data (disorder of the anthracene and phenyl rings and the PF_6^- and SbF_6^- anions in the unit cell, the unit cell contains one cation residing on a special position with site symmetry 2/m), confirming the overall composition of the tetracation. Due to the disorder of the central anthracene ring, the angle between this ring and the N(phenylimidazol-2-ylidene)$_2$ C_{phenyl}–N–C_{phenyl} plane of the N(phenylimidazol-2-ylidene)$_2$ unit cannot be determined unequivocally. In the depicted molecule this angle measures 33.62°.
c) Results of the DFT calculations, confirming the formation of a tetranuclear assembly. Owing to the absence of packing effects and the anions, the angles between the planes of the anthracene rings and the N(phenylimidazol-2-ylidene)$_2$ units fall in the range of 44.54° to 93.79°. This variation confirms that the N(phenylimidazol-2-ylidene)$_2$ units can freely rotate relative to the anthrance ring which forms the basis for the formation of the tetranuclear assembly.

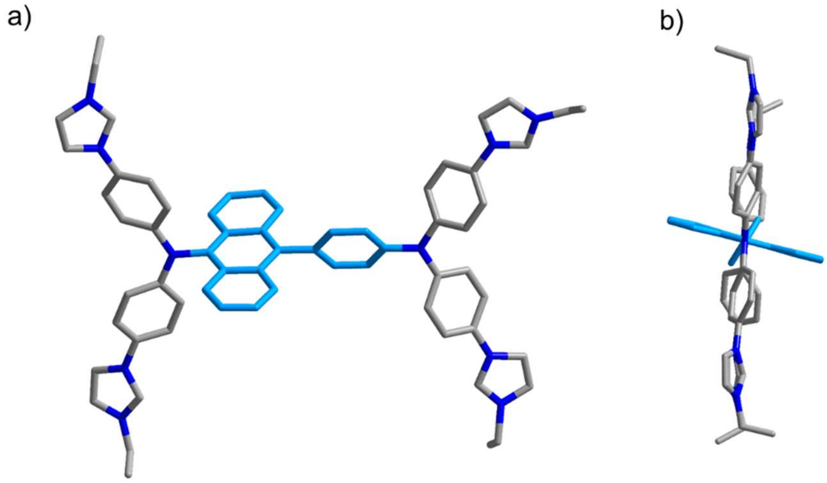

	C_{anth}– C_{anth}– C_{anth}– C_{anth}– C_{anth}– C_{anth}
C_{phenyl}–N–C_{phenyl} directly bound	72.72°
C_{phenyl}–N–C_{phenyl} phenyl bridge	77.57°

Figure S51. Two views of the molecular structure of the tetracation H_4-**1c**$^{4+}$ in H_4-**1c**$(PF_6)_4\cdot2CH_3CN$.

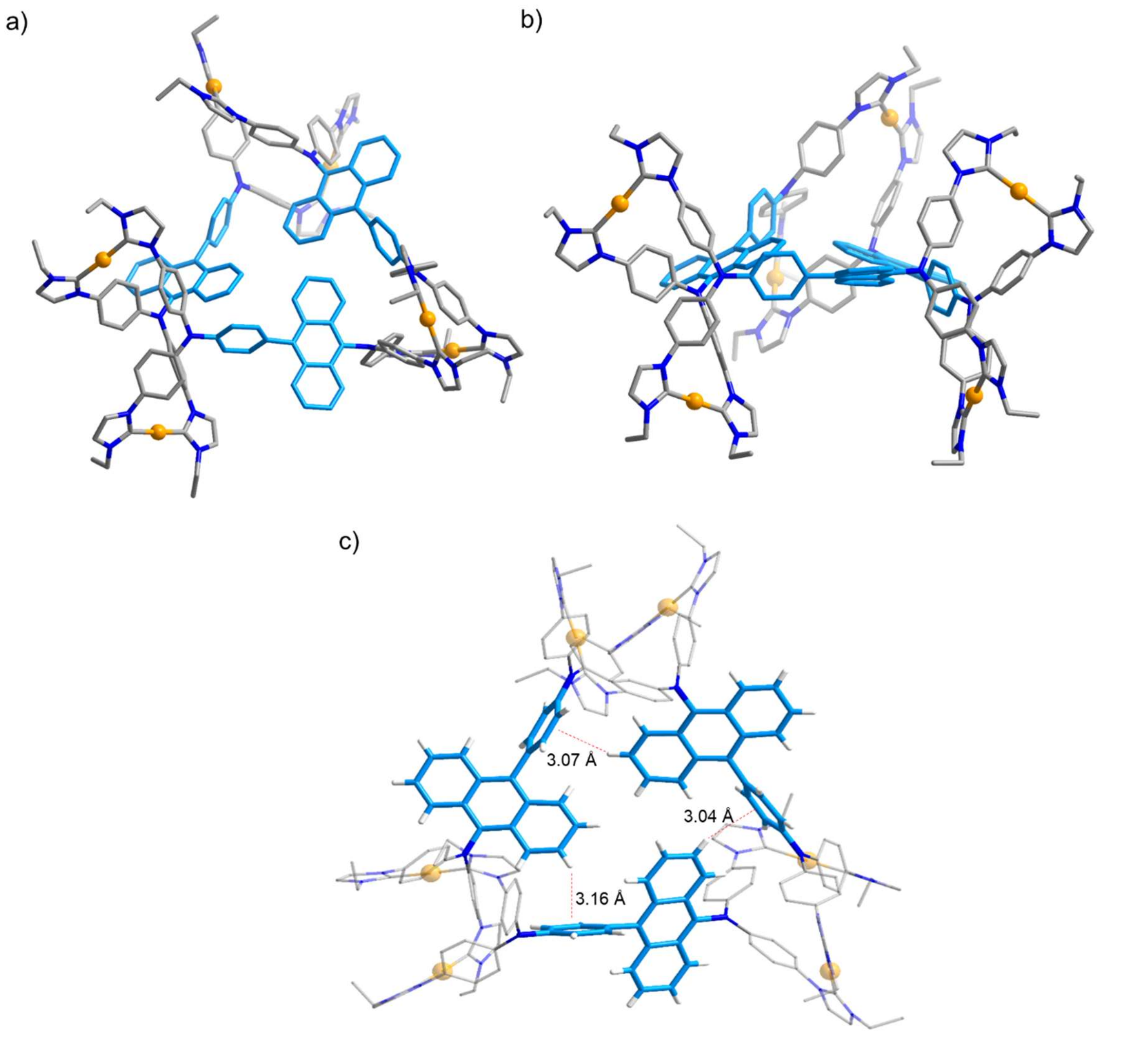

	C_{anth}– C_{anth}– C_{anth}– C_{anth}– C_{anth}– C_{anth}
C_{phenyl}–N–C_{phenyl} directly bound	74.09°, 73.29°, 73.13°
C_{phenyl}–N–C_{phenyl} phenyl bridge	89.54°, 81.99°, 29.78° please check

Figure S52. a and b) Two views of the complex hexacation $[Au_6(\mathbf{1c})_3]^{6+}$ in $[Au_6(\mathbf{1c})_3](PF_6)_6$. c) Illustration of the intramolecular hydrogen C−H···Ar π-bond interactions.

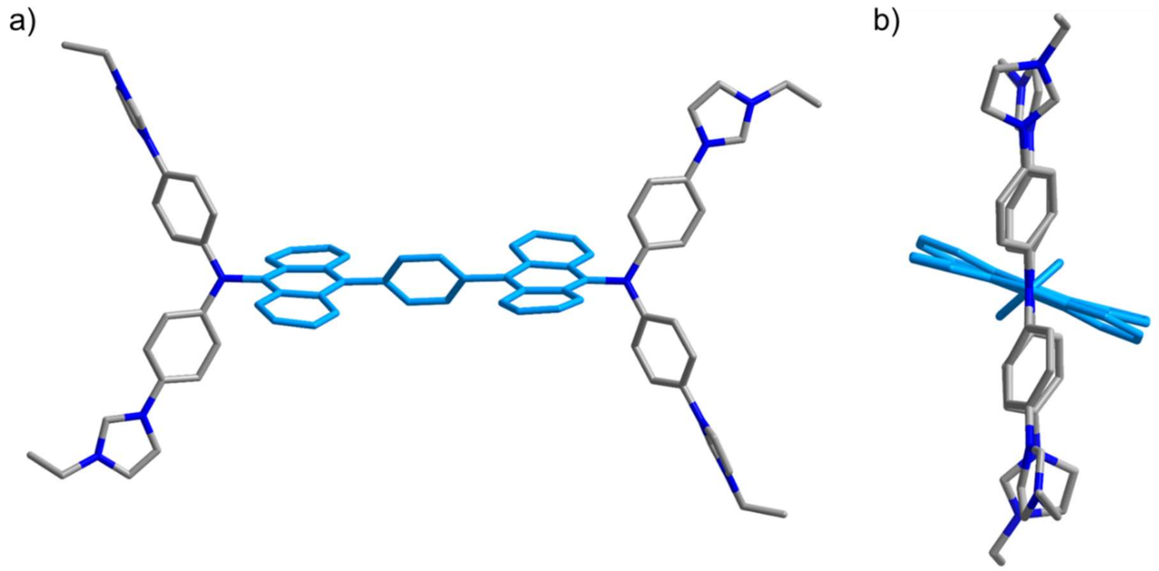

	C_{anth}– C_{anth}– C_{anth}– C_{anth}– C_{anth}– C_{anth}
C_{phenyl}–N–C_{phenyl}	69.64

Figure S53. a and b) Two views of the molecular structure of the tetracation H_4-**1d**$^{4+}$ in H_4-**1d**$(PF_6)_4 \cdot 4CH_3CN$.

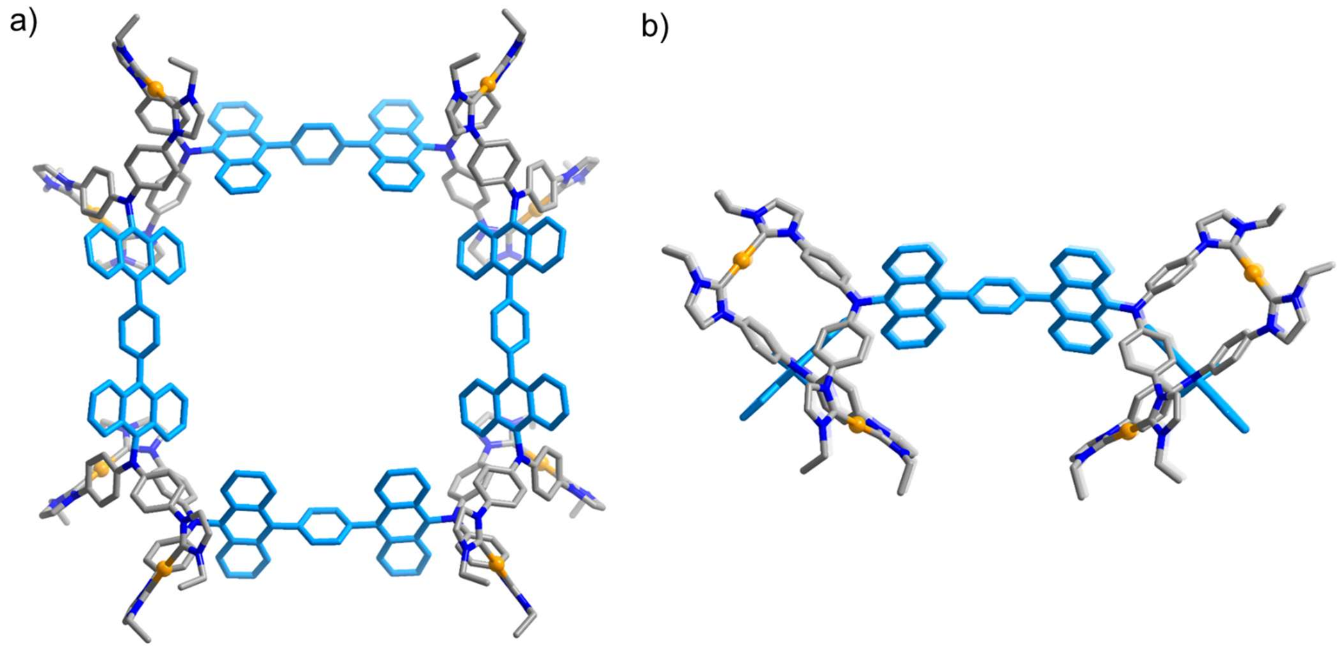

	C_{anth}– C_{anth}– C_{anth}– C_{anth}– C_{anth}– C_{anth}
C_{phenyl}–N–C_{phenyl}	79.76°, 82.61°

Figure S54. a and b) Two views of the complex octacation $[Au_8($**1d**$)_4]^{8+}$ in $[Au_8($**1d**$)_4](PF_6)_8$.

8. Density Functional Theory Calculations

All calculations were performed with the Gaussian(R) 09 program optimizer.[S5] The theoretical approach is based on the framework of density functional theory (DFT).[S6,S7] The geometry optimizations were performed at B3LYP level using LANL2DZ basis set for the Ag element and the 6-31G* basis set for all of the other atoms.

9. References

S1. K. S. Kisel, T. Eskelinen, W. Zafar, A. I. Solomatina, P. Hirva, E. V. Grachova, S. P. Tunik, I. O. Koshevoy, *Inorg. Chem.* **2018**, *57*, 6349−6361.

S2. O. V. Dolomanov, L. J. Bourhis, R. J. Gildea, J. A. K. Howard, H. Puschmann, *J. Appl. Cryst.* **2009**, *42*, 339−341.

S3. L. J. Bourhis, O. V. Dolomanov, R. J. Gildea, J. A. K. Howard, H. Puschmann, *Acta Cryst.* **2015**, *A71*, 59−75.

S4. G. M. Sheldrick, *Acta Cryst.* **2015**, *C71*, 3−8.

S5. M. J. Frisch, G. W. Trucks, H. B. Schlegel, G. E. Scuseria, M. A. Robb, J. R. Cheeseman, G. Scalmani, V. Barone, B. Mennucci, G. A. Petersson, H. Nakatsuji, M. Caricato, X. Li, H. P. Hratchian, A. F. Izmaylov, J. Bloino, G. Zheng, J. L. Sonnenberg, M. Hada, M. Ehara, K. Toyota, R. Fukuda, J. Hasegawa, M. Ishida, T. Nakajima, Y. Honda, O. Kitao, H. Nakai, T. Vreven, J. A. Montgomery Jr., J. E. Peralta, F. Ogliaro, M. J. Bearpark, J. Heyd, E. N. Brothers, K. N. Kudin, V. N. Staroverov, R. Kobayashi, J. Normand, K. Raghavachari, A. P. Rendell, J. C. Burant, S. S. Iyengar, J. Tomasi, M. Cossi, N. Rega, N. J. Millam, M. Klene, J. E. Knox, J. B. Cross, V. Bakken, C. Adamo, J. Jaramillo, R. Gomperts, R. E. Stratmann, O. Yazyev, A. J. Austin, R. Cammi, C. Pomelli, J. W. Ochterski, R. L. Martin, K. Morokuma, V. G. Zakrzewski, G. A. Voth, P. Salvador, J. J. Dannenberg, S. Dapprich, A. D. Daniels, Ö. Farkas, J. B. Foresman, J. V. Ortiz, J. Cioslowski, D. J. Fox, Gaussian, Inc. Revision E.01. Wallingford CT, 2013.

S6. P. Hohenberg, W. Kohn, *Phys. Rev. B*. **1964**, *136*, B864−B871.

S7. W. Kohn, L. Sham, *J. Phys. Rev*. **1965**, *140*, A1133−A1138.
